# Exploiting Co(III)-Cyclopentadienyl Complexes To Develop Anticancer Agents

**DOI:** 10.1021/acs.inorgchem.3c03696

**Published:** 2024-03-19

**Authors:** João Franco Machado, Sandra Cordeiro, Joana N. Duarte, Paulo J. Costa, Paulo J. Mendes, Maria Helena Garcia, Pedro V. Baptista, Alexandra R. Fernandes, Tânia S. Morais

**Affiliations:** † Centro de Química Estrutural, Institute of Molecular Sciences, Faculdade de Ciências, Universidade de Lisboa, Campo Grande, 1749-016 Lisboa, Portugal; ‡ Associate Laboratory i4HB − Institute for Health and Bioeconomy, NOVA School of Science and Technology, NOVA University Lisbon, 2819-516 Caparica, Portugal; § UCIBIO, Departamento de Ciências da Vida, Faculdade de Ciências e Tecnologia, Universidade Nova de Lisboa, 2819-516 Caparica, Portugal; ∥ BioISI − Instituto de Biosistemas e Ciências Integrativas, Faculdade de Ciências, Universidade de Lisboa, 1749-016 Lisboa, Portugal; ⊥ LAQV-REQUIMTE (Polo de Évora), Escola de Ciências e Tecnologia, Universidade de Évora, R. Romão Ramalho 59, 7000-671 Évora, Portugal

## Abstract

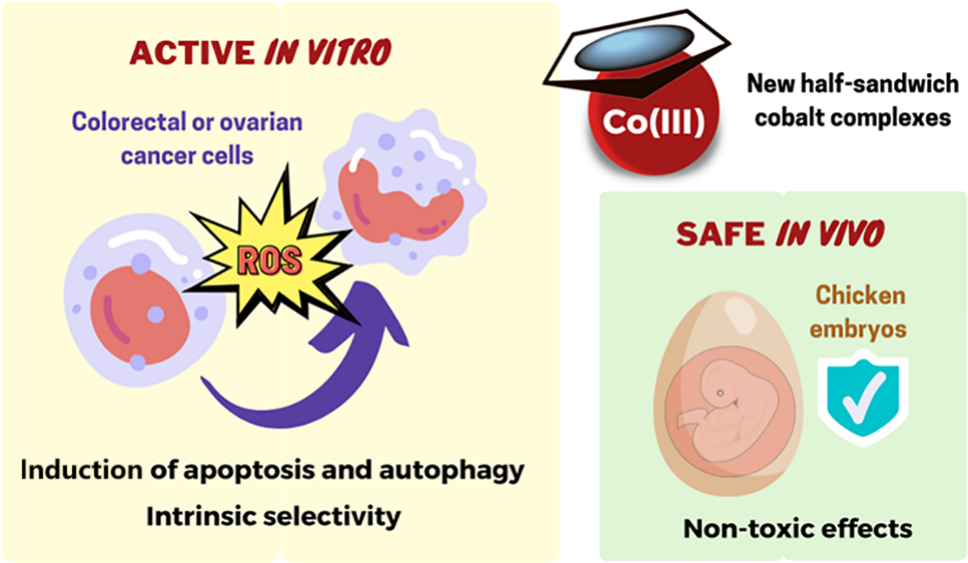

In recent years, organometallic complexes have attracted much attention as anticancer therapeutics aiming at overcoming the limitations of platinum drugs that are currently marketed. Still, the development of half-sandwich organometallic cobalt complexes remains scarcely explored. Four new cobalt(III)-cyclopentadienyl complexes containing N,N-heteroaromatic bidentate, and phosphane ligands were synthesized and fully characterized by elemental analysis, spectroscopic techniques, and DFT methods. The cytotoxicity of all complexes was determined in vitro by the MTS assay in colorectal (HCT116), ovarian (A2780), and breast (MDA-MB-231 and MCF-7) human cancer cell lines and in a healthy human cell line (fibroblasts). The complexes showed high cytotoxicity in cancer cell lines, mostly due to ROS production, apoptosis, autophagy induction, and disruption of the mitochondrial membrane. Also, these complexes were shown to be nontoxic in vivo in an ex ovo chick embryo yolk sac membrane (YSM) assay.

## Abstract 2

First half-sandwich complexes of Co(III) bearing an unsubstituted cyclopentadienyl and containing a monodentate phosphane and bidentate N,N-heteroaromatic ligands as potential anticancer agents.

## Introduction

Cancer remains a major burden in society worldwide. Despite the much progress achieved in oncology over the last decades, medical science has not succeeded in providing an efficient solution to cure cancer. The serendipitous discovery of the anticancer properties of cisplatin has fueled the development of several metal complexes for cancer therapy.^1−3^ In this regard, the anticancer properties of ruthenium, osmium, rhodium, copper, gold, iridium, and titanium metal complexes have been explored.^2,4−12^ Among them, ruthenium complexes have drawn great attention due to their broad spectrum of activity, low toxicity, and lower drug resistance than cisplatin.^13−17^ Particularly, half-sandwich organometallic Ru-arene complexes have preceded other anticancer organic and inorganic drugs due to their equilibrium between hydrophilicity and lipophilicity which could facilitate cellular uptake. Indeed, several examples of ruthenium(II)-(η^6^-C_6_H_6_) complexes showed promising in vitro and in vivo antitumor properties against various tumor types, even in cisplatin-resistant cell lines.^6,18−23^ We have been focusing on developing new half-sandwich cationic complexes derived from the Ru(II)-(η^5^-C_5_H_5_) fragment as prospective anticancer agents. These Ru(II)-(η^5^-C_5_H_5_) derivatives, containing mono/bidentate heteroaromatic and phosphane ligands, have proven to be strong cytotoxic agents against a large range of cancer cell lines in vitro,^5,24−31^ revealing in some cases an antimetastatic behavior in vivo.^32,33^ Nonetheless, and despite these important advances in ruthenium complexes, these compounds still present some toxicity.

Alternative therapeutic approaches based on designing new complexes containing bioessential metals may lead to lower systemic toxicity than conventional metallodrugs and those complexes may reach the specific biological targets more easily. Cobalt is an essential trace element for humans, playing a vital role in several critical biological processes, being mainly found in the form of vitamin B12 (cobalamin).^34,35^ Since humans have developed mechanisms to overcome cobalt overload, cobalt is thus less toxic than nonessential metals like platinum and ruthenium. In the past decade, a large number of cobalt complexes have been developed for therapeutic applications, demonstrating its therapeutic potential.^35−41^ Despite the extensive work developed on cobalt complexes, and besides vitamin B12, there are so far no reports on the usage of cobalt compounds in general clinical use. However, a Co(III) imine complex (Doxovir) has already reached phase II clinical trials for antiviral treatment.^42,43^ Several in vitro studies suggest that cobalt complexes may become promising anticancer agents.^44−49^ The great diversity of physicochemical properties of cobalt complexes, such as its accessible redox behavior and rich photochemistry, results in complexes with different cytotoxic modes of action.^35,44,46,48^

The chemistry of cobalt complexes for therapeutic applications has been essentially dominated by classical coordination complexes. In contrast, the design and development of half-sandwich organometallic cobalt complexes remains scarcely studied and exploited in this context.^35^ Our interest has focused on the exploitation of the “piano-stool” Co-cyclopentadienyl complexes as prospective anticancer agents. Notice that half-sandwich metal complexes with *N*,*N*-heteroaromatic ligands such as 2,2′-bipyridine or 1,10-phenantroline show promising anticancer and/or antimetastatic properties, both in vitro and in vivo, and are strong alternatives to current chemotherapeutic metallodrugs such as cisplatin.^5,32,50^ Notably, the ruthenium complex [RuCp(PPh_3_)(bipy)][CF_3_SO_3_] (TM34) has been shown to surpass the cytotoxicity of cisplatin against several cancer cell lines (breast, ovarian, prostate, leukemia) owing to a different mode of action that might overcome drug resistance to cisplatin by targeting the cell membrane, Golgi apparatus, and mitochondria instead of the nuclear DNA.^26,28^ Encouraged by our previous results obtained with ruthenium compound TM34, we decided to explore an analogous family of Co(III)-cyclopentadienyl (CoCp) complexes with *N*,*N*-heteroaromatic bidentate ligands. Herein, we report the unprecedented synthesis and structural characterization of a new family of organometallic Co(III)(η^5^-C_5_H_5_) complexes of general formula [CoCp(PPh_3_)(NN)][(CF_3_SO_3_)_2_], where NN represents 2,2′-bipyridine, 4,4′-dimethyl-2,2′-bipyridine, 1,10′-phenanthroline, and 5-amino-1,10′-phenanthroline ligands. To the best of our knowledge, these are the first examples of half-sandwich complexes of Co(III) bearing an unsubstituted cyclopentadienyl and containing a monodentate phosphane and bidentate *N,N-*heteroaromatic ligands ever reported in the literature. After a complete characterization using experimental and computational techniques, their cytotoxicity was determined in vitro by the MTS assay in colorectal, ovarian, and breast human cancer cell lines, and in healthy fibroblasts. The cellular uptake was studied by ICP-AES. The determination of cell death mechanism and ROS production is presented and discussed. Also, studies using an ex ovo chick embryo yolk sac membrane (YSM) assay^51,52^ were performed to evaluate the potential of these complexes to modulate the angiogenic process and their toxicity in vivo as angiogenesis is part of the invasion/metastization process in cancer but is also important for tumor growth and maintenance as it allows tumor cells to receive nutrients (glucose, oxygen, etc.).^52^

## Experimental Methods

### Materials and Methods

All chemicals and solvents were of analytical reagent grade and used without further purification except dichloromethane and *n*-hexane, which were purified immediately before use with an MBraun SPS-800 solvent purification system. All manipulations involving air-free syntheses and purifications were carried out under a dinitrogen atmosphere using Schlenk techniques. The starting material [CoCp(CO)I_2_] was synthesized according to previously reported procedures.^53^ NMR spectra were recorded in acetone-*d*_6_ at probe temperature using a Bruker Avance 400 spectrometer on 400.13 MHz (^1^H NMR), 100.62 MHz (APT-^13^C{^1^H} NMR) or 161.97 (^31^P{^1^H} NMR). Chemical shifts (δ) are reported in parts per million (ppm), downfield from solvent peaks considering internal Me_4_Si (0.00 ppm) in the ^1^H and ^13^C spectra or referred from external 85% H_3_PO_4_ in the ^31^P spectra. All NMR resonances were unambiguously assigned using bidimensional complementary experiments (COSY, HSQC, and HMBC). Abbreviations: s = singlet; d = doublet; t = triplet; m = multiplet; *J* = coupling constant. FT-IR spectra (4000–400 cm^–1^) were recorded in KBr pellets at room temperature, using a Shimadzu IRAffinity-1 spectrophotometer, only the most significant bands are cited in the text. Electronic spectra (233–900 nm) were recorded in dichloromethane (1 × 10^–5^–1 × 10^–3^ M) at room temperature, using a Jasco V-560 spectrometer and quartz cuvettes with a 1 cm optical path. Elemental analyses were performed at Laboratório de Análises at Instituto Superior Técnico, using a Fisons Instruments EA1108 system. Data acquisition, integration, and handling were made resorting to the software package EAGER-200 (Carlo Erba Instruments).

### Syntheses of the complexes

The new cobalt(III) complexes of general formula [Co(η^5^-C_5_H_5_)(PPh_3_)(NN)][(CF_3_SO_3_)_2_], where NN represents the *N*,*N*-bidentate ligands 2,2′-bipy (**1**), phen (**2**), Me_2_bipy (**3**), or NH_2_phen (**4**), were synthesized following the general procedure: to a stirring solution of [CoCp(CO)I_2_] (0.20g, 0.5 mmol) in acetonitrile (10 mL) was added dropwise a solution of the desired ligand (0.5 mmol) and AgCF_3_SO_3_ (0.26 g, 1.0 mmol) in acetonitrile (10 mL). The mixture was stirred at 0 °C for 10 min protected from light, turning from black to purple-red. The formation of an AgI precipitate was also observed. The solution was then cannula-filtrated into a stirring solution of triphenylphosphane (0.13 g, 0.5 mmol) in acetonitrile (10 mL). The resulting mixture was stirred at 0 °C for 5 min, turning to orange-red. The solvent was evaporated under vacuum, and the residue was washed with diethyl ether (2 × 10 mL) and cold dichloromethane (1 × 5 mL). The products were recrystallized by slow diffusion of dichloromethane/*n*-hexane or methanol/diethyl ether, affording crystalline products.

#### Data for [Co(η^5^-C_5_H_5_)(PPh_3_)(bipy)][(CF_3_SO_3_)_2_] (**1**)

Recrystallized from dichloromethane/*n*-hexane; orange crystals; yield: 56%.

^1^H NMR [(CD_3_)_2_CO, Me_4_Si, 400.13 MHz] δ/ppm: 9.72 [d, 2, H_1_, ^3^*J*_HH_ = 5.00 Hz]; 8.38 [d, 2, H_4_, ^3^*J*_HH_ = 7.62 Hz]; 8.29 [t, 2, H_3_, ^3^*J*_HH_ = 7.29 Hz]; 7.80 [m, 2, H_2_]; 7.67 [m, 3, H_para_(PPh_3_)]; 7.54 [m, 6, H_meta_(PPh_3_)]; 7.29 [t, 6, H_ortho_(PPh_3_), ^3^*J*_HH_ = 9.07 Hz]; 6.62 [s, 5, H_*Cp*_]. APT-^13^C{^1^H} NMR [(CD_3_)_2_CO, Me_4_Si, 100.62 MHz] δ/ppm: 159.32 [C_1_]; 158.36 [C_5_]; 142.27 [C_3_]; 134.27 [d, C_ortho_(PPh_3_), ^2^*J*_PC_ = 9.60 Hz]; 133.63 [d, C_para_(PPh_3_), ^4^*J*_PC_ = 2.99 Hz]; 130.82 [d, C_meta_(PPh_3_), ^3^*J*_PC_ = 10.89 Hz]; 129.48 [C_2_]; 126.38 [d, C_ipso_(PPh_3_), ^1^*J*_PC_ = 49.42 Hz]; 126.32 [C_4_]; 94.30 [C_Cp_]. ^31^P{^1^H} NMR [(CD_3_)_2_CO, 161.97 MHz] δ/ppm: 41.16 [s, PPh_3_]. FT-IR [KBr, cm^–1^]: 3105–3066 (ν_C–H_, aromatic rings); 1608 (ν_C=N,_ bipy); 1477–1433 (ν_C=C_, aromatic rings); 1259 (νCF_3_SO_3_); 875–696 (δ_C–H_, aromatic rings). Elemental analysis (%) found: C, 48.1; H, 3.2; N, 3.2; S, 8.0. Calculated for C_35_H_28_CoF_6_N_2_O_6_PS_2_·0.5CH_2_Cl_2_ (883.10 g/mol): C, 48.28; H 3.31; N, 3.17; S, 7.26.

#### Data for [Co(η^5^-C_5_H_5_)(PPh_3_)(Me_2_bipy)][(CF_3_SO_3_)_2_] (**2**)

Recrystallized from methanol/diethyl ether; red crystals; yield: 74%.

^1^H NMR [(CD_3_)_2_CO, Me_4_Si, 400.13 MHz] δ/ppm: 9.50 [d, 2, H_1_, ^3^*J*_HH_ = 4.38 Hz]; 8.21 [s, 2, H_4_]; 7.68 [m, 3, H_para_(PPh_3_)]; 7.63 [m, 2, H_2_]; 7.54 [m, 6, H_meta_(PPh_3_)]; 7.28 [t, 6, H_ortho_(PPh_3_), ^3^*J*_HH_ = 7.28 Hz]; 6.56 [s, 5, H_Cp_]; 2.56 [s, 6, H_6_]. APT-^13^C{^1^H} NMR [(CD_3_)_2_CO, Me_4_Si, 100.62 MHz] δ/ppm: 158.22 [C_1_]; 157.84 [C_5_]; 155.31 [C_3_]; 134.35 [d, C_ortho_(PPh_3_), ^2^*J*_PC_ = 9.57 Hz]; 133.53 [d, C_*para*_(PPh_3_), ^4^*J*_PC_ = 2.90 Hz]; 130.77 [d, C_meta_(PPh_3_), ^3^*J*_PC_ = 10.80 Hz]; 130.37 [C_2_]; 127.07 [C_4_]; 126.62 [d, C_ipso_(PPh_3_), ^1^*J*_PC_ = 48.73 Hz]; 93.92 [C_Cp_]; 21.01 [C_6_]. ^31^P{^1^H} NMR [(CD_3_)_2_CO, 161.97 MHz] δ/ppm: 41.05 [s, PPh_3_]. FT-IR [KBr, cm^–1^]: 3120–3082 (ν_C–H_, aromatic rings and methyl groups); 1624 (ν_C=N,_ Me_2_bipy); 1487–1436 (ν_C=C_, aromatic rings and δ_CH3_, Me_2_bipy); 1257 (νCF_3_SO_3_); 875–696 (δ_C–H_, aromatic rings). Elemental analysis (%) found: C, 50.7; H, 3.7; N, 3.2; S, 7.5. Calculated for C_37_H_32_CoF_6_N_2_O_6_PS_2_ (868.69 g/mol): C, 51.16; H, 3.71; N, 3.22; S, 7.38.

#### Data for [Co(η^5^-C_5_H_5_)(PPh_3_)(phen)][(CF_3_SO_3_)_2_] (**3**)

Recrystallized from methanol/diethyl ether; red crystals; yield: 78%.

^1^H NMR [(CD_3_)_2_CO, Me_4_Si, 400.13 MHz] δ/ppm: 10.14 [d, 2, H_1_ + H_10_, ^3^*J*_HH_ = 4.07 Hz]; 8.86 [d, 2, H_3_ + H_8_, ^3^*J*_HH_ = 7.68 Hz]; 8.14 [m, 4, H_2_ + H_5_ + H_6_ + H_9_]; 7.54 [m, 3, H_para_(PPh_3_)]; 7.39 [m, 6, H_meta_(PPh_3_)]; 7.18 [m, 6, H_ortho_(PPh_3_)]; 6.72 [s, 5, H_Cp_]. APT-^13^C{^1^H} NMR [(CD_3_)_2_CO, Me_4_Si, 100.62 MHz] δ/ppm: 160.41 [C_1_ + C_10_]; 148.92 [C_11_ + C_12_]; 140.62 [C_3_ + C_8_]; 133.95 [d, C_ortho_(PPh_3_), ^2^*J*_PC_ = 9.72 Hz]; 133.45 [d, C_para_(PPh_3_), ^4^*J*_PC_ = 2.90 Hz]; 132.38 [C_4_ + C_7_]; 130.41 [d, C_meta_(PPh_3_), ^3^*J*_PC_ = 10.92 Hz]; 128.81 [C_5_ + C_6_]; 128.47 [C_2_ + C_9_]; 125.99 [d, C_ipso_(PPh_3_), ^1^*J*_PC_ = 49.05 Hz]; 94.10 [C_Cp_]. ^31^P{^1^H} NMR [(CD_3_)_2_CO, 161.97 MHz] δ/ppm: 41.25 [s, PPh_3_]. FT-IR [KBr, cm^–1^]: 3082–3076 (ν_C–H_, aromatic rings); 1604 (ν_C=N,_ phen); 1583–1433 (ν_C=C_, aromatic rings); 1261(νCF_3_SO_3_); 873–696 (δ_C–H_, aromatic rings). Elemental analysis (%) found: C, 49.9; H, 3.3; N, 3.3; S, 7.0. Calculated for C_37_H_28_CoF_6_N_2_O_6_PS_2_·0.4CH_2_Cl_2_ (898.63 g/mol): C, 49.99; H, 3.23; N, 3.12; S 7.14.

#### Data for [CoCp(PPh_3_)(NH_2_phen)][(CF_3_SO_3_)_2_] (**4**)

Recrystallized from methanol/diethyl ether; red crystals; yield: 54%.

^1^H NMR [(CD_3_)_2_CO, Me_4_Si, 400.13 MHz] δ/ppm: 10.08 [d, 1, H_1_]; 9.58 [d, 1, H_10_]; 8.99 [d, 1, H_3_, ^3^*J*_HH_ = 8.33 Hz]; 8.35 [d, 1, H_8_, ^3^*J*_HH_ = 8.23 Hz]; 8.07 [m, 1, H_2_]; 7.80 [m, 1, H_9_]; 7.57 [m, 3, H_para_(PPh_3_)]; 7.40 [m, 6, H_meta_(PPh_3_)]; 7.18 [m, 6, H_ortho_(PPh_3_)]; 7.02 [s, 1, H_6_]; 6.68 [s, 5, H_Cp_]; 6.50 [s, 2, H_13_]. APT-^13^C{^1^H} NMR [(CD_3_)_2_CO, Me_4_Si, 100.62 MHz] δ/ppm: 159.83 [C_1_]; 155.09 [C_10_]; 149.43 [C_12_]; 145.83 [C_5_]; 143.05 [C_11_]; 136.63 [C_8_]; 135.53 [C_3_]; 134.43 [C_7_]; 133.83 [d, C_ortho_(PPh_3_), ^2^*J*_PC_ = 9.53 Hz]; 133.27 [d, C_para_(PPh_3_), ^4^*J*_PC_ = 2.74 Hz]; 130.24 [d, C_meta_(PPh_3_), ^3^*J*_PC_ = 10.84 Hz]; 127.76 [C_9_]; 126.83 [C_2_]; 125.95 [d, C_ipso_(PPh_3_), ^1^*J*_PC_ = 49.29 Hz]; 125.10 [C_4_]; 102.88 [C_6_]; 93.86 [C_*Cp*_]. ^31^P{^1^H} NMR [(CD_3_)_2_CO, 161.97 MHz] δ/ppm: 41.61 [s, PPh_3_]. FT-IR [KBr, cm^–1^]: 3250 (ν_N–H,_ NH_2_phen); 3084–3064 (ν_C–H_, aromatic rings); 1645–1598 (ν_C=N_ and δ_N–H_, NH_2_phen); 1519–1435 (ν_C=C_, aromatic rings); 1261 (νCF_3_SO_3_); 867–696 (δ_C–H_, aromatic rings). Elemental analysis (%) found: C, 50.6; H, 3.2; N, 4.8; S, 7.1. Calculated for C_37_H_29_CoF_6_N_3_O_6_PS_2_ (879.67 g/mol): C, 50.52; H, 3.32; N, 4.78; S, 7.29. RP-HPLC: t_R_ = 29.02 min (A: H2O, B: ACN; method 0–1 min: 10%B; 1–18 min: 10–90% B; 18–21 min: 90% B; 21–24 min: 90–10% B; 24–25 min: 10% B); purity: 95,94%.

### Stability Assays by NMR

The stability of the CoCp complexes in aqueous medium was evaluated by ^1^H and ^31^P{H} NMR, using a Bruker Avance 400 spectrometer on 400.13 and 161.97 MHz, respectively. Solutions of the complexes in 90% D_2_O/10% DMSO-*d*_*6*_ were prepared at 2.5 mM and analyzed over 24 h. The spectra were acquired with intervals of 1 h within the first 6 h, followed by a final acquisition by completing 24 h. The samples were kept at room temperature and protected from light in between measurements. Upon competition of the assay, the spectra were analyzed regarding the number, chemical shift, integration, and multiplicity of each ^1^H and ^31^P resonances for each complex.

### Octanol–Water Partition Coefficients (log*P*)

The lipophilicity of the CoCp complexes was estimated by the shake-flask method.^54^ Before the assays, *n*-octanol was vigorously mixed with distilled water for 24 h at room temperature to promote solvent saturation of both phases. After separating the phases, the complexes were dissolved in the *n*-octanol phase to obtain solutions 1.0 × 10^–5^ to 2.5 × 10^–4^ M. Then, each solution was equilibrated with water for 4 h in a mechanical shaker, at a phase ratio of 2 mL *n*-octanol:2 mL water. Afterward, the aqueous and octanol layers were carefully separated by centrifugation (5000 rpm, 10 min), and the UV–vis absorption spectra of the complexes were recorded in the *n*-octanol phase. For each sample, the concentration was determined by using a calibration curve in *n*-octanol. All experiments were performed in triplicate for each complex, being the averages used for calculation purposes. Finally, the values of the partition coefficients were calculated by resorting to the following eq (eq 1):
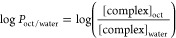
1where log*P*_oct/water_ represents the octanol–water partition coefficient, [complex]_oct_ represents the concentration of the complex in the *n*-octanol phase, and [complex]_water_ represents the concentration of the complex in the water phase.

### DFT and TD-DFT Calculations

All DFT and TD-DFT calculations were performed with Gaussian16^55^ using the PBE0 functional.^56^ Cobalt and phosphorus were represented, respectively, by the LANL2TZ(f) and LANL08(d) basis sets, along with the associated effective core potential.^57,58^ For the other elements, the 6-311G** basis set was employed. The geometry was optimized without symmetry constraints. Using the optimized geometry, TD-DFT calculations were performed, typically requesting the lowest allowed 20 excitations. Solvent effects (dichloromethane) were included by employing the SMD solvation model.^59^ The analysis of the UV–vis spectra and generation of natural transition orbitals cube files was performed with Multiwfn 3.8.^59^

### Cell Culture

Human colorectal carcinoma cell line (HCT116), human breast cancer cell lines (MDA-MB-231 and MCF-7), and normal human primary dermal fibroblasts neonatal (PCS-201-010) cell line were grown in DMEM (Dulbecco’s modified Eagle’s medium) (Invitrogen Corp., Grand Island, NY, USA) supplemented with 10% (v/v) fetal bovine serum and 1% (v/v) antibiotic/antimycotic solution (Invitrogen Corp.). The human ovarian carcinoma (A2780) cell line was cultivated using RPMI (Roswell Park Memorial Institute) medium supplemented as in DMEM medium. The supplemented medium is named a complete medium. Cells were grown in an incubator with a humidified atmosphere at 5% (v/v) CO_2_ and 37 °C. All cell lines were purchased from ATCC (ATCC, American Type Culture Collection).

### Antiproliferative Activity

The evaluation of the cytotoxic potential of complexes **1–4** was obtained through in vitro measurements of the antiproliferative activity of these complexes. Cells were plated in 96-well plates at 7.5 × 10^4^ cells/mL and incubated at 37 °C with 5% (v/v) CO_2_. After 24 h, the culture medium was replaced with fresh medium (100 μL) containing 0.1–50 μM of the different complexes and incubated for 48 h in tumor cell lines or containing 0.1–100 μM of the different complexes and incubated for 48 h in fibroblasts. DMSO 0.1% (v/v) was used as the negative control and cisplatin and doxorubicin (DOX) were used as positive controls (common chemotherapeutic agents) for all cell lines. The fibroblast cell line was used as a control of the cytotoxicity of the complexes for healthy cells. Cell viability was evaluated using Cell Titer 96 Aqueous Non-Radioactive Cell Proliferation Assay (Promega, Madison, WI, USA) using 3-(4,5-dimethylthiazol-2-yl)-5-(3-carboxymethoxyphenyl)-2-(4-sulfophenyl)-2H-tetrazolium inner salt (MTS), as described previously.^60^ In metabolically active cells, enzymes present in the mitochondria catalyze a reaction in which NADPH/NADH is produced. These enzymes reduce the MTS reagent into a brownish product, Formazan, which can be quantified by measuring the absorbance at 490 nm. The quantity of formazan produced is directly proportional to the number of viable cells in the culture. This quantification was executed with a Biorad microplate reader Model 680 (Bio-Rad, Hercules, CA, USA). Half maximal inhibitory concentration (IC_50_) was calculated using GraphPad Prism 6 (GraphPadSoftware). We have calculated the ratio between the IC_50_ of fibroblasts and the IC_50_ of HCT116, A2780, MDA-MB-231, or MCF-7 cell lines for complexes **1–4** that was named herein as the Selectivity index (SI). To ensure the solubility of the complexes in all biological assays, complex solutions were freshly prepared and well solubilized before addition to the cells.

### Inductively Coupled Plasma Atomic Emission Spectroscopy (ICP-AES)

Internalization of the cobalt complexes was evaluated by ICP-AES in the A2780 cell line. For this, the A2780 cell line was seeded in 25 cm^2^ T-flasks at a density of 5 × 10^5^ cells/T-flask and incubated for 24 h at 37 °C with 5% (v/v) CO_2_. After the incubation, the culture media was replaced by fresh medium with 10× the IC_50_ of the different complexes or 0.1% (v/v) of DMSO. As the IC_50_ values in A2780 cells are in the low micromolar range and to ensure ICP-AES sensitivity in terms of limit of detection, the 10× IC_50_ concentrations were used. Cells were then incubated for 3, 6, and 12 h at the same conditions as before and for 12 h at 4 °C. The culture media was recovered to a new tube (supernatant fraction) and cells were detached from the T-flask with 2 mL of TripLE Express (TE) (Gibco by life technologies) and centrifuged at 750 × *g* for 5 min. Then, fresh aqua regia was added to the supernatant tube and to the cellular fractions and incubated overnight at room temperature (RT) in the hood fume. Samples were delivered to Laboratório de Análises/LAQV and the levels of cobalt were evaluated by ICP-AES.

### Cell Fractioning

Internalization of the complexes in different cellular factions (cytosolic, mitochondrial, or nuclear) of A2780 cells was evaluated by using a cell fractionation kit (Abcam). Cells were seeded in a 24-well plate at a density of 2 × 10^5^ cells/mL and incubated for 24 h at 37 °C with 5% (v/v) CO_2_. After the incubation, the culture media was replaced by fresh medium with 10× the IC_50_ of the different complexes or 0.1% (v/v) of DMSO, as for ICP-AES. Cells were then incubated for 12 h at the same conditions as before. The culture media was recovered to a new tube (supernatant fraction) and cells were detached from the T-flask with 250 μL of TE (Gibco by life technologies). Then, we followed the instructions provided by the manufacturer of the cell fractionation kit (Abcam). At the end of the procedure, fresh aqua regia was added to the supernatant tube and to the cellular fractions and incubated overnight at room temperature (RT) in the hood fume. Samples were delivered to Laboratório de Análises/LAQV and the levels of cobalt were evaluated by ICP-AES.

### UV–Visible Spectrum in Biological Medium

UV–visible spectra (220–700 nm) were used to analyze the stability and solubility of complexes **1–4**. Complexes were solubilized in DMSO and later diluted in RPMI medium without phenol red, at final concentrations of 25/50 μM. The spectra were obtained after 0, 24, and 48 h of incubation at 37 °C on the spectrophotometer Shimadzu mini-UV-1240 (Izasa Scientific) with a quartz cuvette with a 1 cm path length. Additionally, a Glutathione (GSH) interaction assay was also performed through the UV–vis spectra (220–700 nm) on the spectrophotometer Evolution 300 UV–vis (Thermo Fischer Scientific, Waltham, MA, USA) with a quartz cuvette with 1 cm path length. As above, RPMI medium without phenol red and FBS was used to dilute the complexes **2–4** at a final concentration of 50 μM with/without 50 μM of GSH. A control solution of GSH was also prepared. All solutions were incubated for 24 h at 37 °C and then analyzed.

### Evaluation of Induction of Apoptosis

The induction of apoptosis was evaluated by flow cytometry in the A2780 cell line, through the Alexa Fluor Annexin V/Dead Cell Apoptosis Kit (ThermoFisher Scientific). A2780 cells were seeded at a density of 2 × 10^5^ cells/well in 6-well plates and incubated for 24 h. After that time, the medium was replaced by medium mixed with complexes **2–4** at their IC_50_. DMSO at 0.1% (v/v) was used as the negative control and 0.4 μM DOX and 5 μM cisplatin were used as positive controls. After a period of 48 h of incubation, cells were washed with PBS 1× and TE was used to detach the cells. The cells were resuspended in annexin-binding buffer 1× and incubated for 15 min at RT with Alexa Fluor 488 annexin V and Propidium Iodide. All samples were analyzed by an Attune Acoustic Focusing Flow Cytometer (ThermoFisher Scientific) and respective software.

### Determination of BAX and BCL-2 Protein Expression by Western Blot (WB)

The quantification of BAX and BCL-2 protein expression in the A2780 cell line incubated with different cobalt complexes in the A2780 cell line was determined by WB. For this, the A2780 cell line was cultivated in 25 cm^2^ T-flasks at a density of 2 × 10^6^ cells/mL and incubated for 24 h in the conditions described above. Cells were incubated for 24 h, and, after that period, the medium was replaced by fresh medium with DMSO 0.1% (v/v) or the IC_50_ c of the complexes **2–4**. After 48 h incubation, the cells were washed and collected using cold PBS 1× and a cell scraper. The samples were centrifuged for 5 min at 700 × *g* and resuspended in fresh lysis buffer (150 mM NaCl, 5 mM ethylenediaminetetraacetic acid (EDTA), 50 mM Tris–HCl at pH 8.0, 2% (v/v) NP-40, 1× phosphatase inhibitors (PhosStop, Roche), 1× Protease inhibitors (complete ULTRA tablets, Mini, easypack, Roche), 1 mM phenylmethylsulfonylfuoride (PMSF) and 0.1% 1,4-dithiothreitol (v/v), DTT) and then storage at −80 °C for 2 h or until required. Cells were submitted to 5 ultrasound cycles and centrifuged at 1000 × *g* for 5 min. The total protein extract (supernatant) was quantified with Pierce Protein Assay Reagent (ThermoFisher Scientific) at 660 nm.

The SDS-PAGE was loaded with 20 ug of protein on 10% polyacrylamide gel and transferred to a 45 μm PVDF membrane (GE Healthcare). A NZYColour Protein Marker II (NZYTech) was used as a molecular weight marker. Each membrane was incubated for 2 h under constant agitation with 5% (w/v) nonfat milk in TBST 1× buffer (50 mM Tris–HCl at pH 7.5, 150 mM NaCl and 0.1% (w/v) Tween 20) and later incubated for 1 h, at RT under constant agitation, with the primary antibodies diluted in 5% nonfat milk in TBST 1×, anti-BAX (1:5000, reference 32503, Abcam) and anti-BCL-2 (1:100; reference B3170, Sigma). After that period, the membranes were washed three times with TBST 1× for 5 min each, under constant agitation, and then incubated with the secondary antibodies, antimouse IgG, horseradish peroxidase HPR-linked antibody (1:3000) and antirabbit IgG, HPR-linked antibody (1:2000) (both from Cell Signaling Technology, USA), for identification of BCL-2 and BAX protein, respectively.

The membranes were incubated with WesternBright ECL subtract (Advansta) for 5 min in the dark and the film was exposed to the membranes in a dark room. Membranes were later incubated two times with stripping buffer (0.1 M glycine, 20 mM magnesium acetate, 50 mM KCl at pH 2.0) during 10 and 20 min, respectively, with agitation and then incubated with anti-β-actin (1:5000; reference A5441, Sigma) as a control to normalize the expression results of the proteins. Protein quantification was made by densitometry with ImageJ software.

### Evaluation of the Mitochondrial Membrane Potential (ΔΨM)

ΔΨM was evaluated in the A2780 cell line using the JC-1 Mitochondrial Membrane Potential Assay Kit (Abnova, Taipei, Taiwan). Cells were seeded at a density of 2 × 10^5^ cells/well in 6-well plates and incubated for 24 h. Posteriorly, the medium was replaced by medium with DMSO 0.1% (v/v), vehicle control, 0.4 μM DOX and 5 μM cisplatin, positive controls, or IC_50_ of the complexes **2–4**. After an incubation of 48 h, the cells were washed with PBS 1×, detached with TE, and incubated with JC-1 in RPMI medium without phenol red + 5% (v/v) FBS for 20 min at 37 °C. The cells were then resuspended in the same medium and the ΔΨM was evaluated with the Attune acoustic focusing flow cytometer (ThermoFisher Scientific) and respective software.

### Evaluation of the Levels of Caspase 8 Activity

The A2780 cell line was seeded in 25 cm^2^ T-flasks at a density of 2 × 10^6^ cells/T-flasks. After 24 h incubation, at the same conditions as described previously, the medium was replaced by medium with DMSO 0.1% (v/v), 0.4 μM DOX, a 5 μM cisplatin or IC_50_ of the complexes **2–4,** and incubated for 48 h. After that, cells were detached using cold PBS 1× and a cell scraper and centrifuged at 500 × *g* for 5 min. Then, we followed the instructions provided by the manufacturer of the Caspase-8 assay kit (Abcam). The data was obtained by measuring the absorbance of each sample at 400 nm and each value was normalized to the value of the DMSO sample.

### Evaluation of Autophagy Induction

The autophagy induced by complexes **2–4** was evaluated using the Autophagy Assay Kit (ab139484, Abcam, Cambridge, United Kingdom) according to the manufacturer’s instructions. For this, the A2780 cell line was seeded at a density of 2 × 10^5^ cells/well in 6-well plates and incubated for 24 h at the conditions mentioned before. Posteriorly, the medium was replaced by medium with DMSO 0.1% (v/v) (vehicle control), 0.4 μM DOX and 5 μM cisplatin, positive controls, and the IC_50_ of the complexes **2–4**. Cisplatin can induce autophagy through the activation of the BECN1 and DOX high cytotoxicity, which also activates this process.^61−63^ The positive control Rapamycin at 500 nM was incubated for only 15 h. After 48 h incubation, the cells were washed with PBS 1×, detached with TE, and treated with the Autophagy detection kit (Abcam, Cambridge, UK) according to the manufacturer’s instructions. The evaluation of autophagy induction was done through the Attune acoustic focusing flow cytometer (ThermoFisher Scientific) and respective software.

### Production of Reactive Oxygen Species (ROS)

The production of ROS was evaluated in the A2780 cell line, seeded in 6-well plates at a density of 2 × 10^5^ cells/well, and incubated for 24 h at the same conditions as described above. After that, the medium was replaced by medium with DMSO 0.1% (v/v), vehicle control, 0.4 μM DOX, 5 μM cisplatin, and 22.2 μM TBHP (as positive controls) or IC_50_ of the complexes **2–4** and incubated for 48 h. Later, cells were washed with PBS 1×, detached from the wells with TE, and incubated with 10 mM of 2′,7′-dichlorodihydrofluorescein diacetate (H_2_DCF-DA) (ThermoFisher Scientific, Waltham, MA, USA) in PBS 1× for 20 min at 37 °C. The data were obtained through an Attune acoustic focusing flow cytometer (ThermoFisher Scientific) and respective software.

### Cell Cycle Progression

The interference of the copper complexes **2–4** in the cell cycle progression was evaluated in A2780 cells, seeded in 6-weel plates, with a density of 2 × 10^5^ cells/well, and incubated for 8 h at the same conditions as described above. After that incubation time, the cells were synchronized in early S-phase with a 2 mM thymidine solution (Sigma, St. Louis, USA) for a 16 h period. Media with thymidine was replaced by fresh media and, after 8 h, another 2 mM thymidine solution was added to the wells (double thymidine block) and incubated for 16 h. The cell medium was replaced with IC_50_ concentrations of complexes **2–4**, DMSO 0.1% (v/v), vehicle control, 0.5 μM DOX, or 5 μM cisplatin (positive controls). Cells were incubated for 9, 18, or 24 h, and, at each hour, the cells were treated with TE and centrifuges at 650 × *g* for 5 min at 4 °C. The pellet was resuspended in PBS 1× and centrifuged at 3000 × *g* for 5 min at 4 °C. Later, the pellet was resuspended in 100 μL of PBS 1×, and 1 mL of a solution of 80% (v/v) ethanol was gently added to the cells. Cells were stored at 4 °C for at least 12 h and then centrifuged for 10 min at 7500 × *g* at 4 °C, treated with 250 μL of 50 μg/mL RNase A, and incubated for 30 min at 37 °C. After incubation, 100 μL of PI (25 μg/mL) was added to the cells as well as 650 μL of PBS 1×. The DNA content was evaluated on an Attune Acoustic Focusing Flow Cytometer (ThermoFisher Scientific) and results were analyzed by the respective software.

### *Escherichia coli* Culture and Plasmid DNA Extraction

*E. coli* transformed with pUC18 was inoculated onto a plate of LB-agar medium (Luria–Bertani) (Applichem, Darmstadt, Germany) supplemented with ampicillin (100 μg/mL) (Bioline, London, UK) and incubated for 24 h at 37 °C. Then, the *E. coli* cells were inoculated in liquid LB medium, supplemented with ampicillin (100 μg/mL) for 24 h at 37 °C. After incubation, the plasmid DNA (pDNA) was extracted by using the *Kit NZYSpeedy Miniprep*, following the instructions provided by the manufacturer.

### Evaluation of the Interaction with pDNA

The extracted pDNA (100 ng) was incubated with 5, 25, 50, and 100 μM concentrations of complexes **2–4**, with DMSO 0.1% or only pUC18. The solutions were prepared in 5 mM Tris–HCl, 50 mM NaCl, pH 7.02, buffer, and incubated for 24 h at 37 °C. A control of pDNA cleaved with *Hind III* endonuclease was also used. Electrophoresis was performed in 0.8% (w/v) agarose gel (NZYtech) in TAE buffer at 70 V constant voltage for 1 h. Electrophoresis gel image was acquired in the Gel Doc EZ Imager (Bio-Rad).

### Determination of the DNA Cleavage Mechanism

The pDNA (100 ng) was incubated with 25 μM of complexes **2–4**, in the absence or presence of 50 μM of NaN_3_, 4 units of catalase from the bovine liver (2000–5000 U/mg, Sigma-Aldrich, Spain), and 25 mM of D_2_O. The solutions were prepared in 5 mM Tris–HCl, 50 mM NaCl, pH 7.02, buffer, and incubated for 24 h at 37 °C. A control of pDNA cleaved with *Hind III* endonuclease was also used. Electrophoresis was performed in 0.8% (w/v) agarose gel (NZYtech) in TAE buffer at 70 V constant voltage for 1 h. Electrophoresis gel image was acquired in Gel Doc EZ Imager (Bio-Rad).

### Single-Cell Gel Electrophoresis Assay (Comet Assay)

A2780 cells were seeded in 6-well plates with a density of 1 × 10^6^ cells/well and incubated for 24 h in an incubator with a humidified atmosphere at 5% (v/v) CO_2_ and 37 °C. After that time, the medium was replaced by a medium with 10× IC_50_ concentrations of complexes **2–4**, 0.1% (v/v) DMSO as vehicle control or 0.05% (v/v) H_2_O_2_. The complexes and DMSO were incubated at 37 °C for 12 h and H_2_O_2_ for 30 min at RT. Cells were harvested with a cell scraper and solutions with 1 × 10^5^ cells/mL in PBS were prepared. It was withdrawn 10 μL from the solutions and added to 90 μL of 1.5% (w/v) low melting point agarose in 1× PBS and dropped on the slides prepared previously with a coating of 1.5% (w/v) agarose (normal melting point agarose) in 1× TAE buffer. After drying at 4 °C for 15 min, the slides were dipped into lysis solution (450 mM NaCl, 3.72% EDTA, 5 mM Tris; to which 10% (v/v) DMSO and 1% (v/v) Triton-X were added just before use) for 1 h at 4 °C, followed by their being dipped for 40 min into cold electrophoresis solution (1 mM EDTA, 300 mM NaOH, pH 13). Electrophoresis was performed at 4 °C for 30 min at 25 V using a Sub-Cell model 96 apparatus (Bio-Rad). Afterward, slides were placed in 0.1 M Tris–HCl buffer (pH 7.5) at 4 °C for 15 min for neutralization, followed by 15 min in methanol at 4 °C and drying at 37 °C. For visualization of comets, slides were hydrated with distilled water at 4 °C for 30 min, stained with 20 μL of GelRed working solution (3×), and covered with new coverslips. The slides were observed under a Ti–U Eclipse inverted microscope (Nikon Instruments, Japan) and analyzed by CometScore version 2.1 (TriTek). About 100 cells per sample were analyzed. The percentage of DNA in the tail was used as a measure of the total DNA strand breakage.

### *Calf Thymus* DNA (CT-DNA) Binding Assays

*Calf Thymus* DNA (CT-DNA) binding assay was performed through the UV–vis spectra from 230 to 700 nm on the spectrophotometer Evolution 300 UV–vis (Thermo Fischer Scientific, Waltham, MA, USA). It was used a filtered buffer (5 mM Tris–HCl, 50 mM NaCl, pH 7.02) for the dilution of 0–50 μM of CT-DNA and 25 μM of the complexes **2–4**. Control solutions of CT-DNA, CT-DNA + DMSO, and DMSO were also prepared. All solutions were incubated for 24 h at 37 °C and then analyzed. CT-DNA concentration in base pairs had been determined at 260 nm in a NanoDrop2000 spectrophotometer (Thermo Scientific), using an extinction coefficient of 6600 M^–1^ cm^–1^. The dilution effect because of the addition of the DNA solution was corrected and the affinity constants were calculated according to eq 2:
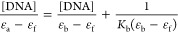
2where [DNA] is the concentration of CT-DNA (per nucleotide phosphate), ε_a_ = Abs/[complex], ε_f_ is the extinction coefficient for the free complex, and ε_b_ is the extinction coefficient for [Co(η^5^-C_5_H_5_)(PPh_3_)(NN)][(CF_3_SO_3_)_2_] when fully bound to DNA. DNA concentration (expressed as the molarity of phosphate groups) was determined with a NanoDrop 2000 spectrophotometer assuming ε_260_ = 6600 M^–1^ cm^–1^. A melting profile of 10 μM CT-DNA and 10 μM CT-DNA with 10 μM of the complexes **2–4**, was also performed. Evagreen dye was added to all samples after a 1h 30 min incubation of CT-DNA with the complexes at 37 °C. Melting curves were analyzed in a Corbet RotorGene 6000 (Biorad, Spain) by a first incubation step at 37 °C for 5 min followed by a ramp from 37 to 95 °C with 90 s hold in the first step and 5 s hold in the next steps with green acquisition.

### Cell Migration Assay

Fibroblasts were seeded in 24-well plates at a density of 4 × 10^5^ cells/mL and grown in the same conditions as described previously until a confluent monolayer was obtained. The medium was changed by a medium with DMSO 0.1% (v/v) or the IC_50_ of complexes **2–4** and a scratch was made on the surface of each well. Images of the Fibroblasts were captured right after the exposure (0 h) to the complexes and after 24 h of incubation at 37 °C and 5% (v/v) CO_2_ in a humidified atmosphere, using CytoSMART Lux2 Live Cell Imager (Axion biosystems, USA) and Ti–U Eclipse Inverted microscope (Nikon Instruments, Japan). Images were analyzed with the ImageJ MRO Wound Healing Tool or with the scratch assay measurement of the CytoSMART cloud portal for the Lux system.

### Ex Ovo Chick Embryo Yolk Sac Membrane (YSM) In Vivo Assay

Ex ovo chick embryo yolk sac membrane (YSM) in vivo assay was performed as previously described.^51,52^ First, the chicken embryos were incubated for 24 h at 37 °C, 5% (v/v) CO_2_, and humidified atmosphere, to stabilize the embryos, and after that, it was placed O-rings with a solution containing DMSO 0.1% (v/v), as the negative control, or the IC_50_ of complexes **2–4** in PBS 1× in the middle. The embryos were incubated for 48 h at 37 °C and images were captured after 0, 24, and 48 h. Newly formed blood vessels were manually counted, via ImageJ software.

The ex ovo YSM assay fulfills the Directive 2010/63/EU of the European Parliament to protect animal models for scientific purposes.

### Statistical Analysis

GraphPad Software (GraphPad Prism version 8.01 for Windows, GraphPad Software, La Jolla, CA, USA, www.graphpad.com) was used to perform the statistical analysis of the data performing a comparison using one-way ANOVA with a confidence interval of 95%.

## Results and Discussion

### Design and Synthesis of New CoCp Complexes

**Scheme 1:**
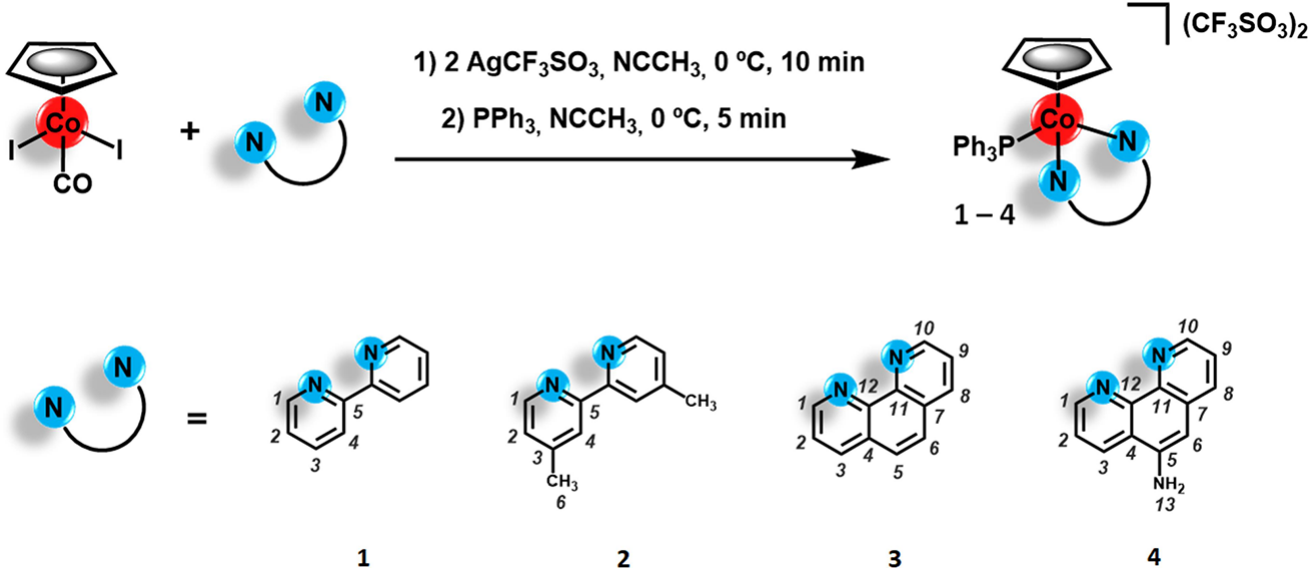
Synthesis of the New Complexes of General Formula [CoCp(PPh_3_)(NN)][(CF_3_SO_3_)_2_] (Cp = η^5^-C_5_H_5_; NN = *N*,*N*-Heteroaromatic Ligands): **1** (NN = **bipy** = 2,2′-Bipyridine); **2** (NN = **Me**_**2**_**bipy** = 4,4′-Dimethyl-2,2′-bipyridine); **3** (NN = **phen** = 1,10′-Phenanthroline); **4** (NN = **NH**_**2**_**phen** = 5-Amino-1,10′-phenanthroline)

A new family of dicationic Co(III)-cyclopentadienyl (CoCp) complexes with N,N-heteroaromatic bidentate ligands of general formula [CoCp(PPh_3_)(NN)][(CF_3_SO_3_)_2_], where NN = 2,2′-bipyridine (bipy; complex **1**); 4,4′-dimethyl-2,2′-bipyridine (Me_2_bipy, complex **2**); 1,10′-phenanthroline (phen; complex **3**); or 5-amino-1,10′-phenanthroline (NH_2_phen, complex **4**), was synthesized according to Scheme 1. The four new complexes were prepared from [CoCp(CO)I_2_] by σ-chelation of the *N*,*N*-heteroaromatic ligands after iodide abstraction with silver trifluoro methanesulfonate, followed by σ-coordination of the triphenylphosphane coligand. The reactions were carried out in situ, in acetonitrile at 0 °C for 10 and 5 min, respectively, achieving the desired products after recrystallization by slow diffusion of diethyl ether in dichloromethane or methanol at room temperature. The CoCp complexes **1**–**4** were obtained in moderate-high yields (54 to 78%), in the same order of magnitude as the analogous RuCp complexes with triphenylphosphane and bidentate *N*,*N*-heteroaromatic ligands.^29,30^

The solid-state FT-IR spectra of complexes **1**–**4** showed the characteristic ν_C–H_ and ν_C=C_ stretching bands of the aromatic rings of the cyclopentadienyl and *N*,*N*-heteroaromatic ligands in the ranges of 3120–3064 and 1583–1433 cm^–1^, respectively. All complexes also showed bands at 1261–1257 cm^–1^ characteristic of the CF_3_SO_3_^–^ counterions and at 1624–1604 cm^–1^ assigned to ν_C=N_ vibrations of the ligands. Complex **2** also presented the ν_C–H_ and δ_CH3_ vibrations of the methyl groups of Me_2_bipy in the ranges of 3120–3082 and 1487–1436 cm^–1^, respectively. For complex **4**, the vibrations of the amine group of the NH_2_Phen ligand were observed at values 3250 cm^–1^ (ν_N–H_) and 1645–1598 cm^–1^ (δ_N–H_).

The new complexes were also characterized by ^1^H, APT-^13^C{H}, and ^31^P{H} NMR experiments complemented by 2D experiments (COSY, HMQC, and HMBC) in acetone-*d*_6_ at room temperature; see Scheme 1 for the atom labeling of the coordinated *N,N*-heteroaromatic ligands. The ^1^H NMR spectra of the free ligands were also recorded under the same experimental conditions for comparison. As a general trend, a deshielding was observed on the ^1^H NMR signals of the *N*,*N*-heteroaromatic ligands upon coordination to the metal center in all complexes, a feature particularly perceived in the *ortho* protons (Δ_δ_ H_1_/H_10_ = 0.83–1.04 ppm), which is a strong evidence of a successfully σ-coordination. The assignment of the H_1_/H_10_ protons is consistent with the relatively low values of their vicinal coupling constants (^3^*J*_HH_ = 4.07–5.00 Hz), which are in good agreement with the literature values for ^3^*J*_HH_ of protons adjacent to electronegative atoms such as nitrogen.^64^ Moreover, deshielding of the ^1^H NMR signals of the methyl and amino groups from the ligands of complexes **2** and **4** was also observed upon coordination (Δ_δ_ H_6_ = 0.12 ppm, and Δ_δ_ H_13_ = 0.88 ppm, respectively), being found within their typical range values. The ^1^H resonances of the η^5^-cyclopentadienyl (Cp) ring of all the compounds were also found within the expected range for dicationic half-sandwich cobalt(III) complexes (δ H_Cp_ = 6.56–6.72 ppm).^65,66^ As expected, upon coordination, a deshielding of the Cp ring signal was observed compared to the neutral precursor [CoCp(CO)I_2_] (Δ_δ_ H_Cp_ = 0.61–0.77 ppm), mostly due to the change in the overall charge of the complexes. A π-back-donation effect may also play a role. Indeed, the magnitude of this deshielding is attenuated within the new cobalt complexes following the order **2** > **1** > **4** > **3**, reflecting the σ donor nature of the N,N-hetereroaromatic ligands to the metal center.^67,68^ A general deshielding of the ^1^H resonances of the triphenylphosphane coligand was observed as expected upon its coordination in all four cobalt complexes, particularly for the *para* protons (Δ_δ_ H_para_ = 0.16 to 0.30 ppm). Interestingly, for complexes **3** and **4**, with the phenantroline coligands, a slight shielding of the *ortho* protons (Δ_δ_ H_ortho_ = −0.12 ppm) was also found, suggesting the involvement of the P center in the overall extended π back-donation system. The ^31^P NMR spectra are in good agreement with these findings. An accentuated deshielding of the single sharp signal, attributed to the triphenylphosphane, was observed upon coordination (Δ_δ_ PPh_3_ = 46.69–47.25 ppm), coherent with the strong σ-donor character of this coligand and the overall dicationic charge character of the cobalt complexes. The APT-^13^C{H} NMR spectra are consistent with the results discussed above. However, it is interesting to note that, generally the ^13^C resonances of the complexes generally do not follow the same order as the respective ^1^H signals. Nonetheless, a similar pattern upon coordination as in the ^1^H NMR spectra was observed. Figure S1 exemplifies the general behavior observed in complexes **1**–**4** and depicts the ^1^H and ^31^P{H} NMR spectra of [CoCp(PPh_3_)(bipy)][(CF_3_SO_3_)_2_] (**1**) compared to the spectra of its precursor complex and free ligands.

**Figure 1:**
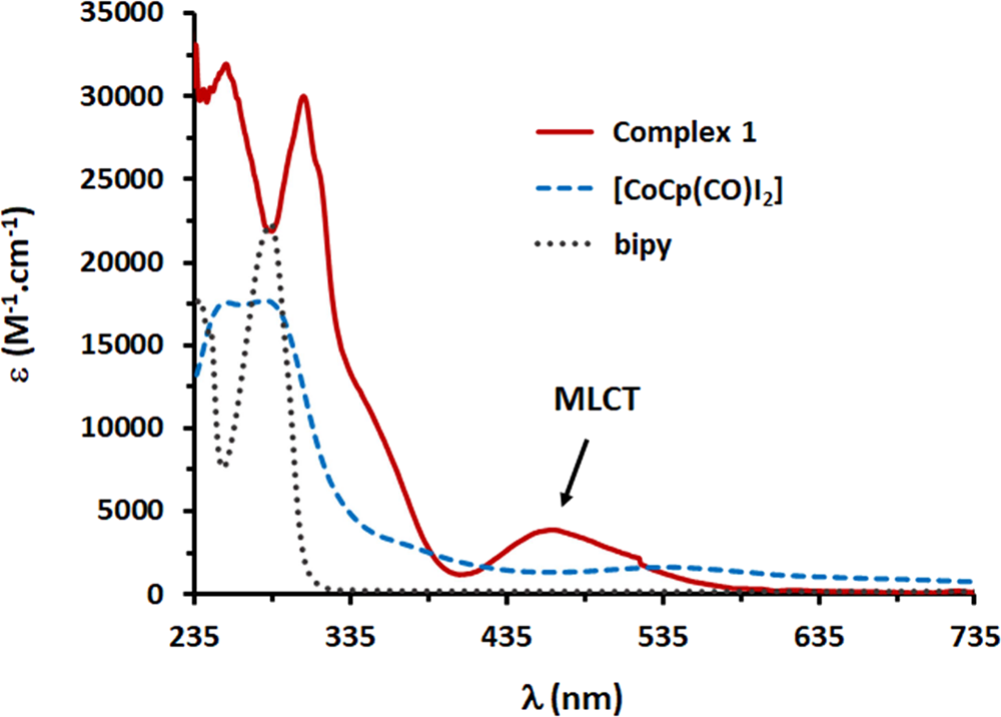
Electronic spectra of [CoCp(PPh_3_)(bipy)][(CF_3_SO_3_)_2_] (**1**, **––**), its precursor [CoCp(CO)I_2_] (**- - -**), and free bipy ligand (^**···**^) in dichloromethane.

The electronic absorption spectra of the four CoCp complexes (**1**–**4**) were acquired in dichloromethane solutions (1 × 10^–5^–1 × 10^–3^ M) at room temperature. For comparison, the electronic spectra of the precursor [CoCp(CO)I_2_] and free ligands were recorded under the same experimental conditions. The corresponding values of absorption maximum are collected in Table S1 of the Supporting Information. Figure 1 depicts the general behavior for this set of complexes, showing the electronic spectra of [CoCp(PPh_3_)(bipy)][(CF_3_SO_3_)_2_] (**1**), its precursor complex, and the free ligand.

All complexes show intense absorption bands in the UV region at 235–370 nm, characteristic of π **→** π* intraligand electronic transitions. Additionally, a band in the visible region (465–467 nm) was also observed in the electronic spectra of all complexes except for **4,** which was found blue-shifted, appearing at 339 nm with ε (molar extinction coefficient) of 3.15 × 10^–3^ M^–1^·cm^–1^, with the same order of magnitude as the ones placed in the visible area (464–467 nm). This band is tentatively assigned as a metal-to-ligand d_Co_ to π*_Cp_ charge transitions (MLCT). Nonetheless, other contributions cannot be ruled out. Further clarification can be found below in the DFT calculations section. For complexes **1** and **2**, coordination of the bipyridyl-derived ligands led to an enhancement of the UV bands at 236–312 nm, assigned to the π **→** π* transitions occurring in the ligand. For complexes **3** and **4**, containing the phenanthroline-derived ligands, no significant alterations were observed in the intensity of the UV bands upon coordination of the *N*,*N*-heteroaromatic ligand. Overall, the new cobalt complexes show similar electronic spectra to their analogous complexes containing other metals, such as ruthenium, although, in these cases, the origin of the MLCT involved the d_Ru_ orbitals to π*_NNligand_.^29,30^ The existence of MLCT bands denoting π interaction of the metal with coordinate ligands whether η^5^-Cp or NN can constitute a piece of information concerning the further design of new compounds since they allow us to fine-tune the electronic flow inside the molecule. In the present cobalt derivatives, the electronic richness at Cp might be inestimable for its functionalization, thus opening a new area of synthesis.

The percentual elemental analyses of all the complexes were also in accordance with the proposed formulations.

### Stability in Aqueous Solution and Estimation of Lipophilicity

**Figure 2:**
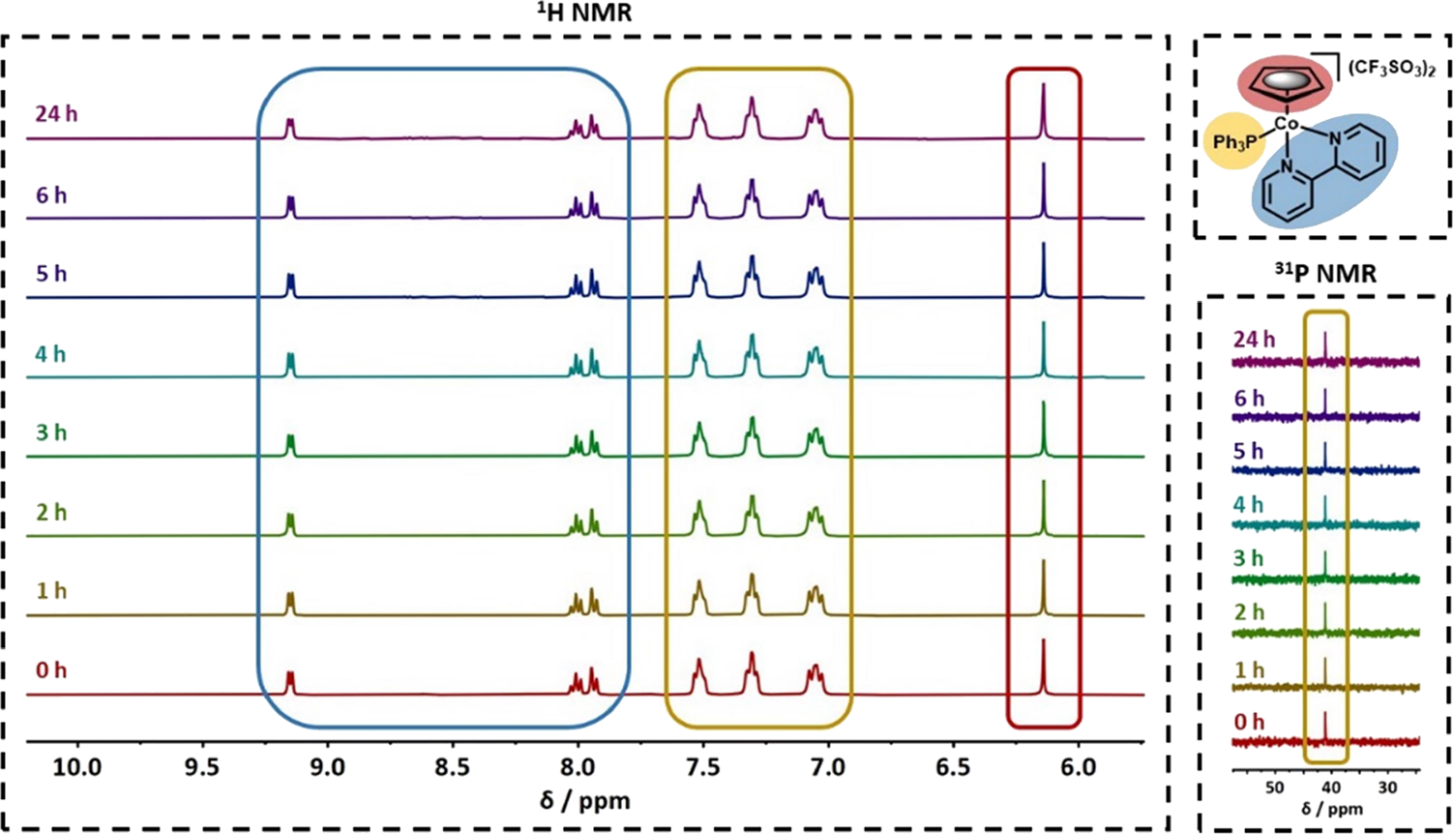
Evaluation of the stability of complex **1** in 90% D_2_O/10% DMSO-*d*_*6*_ solution (2.5 mM) over 24 h, by ^1^H NMR (left) and ^31^P{H} NMR (right).

Determining the stability of organometallic complexes in aqueous solution before in vitro biological studies is a crucial requirement for any substance with a potential biomedical application. Therefore, the stability of the CoCp complexes in aqueous solution (95% D_2_O/5% DMSO-*d*_*6*_) was evaluated over 24 h at room temperature by ^1^H and ^31^P{H} NMR spectroscopy. A small percentage of DMSO was needed to facilitate the dissolution of the complexes as they were only partially soluble in water at the tested concentrations (2.5–3.5 mM). The collected spectra do not display any significant variation over time regarding the number, chemical shift, integration, or multiplicity of the ^1^H and ^31^P resonances. Figure 2 represents the evaluation of the stability of complex **1** over 24 h. All CoCp complexes are stable in aqueous medium for at least 24 h, and thus suitable for further biological studies.

Lipophilicity/hydrophilicity is among the most important physicochemical properties in biomedical applications, as it considerably influences its cytotoxicity/bioactivity, general toxicity, cell membrane/tissue permeability, availability to interact with drug targets, biodistribution, and excretion, as well as drug formulation.^69^ Thus, evaluating the lipophilicity of a drug candidate during the initial phase of the drug development process is a crucial step. Herein, the *n*-octanol/water partition coefficients of the CoCp complexes were estimated by the shake-flask method at room temperature.^54^ All the complexes are slightly hydrophilic, nonetheless, complexes containing bipyridine ligands (log*P***1** = −0.67 ± 0.02; log*P***2** = −0.56 ± 0.02) are less lipophilic than those containing the phenanthroline ligands (log*P***3** = −0.28 ± 0.05; log*P***4** = −0.22 ± 0.03).

### DFT Calculations

**Figure 3:**
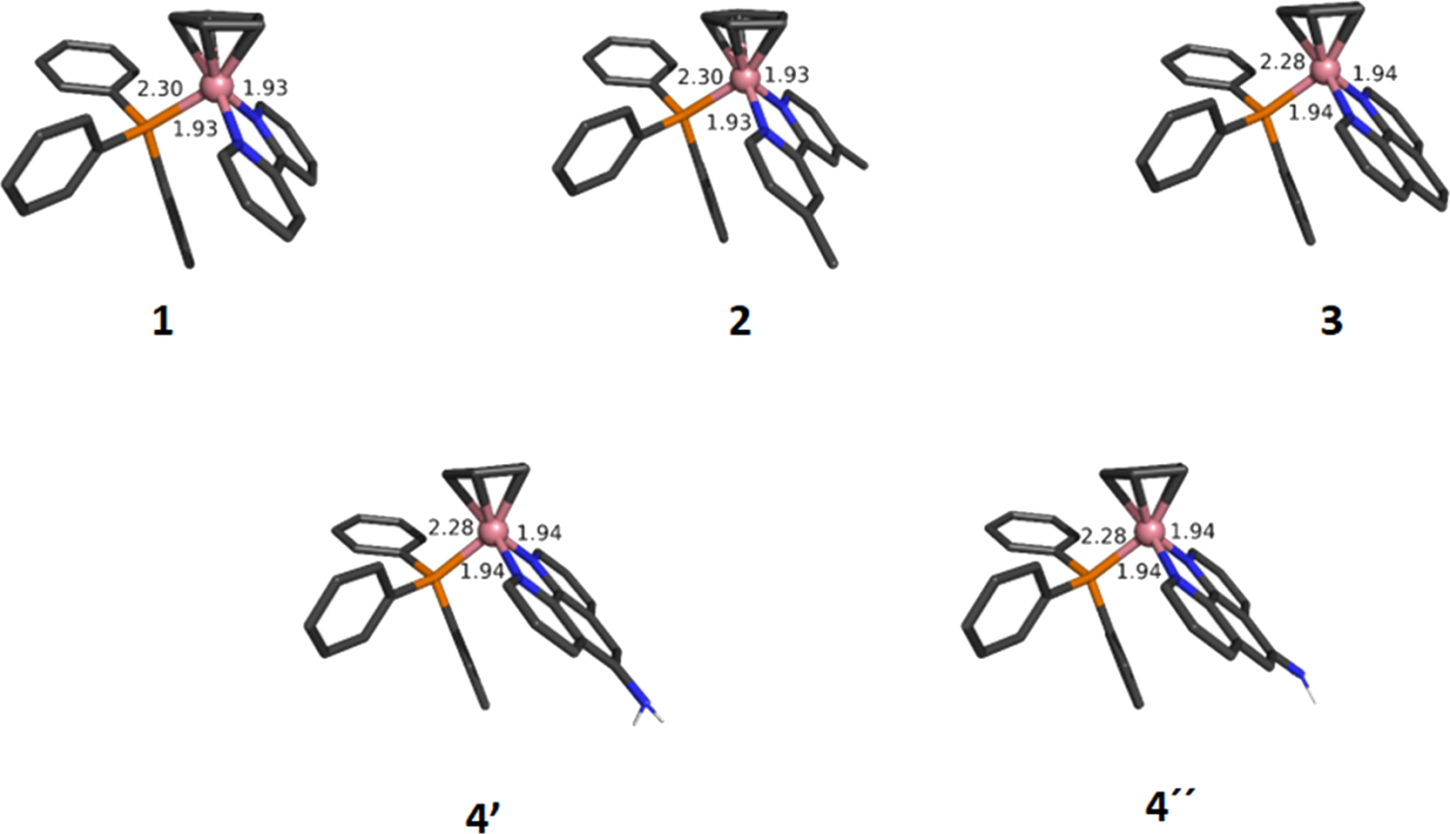
DFT optimized structures of complexes **1**–**4**. For **4**, the possible pair of diastereoisomers was considered (denoted as **4′** and **4″**). Relevant distances are given in Angstrom.

To further elucidate the structural features, owing to the absence of X-ray structures and help in the interpretation of the electronic absorption spectra, DFT and TD-DFT calculations were performed on complexes **1**–**4**. We first started by optimizing their geometries (Figure 3). Notice that due to the pro-chiral nature of the CoCp(PPh_3_) fragment and the single substitution of the NH2phen ligand in **4**, both diastereoisomers were considered (denoted **4′** and **4″**). All complexes feature the classic piano-stool geometry and despite the difference in the NN ligand, no substantial structural changes are observed. Nonetheless, when the NN ligand changes from the bipy-based (**1**–**2**) to phen-based (**3**–**4**), a slight contraction of the Co–P bond is observed (from 2.30 to 2.28 Å) with a concomitant slight elongation of the Co–N bonds (1.93–1.94 Å). This observation is in good agreement with the shielding observed on the H_ortho_ protons of the PPh_3_ attributed to π back-donation d_Co_-to π*_Cp_.

**Figure 4:**
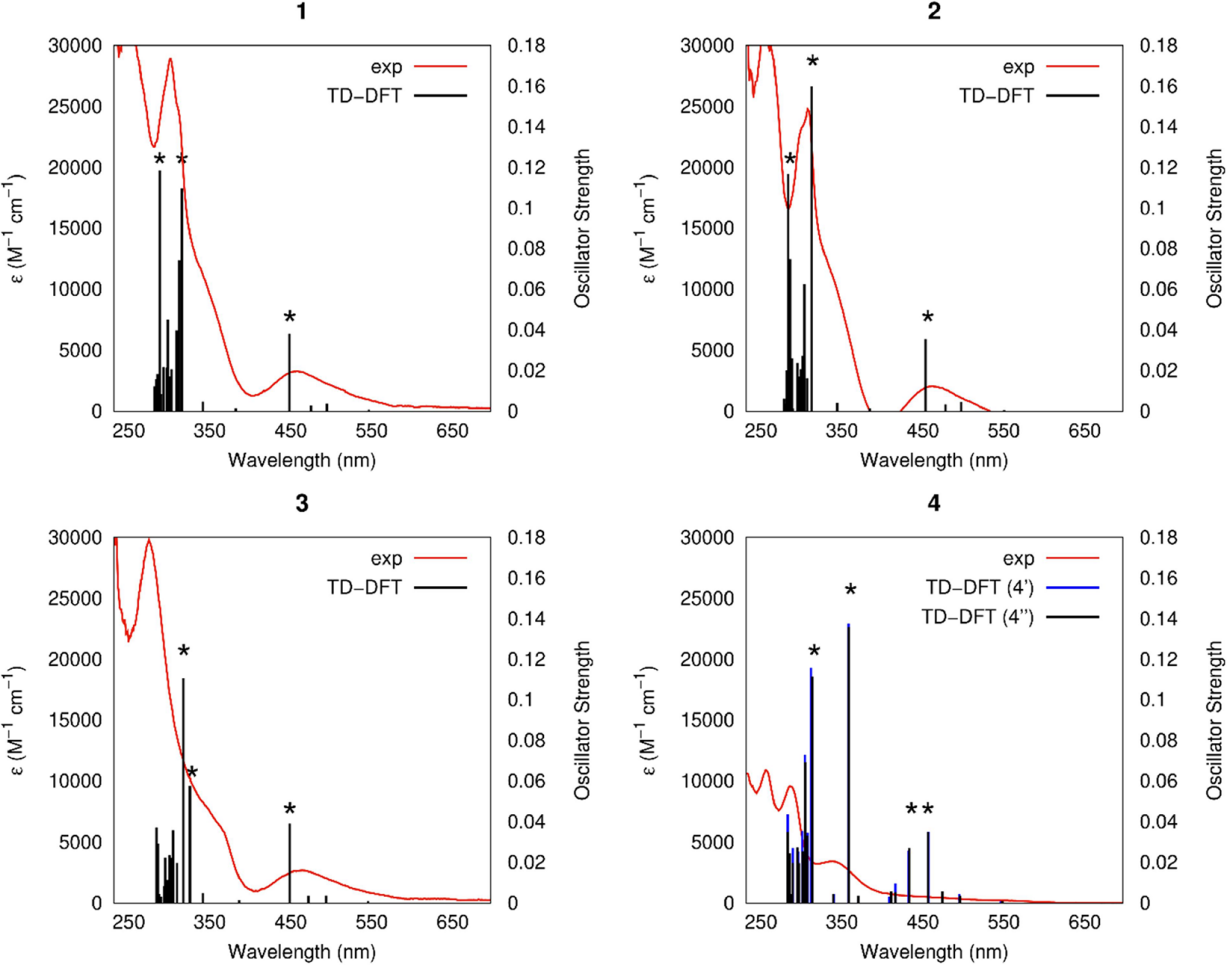
TD-DFT excitations plotted against the UV–visible absorption spectra for complexes **1**–**4**. The excitations reported in Table S2 are highlighted (*). Both calculated and experimental spectra were obtained in dichloromethane.

**Figure 5:**
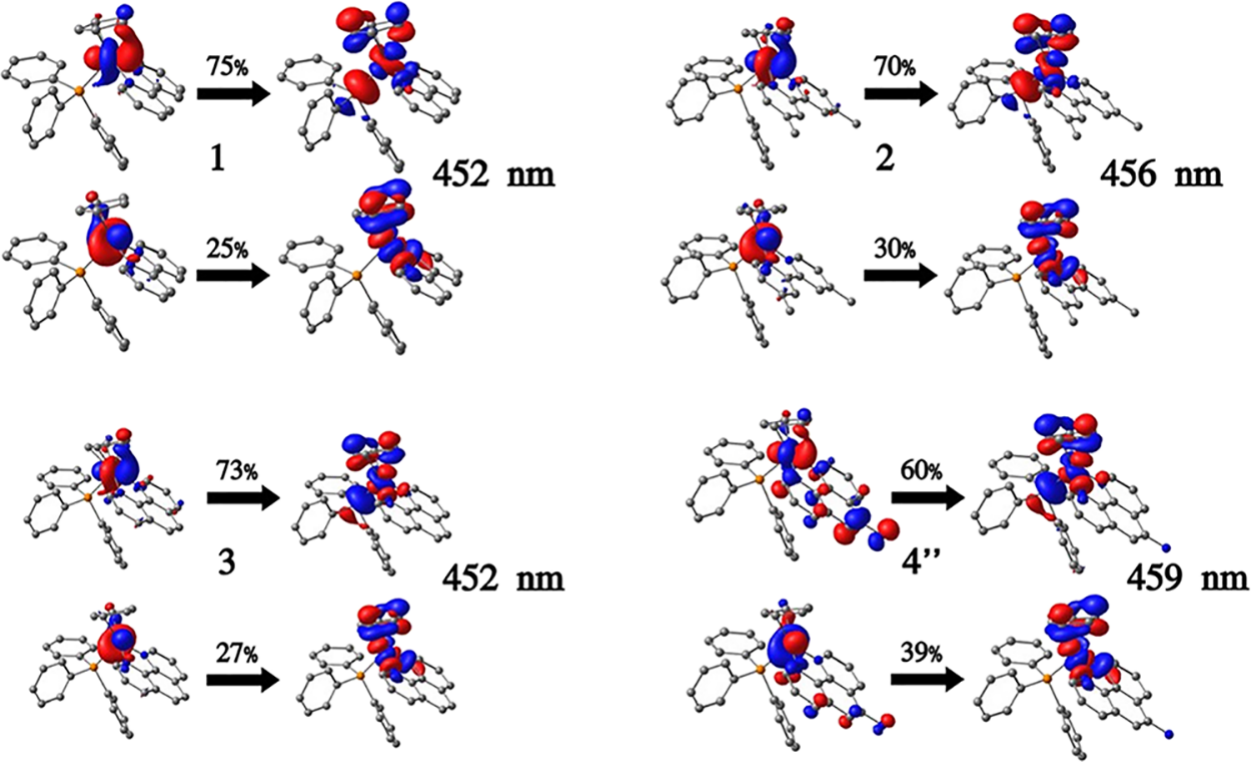
Representative natural transition orbital pairs along with the respective coefficients for the TD-DFT calculated low-energy excitations of complexes **1**-**4**. For **4**, only **4″** is represented for simplicity.

Subsequently, TD-DFT calculations were performed in dichloromethane for all complexes to help in the interpretation of the UV–vis data. Figure 4 shows a superposition of the experimental spectra with the calculated excitations, while the values for the relevant excitations, highlighted with an asterisk, along with their oscillator strengths and composition are listed in Table S2. The superposition of the experimental spectra with the calculated excitations is exceptional for complexes **1**–**3**, while for complex **4**, the agreement is much worse. Despite complex **4** showing a different behavior experimentally, the TD-DFT calculations predict very similar excitation patterns for all complexes, comprising low-energy excitations at >450 nm, which should account for the experimental bands observed in the visible region (465–467 nm) for **1**–**3**, and two main excitations at higher energies. The excitations possess significant contributions from several orbitals (Table S2), therefore, a natural transition orbital (NTO) analysis^70^ was performed to assist in the assignment of the transitions. Figure 5 illustrates the NTO pairs for the calculated low-energy transitions for complexes **1–4**. The excitation possesses a large MLCT character, mainly from d_Co_ to the Cp ligand, though a non-negligible contribution of d–d character is also observed. The π system of the NN ligand does not participate in these low-energy excitations in all complexes. As stated, the TD-DFT calculations predict the existence of an MLCT band for complex **4**, despite this band seeming absent in the experimental spectrum. A possible explanation is that the UV–vis spectrum for complex **4** is blue-shifted, hence, the 339 nm band should correspond to the MLCT transition calculated at 459 nm (see Table S2). Indeed, as mentioned above upon the discussion of the experimental UV–vis spectra of these new CoCp compounds, a band at 339 nm was found for compound **4** with a ε value compatible with a blue-shifted MLCT band (ε = 3.15 × 10^–3^ M^–1^·cm^–1^ in the same order of magnitude as the MLCT bands observed for **1**, **2** and **3** compounds). The NTO pairs for the remainder of relevant high-energy excitations are shown in Figures S2–S5 and confirm the LLCT/ π **→** π* nature of the transitions.

### Antiproliferative Activity

**Figure 6:**
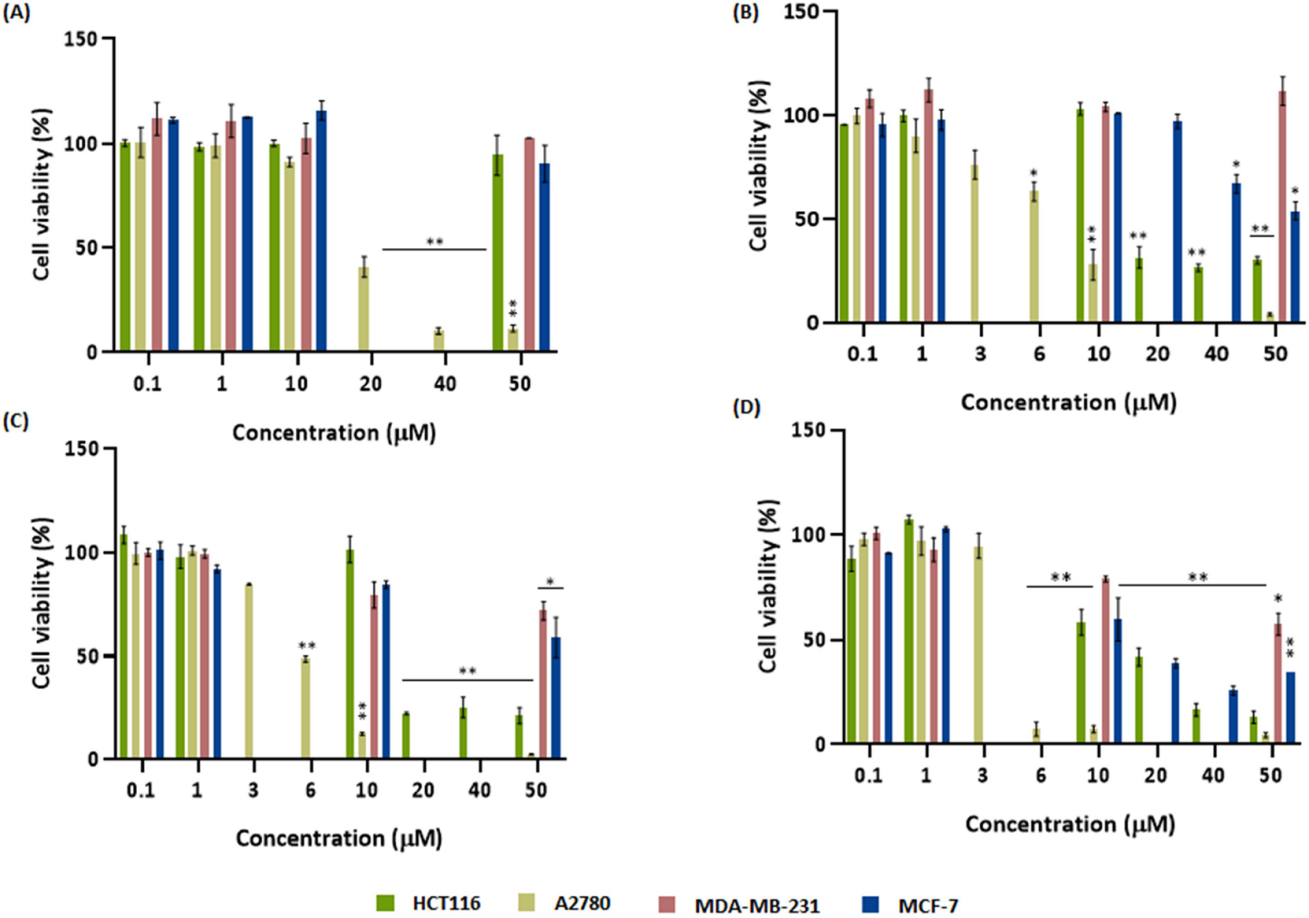
Cellular viability (%) in A2780, HCT116, MDA-MB-231 and MCF-7 after 48 h of exposure to complexes **1** (A), **2** (B), **3** (C), and **4** (D). Data normalized against the control (0.1% (v/v) DMSO) and expressed as the mean ± SEM of at least two independent assays. The symbols ** and * represent *p* < 0.0005 and *p* < 0.05, respectively. Statistical analysis was performed by two-way ANOVA method.

The in vitro cytotoxic potential of complexes **1–4** was evaluated through the CellTiter 96Aqueous Non-Radioactive Cell Proliferation Assay using MTS, as described in the Materials and Methods.^13^ The antiproliferative activity was evaluated through the exposure of A2780 (derived from ovarian carcinoma), HCT116 (derived from colorectal carcinoma), and MDA-MB-231 and MCF-7 (both derived from breast carcinoma) cell lines to 0.1–50 μM to all complexes for 48 h (Figure 6). There is a concentration-dependent reduction of cell viability for all complexes in the A2780 cell line. Interestingly, for complexes **2**–**4**, there is also a concentration-dependent reduction of cell viability for HCT116 and MCF-7 cells, but more effective in HCT116 than in MCF-7 cells (Figure 6B–D). Complexes **3** and **4** were able to induce a concentration-dependent reduction of cell viability for all cell lines including MDA-MB-231, a triple-negative breast cancer cell line (Figure 6C, D). The best way to evaluate the effect of the complexes in the tested cell lines is by calculating their IC_50_ (concentration necessary to inhibit 50% of the cell viability^60^) for each cell line (Table 1). The IC_50_ values show that the cytotoxicity order in the most tumor-sensitive cell line, A2780, is **4** > **2 = 3** > **1**, while for HCT116 is **3** > **4** > **2** > **1** whereas, for MCF-7, only complex **4** is moderately cytotoxic (IC_50_ of 10.2 μM) (Table 1). Despite the slight reduction of cell viability (Figure 6D), complex **4** does not show cytotoxicity for MDA-MB-231 breast cancer cells (IC_50_ > 50 μM) (Table 1). Particularly interesting is the fact that complex **4** shows the lowest relative IC_50_ in the A2780 cell line (4.7 μM) when compared to the other complexes (IC_50_ values of 18.1, 6.1, and 6.2 μM for **1–3**, respectively), demonstrating its high antiproliferative effect in this type of tumor cell line. Remarkably, the IC_50_ of complex **4** (4.7 ± 0.09 μM) is in the same order of magnitude as cisplatin (used as positive control; 1.90 ± 0.20 μM).

**Table 1:** Relative IC_50_ and Selectivity Index (SI) of the Complexes **1–4**, Cisplatin, and DOX in the A2780, HCT116, MDA-MB-231, MCF-7, and Fibroblasts Cell Lines^a^

complex	cell lines	IC_50_ (μM)	SI
**1**	A2780	18.1 ± 0.1	5.5*
	HCT116	>50	–
	MDA-MB-231	>50	–
	MCF-7	>50	–
	fibroblasts	>100	–
**2**	A2780	6.1 ± 0.1	16.4*
	HCT116	15.3 ± 0.2	6.5*
	MDA-MB-231	>50	–
	MCF-7	>50	–
	fibroblasts	>100	–
**3**	A2780	6.2 ± 0.1	16.1*
	HCT116	13.4 ± 0.1	7.5*
	MDA-MB-231	>50	–
	MCF-7	>50	–
	fibroblasts	>100	–
**4**	A2780	4.7 ± 0.1	21.3*
	HCT116	14.1 ± 0.2	7.1*
	MDA-MB-231	>50	–
	MCF-7	10.2 ± 0.1	9.8*
	fibroblasts	>100	–
**doxorubicin**	A2780	0.1 ± 0.1	121
	HCT116	0.5 ± 0.1	24.2
	fibroblasts	12.1 ± 0.2	–
**cisplatin**	A2780	1.90 ± 0.2	4.6
	HCT116	15.6 ± 5.3	0.6
	fibroblasts	8.8 ± 2.9	–

a Data expressed as mean ± SEM of at least three independent assays. * SI values calculated considering that the IC_50_ would be 100 μM (assuming the minimal value); – SI values not calculated. Cytotoxicity data for cisplatin is shown in Supplementary Figure S7.

To understand if the observed cytotoxicity was correlated with the type of ligands, the antiproliferative effect of the ligands was also studied (Supporting Information Figure S6 and Table S3). All free ligands present cytotoxicity in the micromolar (μM) range except for bipy (IC_50_ > 50 μM). The NH_2_phen ligand shows the highest cytotoxicity, particularly in A2780 (IC_50_ = 1.9 ± 0.08 μM) and HCT116 (IC_50_ = 2.3 ± 0.06 μM), followed by Phen (IC_50_ = 2.4 ± 0.05 and 4.1 ± 0.04 μM, for A2780 and HCT116, respectively), which seems to corroborate the higher cytotoxicity of complexes **4** and **3** in those cell lines, particularly in the A2780 cell line (Figure 6, Table 1 and Supporting Information Figure S6 and Table S3). Curiously, complex **2** shows IC_50_ values in A2780 and HCT116 like those presented by complex **3**, although the free ligand (Me_2_bipy) is about 10-fold less cytotoxic than Phen and NH_2_phen ligands. The other ligands (bipy and PPh_3_) and the precursor complex show no or low cytotoxicity in the other cell lines (Supporting Information Figure S6 and Table S3).

The antiproliferative activity of complexes **1–4** was also tested in healthy human cells (fibroblasts) using a range of concentration of 0.1–100 μM (Figure S8). All complexes are not cytotoxic in this healthy cell line (all IC_50_ > 100 μM; Table 1). The selectivity index (SI) of each complex and ligand, based on the ratio IC_50_ in fibroblasts/IC_50_ in a tumor cell line, was determined to evaluate the selectivity of the complex/ligand toward tumor cells. Higher SI indicates higher selectivity of the complex/ligand for a particular tumor cell line.^60,71^ As observed in Table 1, all the evaluated complexes are slightly selective for cancer cells over fibroblasts, with selectivity indexes ranging from 5.0 to 21. It is worth mentioning that complex **4** shows a high selectivity (SI = 21.3) for the A2780 cell and low IC_50_ values for the HCT116 and MCF-7 cell lines (14.1 and 10.2 μM, respectively). Besides these facts, the high SI and low IC_50_ for the A2780 cell line were the decisive factors for choosing this cell line for further biological studies.^72−75^

**Figure 7:**
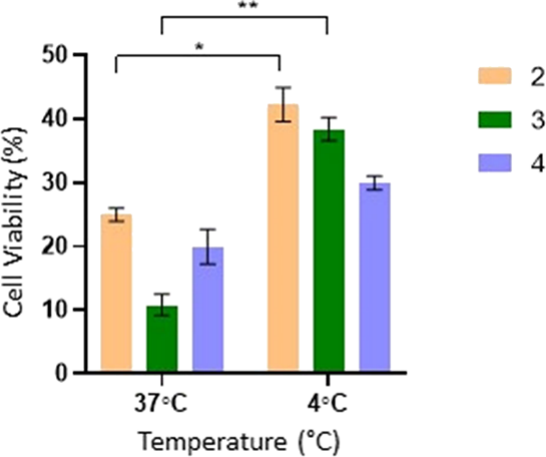
Cellular viability (%) in A2780 after 12 h of exposure to 20× IC_50_ concentrations of complexes **2** – **4** at 37 and 4 °C. Data normalized against the control (0.1% (v/v) DMSO) and expressed as the mean ± SEM of at least two independent assays. The symbols ** and * represent *p* < 0.05 and *p* < 0.5, respectively. Statistical analysis was performed by the two-way ANOVA method.

The internalization of each complex into A2780 cells might correlate with the individual viability data. As such, A2780 cells were incubated with 10× IC_50_ concentrations of complexes **2–4** for 3 h, 6 h, 12 h at 37 °C, and for 12 h at 4 °C, and inductively coupled plasma atomic emission spectrometry (ICP-AES) was used to quantify the % of metal in the cellular fraction (Table 2). There were two major motivations for using these 10× IC_50_ concentrations of complexes: (i) the IC_50_ concentration might be too low considering the limit of detection of the ICP-AES technique; (ii) as the IC_50_ is the relative concentration that induces a 50% reduction of cell viability when exposing cells for 48 h to the complexes and based on our previous data, metal complexes usually enters cells after 3 to 6 h, a higher concentration might be needed to see an effect for shorter time points (3, 6, and 12 h). This last consideration is also a reason why stability in biological media needs to be accessed at least for 24 h (as complexes internalized faster). Indeed, after 12 h of exposure, more than 75% of cobalt is in the cellular fraction for all complexes. Interestingly, the % of internalization of complex **4** is higher compared to the % of internalization of complex **3**, which seems to correlate with its higher cytotoxicity, but when compared to complex **2** both show a similar % of internalization, while complex **4** cytotoxicity is higher (Figure 6 and Table 1). Comparing the results of cobalt internalization after 12 h of exposure to the complexes at 37 and 4 °C (Table 1), it is possible to observe that even at 4 °C more than 45% of complex **2**, 62% of complex **3** and 69% of complex **4** are internalized in the cellular fraction despite the values are lower compared to 37 °C, being complex **2** the one with the lower % of internalization by passive diffusion and more % of complex internalized by an active process compared to **3** and **4**. These results demonstrate that complexes may be internalized by an active transport, as reported by other authors,^76,77^ but they are also able to enter cells by passive diffusion. Considering these results, the % of viability after 12 h of exposure at 37 °C versus 4 °C, was also assessed by exposing A2780 cells to 20× IC_50_ of each complex at those two temperatures. Figure 7 shows that complexes **2** and **3** significantly lose some of their cytotoxic activity at 4 °C when compared to 37 °C. Therefore, analyzing the results obtained in Table 1 and Figure 7 it is possible to say that complexes **2–4** have higher activity and % of internalization at 37 °C due to the contribution of active and passive mechanisms.

**Table 2:** Percentage of Internalization of Complexes **2–4** in the A2780 Cell Line after 3 h, 6 h, and 12 h at 37 °C and for 12 h at 4 °C of Exposure to 10× IC_50_ concentrations

time	complex 2	complex 3	complex 4
3 h at 37 °C	40.6% ± 2.0	49.7% ± 3.5	33.7% ± 2.7
6 h at 37 °C	50.7% ± 3.3	31.9% ± 10.2	43.6% ± 5.8
12 h at 37 °C	92.6% ± 6.5	76.9% ± 15.9	89.3% ± 10.6
12 h at 4 °C	45.5% ± 4.9	62.1% ± 2.3	65.9% ± 3.1

Besides the total cellular fraction, and to understand if complexes were able to accumulate in different cellular organelles, such as mitochondria and nuclei, we have also evaluated, by ICP-AES, the % of cobalt in those subcellular localizations for the three complexes. A2780 cells were exposed to 10× IC_50_ concentrations of the complexes for 12 h and the cell fractioning kit was used to obtain the cytosolic, mitochondrial, and the nuclear fractions (Table 3).

**Table 3:** Percentage of Internalization of Complexes **2–4** in the Different Cellular Fractions (Cytosolic, Mitochondrial, and Nuclear) of A2780 Cells After 12 h of Exposure to 10× IC_50_ Concentrations at 37 °C

cellular fraction	complex 2	complex 3	complex 4
cytosol	36.9% ± 10.4	30.6% ± 2.7	66.4% ± 14.7
mitochondria	17.6% ± 6.5	26.0% ± 11.4	4.5% ± 2.1
nucleus	38.1% ± 7.4	20.3% ± 5.8	18.4% ± 4.9

Table 3 shows that after 12 h, the complexes can accumulate in all the different subcellular fractions, namely, in the nucleus (**2** > **3** > **4**), mitochondria (**3** > **2** > **4**), and cytosol (**4** > **2** > **3**).

Stability tests were also performed to evaluate complexes’ stability/solubility issues when incubated in a cell culture medium over time (Figure S9). Interestingly, complexes **2–4** are relatively stable in a biological medium for 48 h as their characteristic peaks do not change over time. Indeed, for complexes **2–4**, we can observe that a high level of internalization is observed after 3 h, but accumulation continues to increase until 12 h, and at 12h, complexes are found in the cytoplasm and in different cellular organelles (Tables 2 and 3). This means that complexes may internalize very fast to produce their biological effect. The slight changes in solubility between 0 and 24 h (Figure S7) are not sufficient for their lack of biological response due to this fast internalization rate. For complex **1**, the maximum peak at 300 nm slightly disappears and changes from 0 to 24 h, which could indicate some decrease in solubility and changes to the complex (Figure S9). Surprisingly, this was not observed in the NMR studies in water (Figure 2). This result might explain its lower cytotoxicity in A2780 since bipy has revealed good cytotoxic performance when coordinated with other CpM centers (e.g., M = Ru, Fe). The large stability of complexes **2–4** in cell culture medium, high levels of internalization in the cells, high cytotoxicity in A2780 cells, and high SI prompted further biological studies.

### Evaluation of Induction of Apoptosis

**Figure 8:**
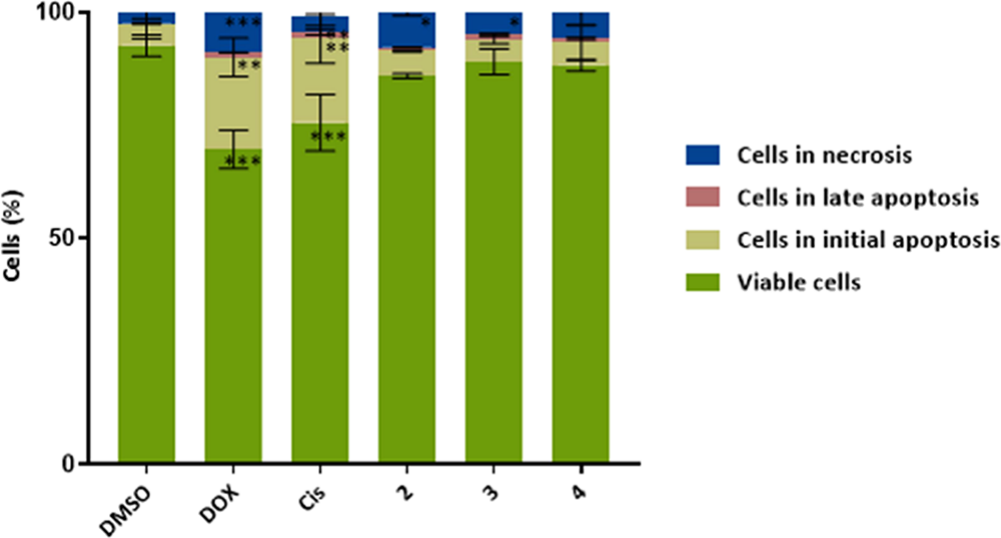
Apoptosis induction in the A2780 cell line exposed to IC_50_ of the complexes **2–4** for 48 h by flow cytometry. Annexin V/PI double staining was used along with DMSO 0.1% (v/v) as negative control (solvent control) and 0.4 μM DOX and 5 μM cisplatin (Cis) as positive controls. Data expressed as the mean ± SD of three independent assays. The symbols ***, **, and * represent *p* < 0.0005, *p* < 0.005 and *p* < 0.05, respectively. Statistical analysis was performed by the two-way ANOVA method.

To fully characterize the antiproliferative potential of the complexes, it is important to understand the type of cell death that is triggered. First, the levels of apoptosis, a type of programmed cell death, and necrosis were determined by flow cytometry using the Annexin V – Alexa fluor 488/PI double staining after 48 h of exposure of A2780 cells to the IC_50_ of each complex **2**–**4** (Figure 8). This allows to distinguishing cells in different stages of the death process, such as early apoptosis (labeled with annexin V-Alexa fluor 488), late apoptosis (labeled with both annexin V-Alexa fluor 488 and PI), necrosis (labeled with PI), from normal viable cells (not labeled) (Figure S10). Exposure to the different complexes showed a slight increase (but without statistical significance) in the percentage of cells in initial apoptosis (5.0, 5.0, and 5.6% for complexes **2**, **3,,** and **4**, respectively), late apoptosis (0.8, 0.9, and 0.9% for complexes **2**, **3**, and **4**, respectively) and necrosis (5.9% for complex **4**) when compared with the control DMSO (4.7, 0.2, and 2.5% for initial apoptosis, late apoptosis and necrosis, respectively) (Figure 8).

The necrosis levels due to exposure to complexes **2** and **3** (7.8 and 6.1%, respectively) are statistically significantly higher when compared to the control DMSO (Figures 8 and S10). Considering total apoptosis levels, we observed 5.8, 5.9, and 6.5% values for complexes **2**, **3**, and **4**, respectively, compared to 4.9% in DMSO. To further confirm these data, we assessed the analysis of the mitochondrial membrane potential (ΔΨM) and the expression of BAX and BCL-2 proteins via Western blot for further information on the dominant apoptotic pathway.

### Evaluation of the Mitochondrial Membrane Potential (ΔΨM)

**Figure 9:**
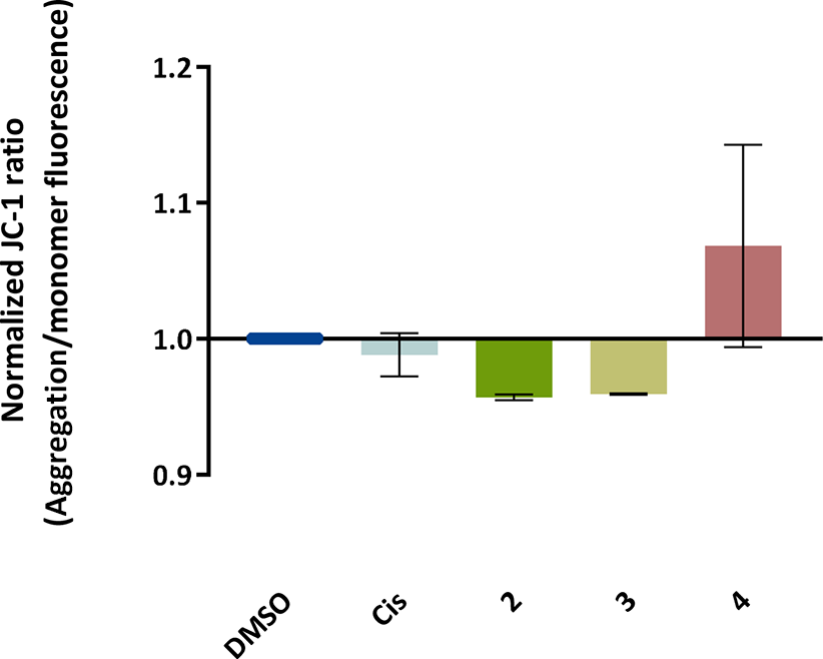
Evaluation of the mitochondrial membrane potential (ΔΨM) changes in the A2780 cell line exposed to IC_50_ of the **2–4** complexes for 48 h by flow cytometry. The JC-1 staining was used along with DMSO 0.1% (v/v) as a negative control (solvent control) and cisplatin (Cis) 5 μM as positive control. Data normalized against the DMSO control and expressed as the mean ± SEM of two independent assays. Statistical analysis was performed by two-way ANOVA method.

The induction of the intrinsic pathway of apoptosis is associated with changes in the mitochondrial membrane potential.^72−75^ Therefore, it is essential to study the effect of the complexes in the mitochondria and their membrane potential. The cationic dye JC-1 was used to evaluate the integrity of the mitochondrial membrane and changes in its potential.^75,78^ This dye naturally accumulates within the mitochondria due to its cationic properties, which enables the formation of aggregates that show red fluorescence (from 532 to 590 nm).^75,78^ When the inner mitochondrial membrane is compromised and has a negative potential (high ΔΨM), the JC-1 dye will leave the mitochondria-originating monomers, with a concomitant change in the dye’s fluorescence from red to green (from 510 to 560 nm).^75,78^ Cells with permeable mitochondria will show a lower red/green fluorescence ratio. The A2780 cell line was exposed to complexes **2–4** for 48 h, and the red/green fluorescence ratios were obtained and represented in Figure 9. The results presented a low (without statistical significance) ΔΨM in the A2780 cell line exposed to complexes **2** and **3** for 48 h, which is in line with data in Figure 8, and Table 3 (higher accumulation in mitochondria), and a low increase in apoptosis compared to control cells. In the case of complex **4**, contrary to complexes **2** and **3**, an increase in ΔΨM was observed, indicating that the membrane potential is hyperpolarized, which may be in line with its lower accumulation in the mitochondria (Table 3). Therefore, based on these data, it is important to use additional methods to confirm if complexes **2** and **3** are triggering an intrinsic apoptotic process and **4** an extrinsic apoptotic process. To further analyze the mitochondria-dependent apoptosis, BAX (a pro-apoptotic protein) and BCL-2 (antiapoptotic protein) expression were assessed. The ratio of BAX/BCL-2 proteins is an excellent marker of the trigger of mitochondria-dependent apoptosis.^79^

### Determination of BAX and BCL-2 Proteins Expression by Western Blot (WB)

**Figure 10:**
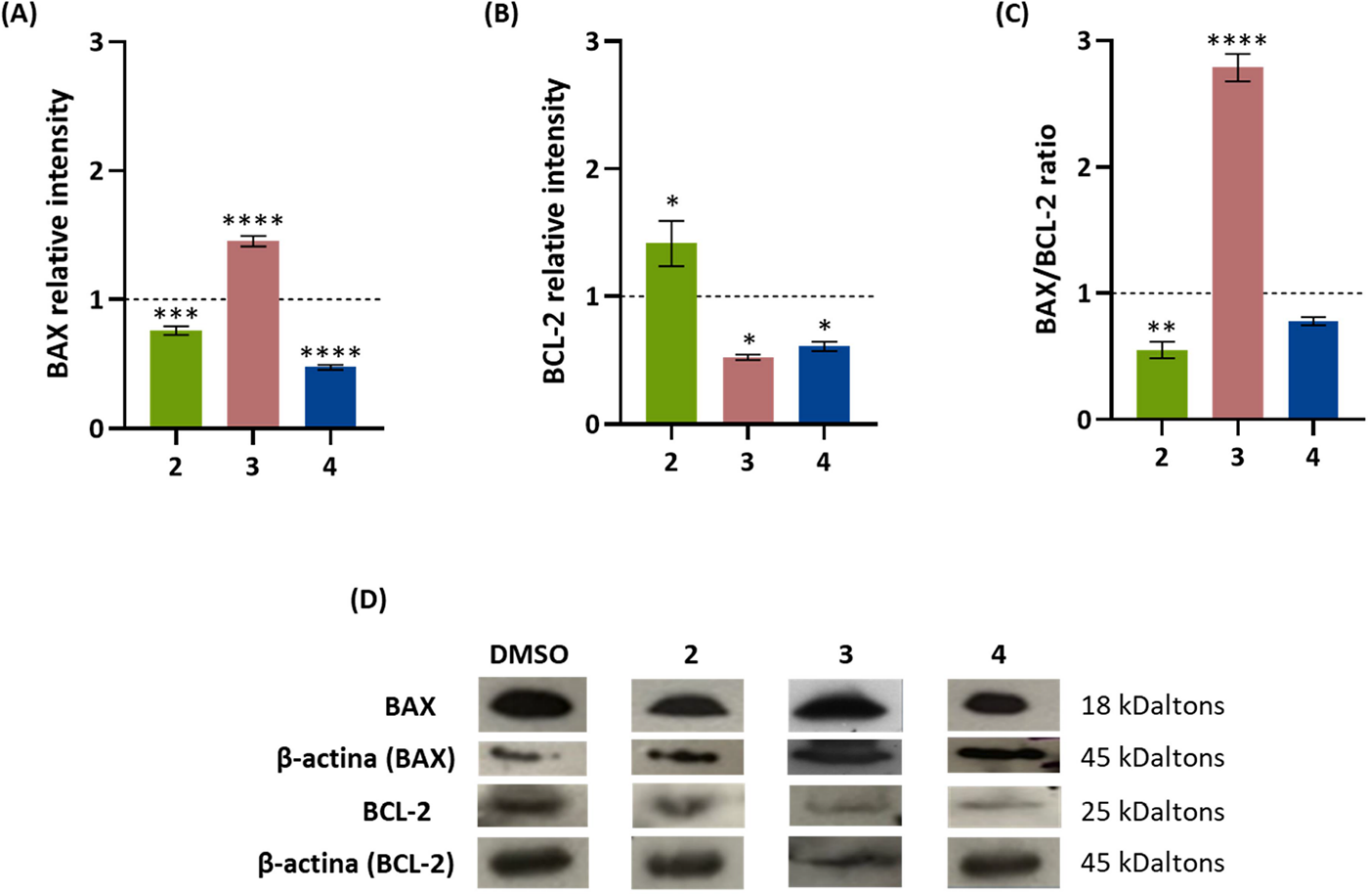
Relative expression of BAX (A) and BCL-2 (B) proteins in the A2780 cell line incubated for 48 h with IC_50_ of the complexes **2–4**. (C) BAX/BCL-2 ratio in A2780 exposed to the different complexes. (D) Western Blot bands used for the quantification of proteins BAX and BCL-2 in A2780 cells after their exposure to complexes 2–4 or DMSO. DMSO 0.1% (v/v) was used as negative control (solvent control). Data normalized against the DMSO control (values represented as a dotted line at the value of *Y* = 1) and expressed as the mean ± SEM. The symbols ****, ***, **, and * represent *p* < 0.0001, *p* < 0.001, *p* < 0.01, and *p* < 0.1, respectively. Statistical analysis was performed by the two-way ANOVA method.

An A2780 cell line was exposed to the IC_50_ concentration of complexes **2**, **3**, and **4** for 48 h, and the levels of those proteins were determined by Western blot (WB) (Figures 10 and S11). Results show a statistically significant increase of the BAX/BCL-2 ratio for complex **3** (Figure 10C), 2.8× higher than the control, meaning that the exposure of A2780 cells to complex **3** leads to a high accumulation in mitochondria after 12 h (Table 3), triggering an increase of BAX/BCL-2 ratio (Figure 10) and depolarization of mitochondria membrane potential (Figure 9), all associated with the induction of mitochondria-dependent apoptosis (intrinsic pathway).^72−75^ On the other hand, complex **2** also accumulates in mitochondria (Table 3) triggering a slight depolarization of its membrane potential (Figure 9), it seems not to be dependent on BAX (Figure 10C). Data for complex **2** (Figures 8–10) might indicate that cell death is being triggered via other pro-apoptotic proteins such as BAK, via an extrinsic signal or via other Type II programmed cell death mechanisms (e.g., autophagy). In what concerns complex **4**, it seems that its lower level of accumulation in mitochondria (compared with **2** and **3**) is not sufficient to induce BAX compared to BCL-2 (BAX/BCL-2 < 1) (Figure 10C) that correlates with the hyperpolarization of mitochondrial membrane (Figure 9). Considering these data, complex **4** might be triggering an extrinsic pathway of apoptosis or Type II programmed cell death mechanism such as autophagy.

It should be noted that these methods to evaluate apoptotic cell death make use of different markers of the process and with different sensitivities. On the one hand, flow cytometry allows the quantification of phosphatidylserine on the external cell membrane, a translocation that is triggered in the early stages of apoptosis (Figure 8), and the internalization/release from mitochondria of a cationic dye that indirectly relates with membrane permeabilization, a process that precedes cytochrome c release to the cytoplasm (Figure 9). The WB data allow a more precise quantification of the BAX and BCL-2 levels, which are involved in the control of mitochondrial membrane permeability (Figure 10).^72−75^ When stimulated by different conditions, BAX suffers a conformational change that enables its translocation to the mitochondrial outer membrane, triggering pore formation and the release of cytochrome C into the cytoplasm.^72−75^ The release of the cytochrome C will lead to the activation of the caspases pathway.^72−75^ To further evaluate if complexes **2** and **4** might induce apoptosis via an external stimulus, caspase 8 activity, which further triggers activation of the apoptotic process but in response to extrinsic stimuli, was measured.^79,80^

### Evaluation of the Levels of Caspase 8 Activity

**Figure 11:**
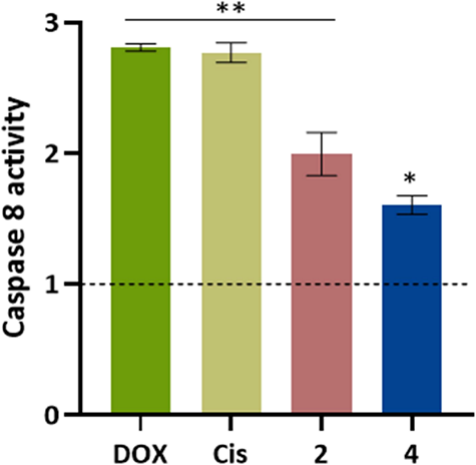
Caspase 8 activity in A2780 cell line incubated with the complexes **2–4** for 48 h. DMSO 0.1% (v/v) was used as negative control (solvent control) and DOX 0.4 μM and cisplatin (Cis) 5 μM as positive controls. Caspase 8 activity was quantified using the caspase 8 assay kit (Abcam). Data normalized against the DMSO control (value represented as a dot line at *y* = 1) and expressed as the mean ± SEM of three independent assays. The symbols ** and * represent *p* < 0.0005 and *p* < 0.005, respectively. Statistical analysis was performed by two-way ANOVA method.

Caspase 8 is activated by apoptotic stimuli from the plasmatic membrane and, subsequently, will cleave several molecules, such as downstream caspases, nuclear proteins and plasma membrane, and mitochondrial proteins.^79,80^ The chromogenic substrate IEDT-pNA, constituted by IETD (Ile-Glu-Thr-Asp) peptide conjugated with the chromophore p-nitroanilide (pNA), is commonly used to quantify caspase 8 activity in total protein extracts.^81^ When the substrate is cleaved by caspase 8, pNA is released and can be quantified by measuring the absorbance at 400 nm.^81^ To evaluate caspase 8 activity induced by the complexes, the A2780 cell line was incubated for 48 h with the IC_50_ of complexes **2** and **4** (Figure 11). Exposure of A2780 to complexes **2** and **4**, show 2× and 1.6× higher activity than that of the control, respectively. Nevertheless, despite significant differences, the values were not as high as those observed for DOX and cisplatin (Figure 11). These results complement those obtained for Annexin V (Figure 8), the expression of BAX and BCL-2 proteins (Figure 10), and the evaluation of the ΔΨM (Figure 9). The evaluation of caspase 8 activity indicated that both complexes **2** and **4** induced apoptosis mostly via an extrinsic pathway (Figure 11).

### Evaluation of Induction of Autophagy

**Figure 12:**
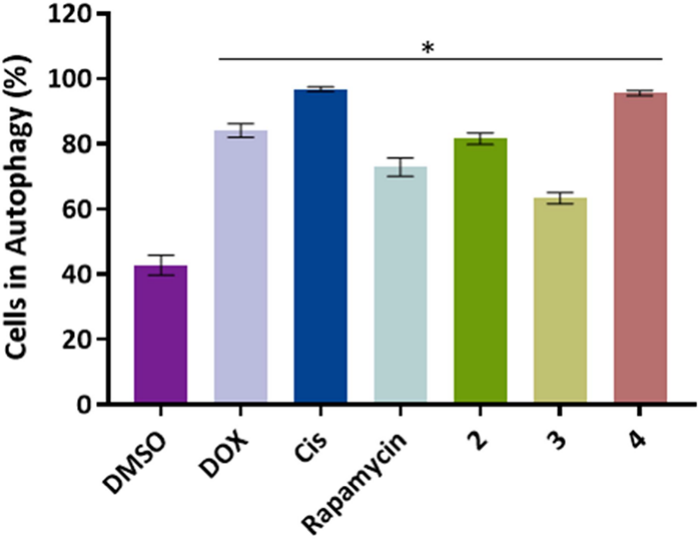
Evaluation of the autophagy induction in A2780 cell line after 48 h of exposure to the IC_50_ of the complexes **2–4** by flow cytometry using the Autophagy assay. DMSO 0.1% (v/v) was used as negative control (solvent control) and DOX 0.4 μM, cisplatin (Cis) 5 μM, and Rapamycin 0.5 μM as positive controls. Data expressed as the mean ± SEM of two independent assays. The symbol * represents *p* < 0.0005. Statistical analysis was performed by two-way ANOVA method.

Besides apoptosis, other types of programmed cell death, such as autophagy, which have been shown to be commonly triggered by different metal complexes.^79,82^ Therefore, the induction of autophagy in the A2780 cell line after exposure to complexes **2–4** was evaluated by the identification of the presence of intracellular autophagosomes^82^ after 48 h exposure to IC_50_ of the complexes (Figure 12). Data show an increase of 1.9×, 1.5×, and 2.2× for complexes **2**, **3**, and **4**, respectively, compared to the control (DMSO 0.1%). The levels of autophagy induced by complexes **2** and **4** (81.5 and 95.5%, respectively) were higher than those for rapamycin (72.93%, positive control), and almost the same as those induced by the DOX (84.1%) and cisplatin (96.7%), which are renowned antitumor agents (Figure 12).

Altogether, these results show a simultaneous induction of apoptotic (via different pathways) and autophagic cell death in cells exposed to complexes **2–4**. To further understand what is triggering this cell death and loss of viability by the presence of the cobalt complexes, intracellular reactive oxygen species (ROS) were measured.

### Production of Reactive Oxygen Species (ROS)

**Figure 13:**
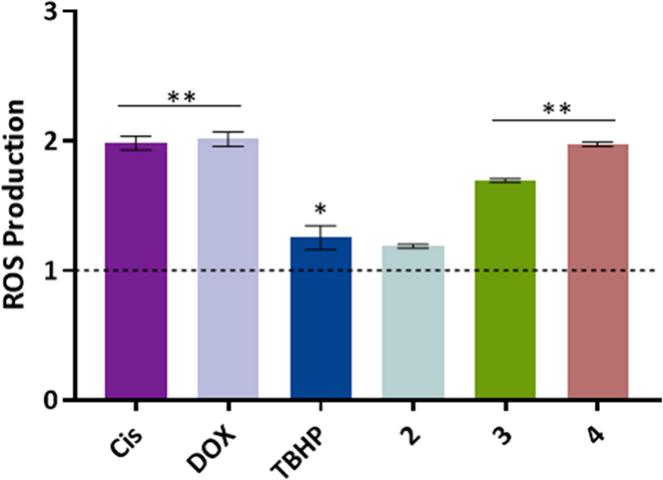
Evaluation of the production of reactive oxygen species (ROS) in A2780 cell line after 48 h of exposure to the IC_50_ of the complexes **2–4** by flow cytometry. DMSO 0.1% (v/v) was used as negative control (solvent control) and DOX 0.4 μM, cisplatin (Cis) 5 μM, and TBHP 22.2 μM as positive controls. Data normalized against the DMSO control (value represented as a dot line at *y* = 1) and expressed as the mean ± SEM of two independent assays. The symbols ** and * represent *p* < 0.0005 and *p* < 0.05, respectively. Statistical analysis was performed by two-way ANOVA method.

The increase of intracellular ROS has been correlated with programmed cell death.^82^ The electron transport chain present in the membrane of the mitochondria is one of the main sources of ROS production.^83^ An excessive production of ROS by the mitochondria can lead to the occurrence of autophagy, damage in the DNA, apoptosis, and oxidation of amino acids in proteins.^83^ The complexes by itself can also produce ROS and trigger cell death.^4^ Therefore, the production of ROS was investigated through the exposure of the A2780 cell line to IC_50_ concentrations of complexes **2–4** (Figure 13). Complexes **3** and **4** exhibits higher levels of ROS production 1.7× and 1.9× higher than the control (DMSO), respectively, and comparable to the levels induced by the positive controls DOX and cisplatin (Figure 13). Even though complex **2** induces only a slight increase in the % of ROS (Figure 13), these levels are enough to trigger autophagic cell death (Figure 13) and loss of cell viability (Figure 6). These results, particularly for complexes **3** and **4**, indicate that they are capable of inducing ROS production that in turn triggers apoptosis and autophagy leading to the loss of A2780 cell viability (Figures 8–12). The observed increase in ROS might be correlated with the levels of cytotoxicity, which is in agreement with several reports supporting that cobalt complexes are able to provoke DNA damage and increase ROS production, resulting in cell death.^84−86^ This ability could be related to the oxidation and reduction processes that may occur for cobalt complexes in a hypoxic environment,^84^ since the Co(III) reduction to Co(II) typically occurs between 0.4 and −1.28 V for this type of complexes,^45,65^ reduction potential of the cytoplasmic environment of the cell is −0.298 V.^87^

### Cell Cycle Progression

The cell death provoked by the presence of different metal complexes may also be associated with their cytostatic properties.^88,89^ The metal complexes can damage the DNA, leading to the trigger of cell cycle checkpoints and consequently to the arrest of the cell cycle. If the damage is not repaired, cell death can occur.^88,89^

**Figure 14:**
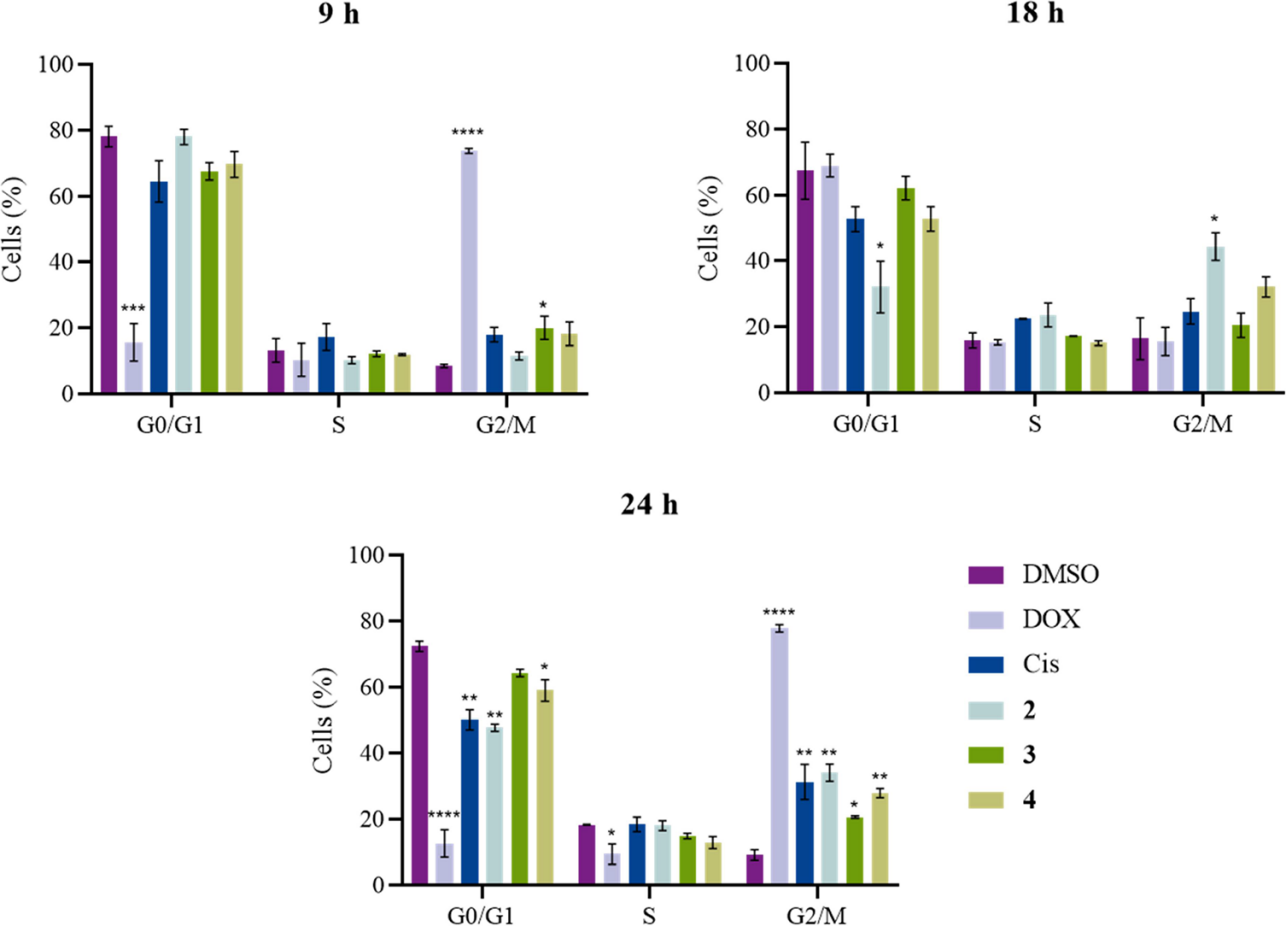
Cell cycle progression in A2780 cells after 9 h, 18 h, and 24 h of exposure to the IC_50_ concentrations of the complexes **2–4**, 0.4 μM DOX, 5 μM Cisplatin or DMSO 0.1% (v/v). DMSO 0.1% (v/v) was used as a vehicle control. Data expressed as the mean ± SEM of two independent assays. The statistical analysis was done by the two-way ANOVA test against the DMSO control. The symbols *, **, *** and **** represent *p* < 0.1, *p* < 0.01, *p* < 0.001 and *p* < 0.0001, respectively.

As a way to evaluate the cytostatic effect of the complexes **2–4**, the DNA content in each phase of the cell cycle (G0/G1, S, and G2/M) was evaluated by flow cytometry, by using propidium iodide (PI), a fluorescent marker that can intercalate with DNA, and a thymidine solution to block the cells in the early S phase of the cell cycle.^90^ This study was performed at 9, 18, and 24 h after the exposure to the IC_50_ concentrations of the complexes **2–4** (Figure 14).

The results show that all the complexes have a cytostatic effect in the A2780 cells after 24 h, which is confirmed by comparing the % of cells present in the G2/M phase in the DMSO control (9.2%) and in the presence of the complexes (34.1% for complex **2**, 20.7% for complex **3,** and 28% for complex **4**) or positive controls, DOX, and cisplatin (77.9 and 31.3%, respectively). These results are in line with the literature, where it is described that DOX, cisplatin, and the copper complexes have, in fact, a cytostatic effect associated.^91−94^ They are also in line with the previous results from Table 3, where it is shown that the complexes tend to accumulate in the nucleus of the cells.

### Evaluation of the Interaction with pDNA

Considering that all complexes **2**–**4** were able to enter the nuclei (Table 3), the evaluation of the interaction of the complexes with the DNA of the cells (in the case of this study A2780 cells) is really important since most of the chemotherapeutic complexes have as target the DNA.^95^ This way, 100 ng of plasmidic DNA (pUC18) were incubated with different concentrations (5, 25, 50, and 100 μM) of the complexes **2–4** for 24 h. pUC18 incubated with 5 mM Tris–HCl, 50 mM NaCl (pH 7.02), pUC18 incubated with DMSO, and pUC18 incubated with *Hind III* endonuclease for 2 h were used as controls (Figure S12). The normal conformation of the pDNA is the supercoiled isoform and when both of the chains are cleaved the pDNA adopts a linear isoform. The Hind III is a restriction endonuclease, which can cleave the double chain DNA and has a recognition sequence of 5′ A-AGCTT, also present in pUC18. When pUC18 is exposed to the activity of Hind III, it suffers hydrolyses on its chains and transforms to its linear isoform. Therefore, pUC18 exposed to *Hind III* will be the control for the detection of this isoform.

In Figure S12, it is possible to observe three distinguish bands, the nicked isoform (N), the linear isoform (L), and the supercoiled isoform (SC). The control with *Hind III* presents only the linear isoform, while the pUC18 and DMSO controls present the supercoiled isoform. On the other hand, for all the complexes **2–4,** it is possible to observe the two isoforms mentioned above and a third one corresponding to the nicked isoform, which will increase in intensity with the increase of the concentration of the complexes. Therefore, it is possible to say that complexes **2–4** can interact with the pDNA and induce its cleavage, the concentration of 25 μM being the one where the saturation of this interaction is observed (Figure S12). These results agree with the literature.^4^

### Determination of the DNA Cleavage Mechanism

Once it has been shown that the complexes are able to cleave the pDNA it is important to understand the mechanism through which this happens. For that, it was used as a reactive oxygen species scavenger, NaN_3_, which is an oxygen singlet scavenger. Therefore, pUC18 was incubated with a combination of 25 μM of each complex and 50 μM of NaN_3_ (Figure S13).

As shown in Figure S13, it is possible to identify the three isoforms of the pDNA (nicked, linear, and supercoiled) for all the combinations tested. It is also shown that in the presence of NaN_3_ alone, the majority of the pDNA is in its supercoiled isoform and the same is observed with the combination of the NaN_3_ and complexes **2–4**. The results for NaN_3_ agree with the literature since this agent scavenges singlet oxygen-free radicals that might be formed. When complexes are incubated in the presence of NaN_3_ (singlet oxygen scavenger), a disappearance of the circular isoform is observed with a pattern like pDNA control (Figure S13, only the supercoiled isoform (SC) is observed), which may indicate that the complexes can trigger oxidative stress via singlet oxygen^96^ and as previously observed in Figure 13. On the other end, when complexes are incubated with catalase, a hydrogen peroxide scavenging, the circular isoform is still observed as in the presence of complexes alone (Figure S14). When we increase complex **2** concentration in the presence of catalase, there is an appearance of the linear isoform (double-strand break) (Figure S14).

As NaN_3_ results demonstrated that the Co(II) complexes may cleave pDNA via singlet oxygen, incubation of complexes in the presence of D_2_O (which prolongs the half-life of singlet oxygen) was also evaluated (Figure S15). As observed in Figure S15, it seems that incubation of D_2_O induces a high level of circular isoform at least for complex **3**. In the case of **2,** no increase in the circular isoform might be observed in the presence of D_2_O, which could be associated with a saturation of the cleavage for this time point and concentration of complex **2**. This observation, together with the fact that NaN_3_ can reverse the pDNA cleavage, may provide evidence that complexes **2** and **3** cleave pDNA through singlet oxygen.^96,97^

### Single-Cell Gel Electrophoresis Assay (Comet Assay)

To further examine the DNA damage induced by complexes **2–4** in in vitro A2780 cells, the comet assay was performed. This assay is recognized as an effective method to evaluate the DNA integrity when exposed to different damage agents.^98,99^Figure S16 shows a representation of the DNA damage (“comets”) of A2780 cells in the presence of 0.1% DMSO, 0.05% H_2_O_2_ (positive control), and 10× IC_50_ concentrations of complexes **2–4**.

As observed in Figure S16–C, A2780 cells exposed to DMSO show a 12.9% of DNA in the tail, while exposure of cell to hydrogen peroxide leads to 73% of DNA in the tail. When it comes to our complexes **2**, **3,** and **4**, a 3.1×-fold (39.7%), 3.2×-fold (42.4%), and a 4.4×-fold (56.4%) increase in DNA in the tail (DNA damaged) compared to DMSO control, respectively. These results, together with ROS generation (Figure 13), indicate that Co(II) complexes when exposed to A2780 cells can induce ROS that trigger genomic DNA fragmentation.

### *Calf Thymus* DNA (CT-DNA) Binding Assays

To further understand the binding of complex **2**, **3,** and **4** to DNA, incubation of complexes **2**–**4** in the presence of different concentrations of *calf thymus* DNA (CT-DNA) was evaluated by UV-spectroscopy (Figure S17).

Figure S17 demonstrates that complexes **2–4** interact with CT-DNA and by increasing its concentration an effect in the lowest-energy absorption band is observed, namely, a small hyperchromic effect for complex **2** and a hypochromic effect for **3** and **4,** with no major shifts on the bands. The hyperchromic effect is indicative of different binding modes to CT-DNA which may be due to the unwinding of DNA strands or damaging the DNA double helix, minor groove binding, or an external interaction such as electrostatic binding, while the hypochromic effect is usually associated with the strong staking interactions between the aromatic parts of the complexes and DNA. The π* orbital of the complexes couples with the π orbital of base pairs of CT-DNA, and the conformational change in CT-DNA leads to a decrease in the absorbance.^100,101^ The intrinsic binding constants *K*_b_ were calculated via the Wolfe–Shimmer equation and are 0.2 × 10^4^ M^–1^ for **2**, 1.6 × 10^4^ M^–1^ for **3,** and 0.9 × 10^4^ M^–1^ for **4**, values that are lower than what is described for other cobalt complexes and also doxorubicin.^99,100^ To complement these results, the melting profile, using Evagreen dye, of 10 μM CT-DNA in the presence and absence of 10 μM of complexes **2–4** or 0.1% of DMSO was performed (Figure S18). Through the melting profile analysis (Figure S18), it is possible to verify that the addition of Evagreen dye to CT-DNA that has been incubated (with or with 0.1% DMSO) but in the absence of complexes **2**-**4**, allows to obtain a typical melting profile for CT-DNA. On the other hand, a prior incubation of CT-DNA in the presence of complexes **2**-**4** for 1h30 prior to adding Evagreen dye, prevented the acquisition of Evagreen fluorescence (associated with its binding to DNA). Taken together, our data suggests that our cobalt complexes can interact with DNA possibly by a groove binding mechanism (Figure S18) but particularly, can induce DNA damage (cleavage) (Figures S12–S16).

### Glutathione (GSH) Interaction Assay

Glutathione (GSH) is an important enzyme involved in the detoxification process of free radicals and ROS as well as DNA biosynthesis, protection of cells against various oxidative stresses, intracellular signal transduction, and gene regulation, and can also induce complex reduction.^102^ To study the effect of incubation of glutathione with our complexes **2–4** in a biological medium, UV–vis spectra of the complexes in the presence and absence of GSH after 24 h incubation at 37 °C were obtained (Figure S19). The UV–vis spectra (Figure S19) show that there are no major changes in complex **2–4** typical bands profile in the absence or presence of GSH, indicating that no ligand displacement has occurred or new bands due to the GSH-complex formation. Nevertheless, the slight increase in absorbance is indicative of a change in the complex environment. Nevertheless, we cannot exclude the hypothesis that Co(III) can be reduced to Co(II) or even to Co(I) even before internalization within cells (e.g., what happens of Fe(III) to Fe(II) and Cu(II) to Cu(I) which can then be internalized by the respective plasma membrane transporters.^76^ Only providing a detailed cell analysis of the route of internalization of Co(III) complexes could give us valuable information to understand tumor cells' response to our complexes.

### Cell Migration Assay

**Figure 15:**
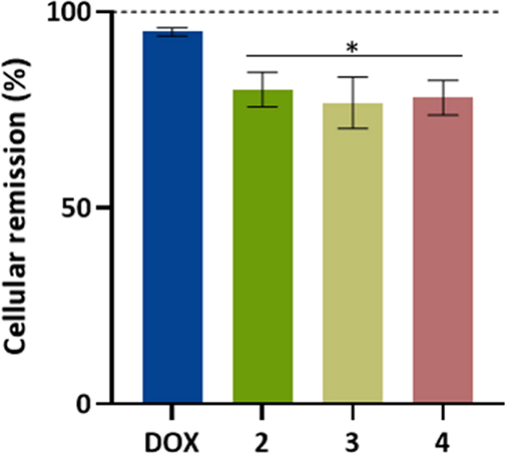
Evaluation of the fibroblasts’ remission after 24 h of exposure to the IC_50_ of the complexes **2–4** by inverted microscopy. DMSO 0.1% (v/v) was used as negative control (vehicle control) and DOX 0.4 μM was used as positive control. Data normalized against the DMSO control (value represented as a dot line at *y* = 100%) and expressed as the mean ± SEM of three independent assays. The symbols * represent *p* < 0.05.

Some cobalt complexes can also present an antimetastatic behavior,^103^ and to evaluate this, a cell migration assay was used. In this assay, fibroblasts are grown in a monolayer, scratched, and then exposed for 24 h to the IC_50_ concentrations of complexes **2–4**. The percentage of cellular remission (at the scratch) is compared to cells exposed to only vehicle control (DMSO) (Figures 15 and S20).

Figure 15 clearly shows significantly lower cellular remission in the presence of complexes **2** and **4** (80.2 and 78.1%, respectively). Complex **3** also shows lower cellular remission (76.9%) when compared to the control. These results indicate that the tested complexes, especially complexes **2** and **4**, have antimetastatic properties, which might be considered a good indicator for a potential therapeutic agent avoiding the spread of the tumor.

### Ex Ovo Chick Embryo Yolk Sac Membrane (YSM) In Vivo Assay

**Figure 16:**
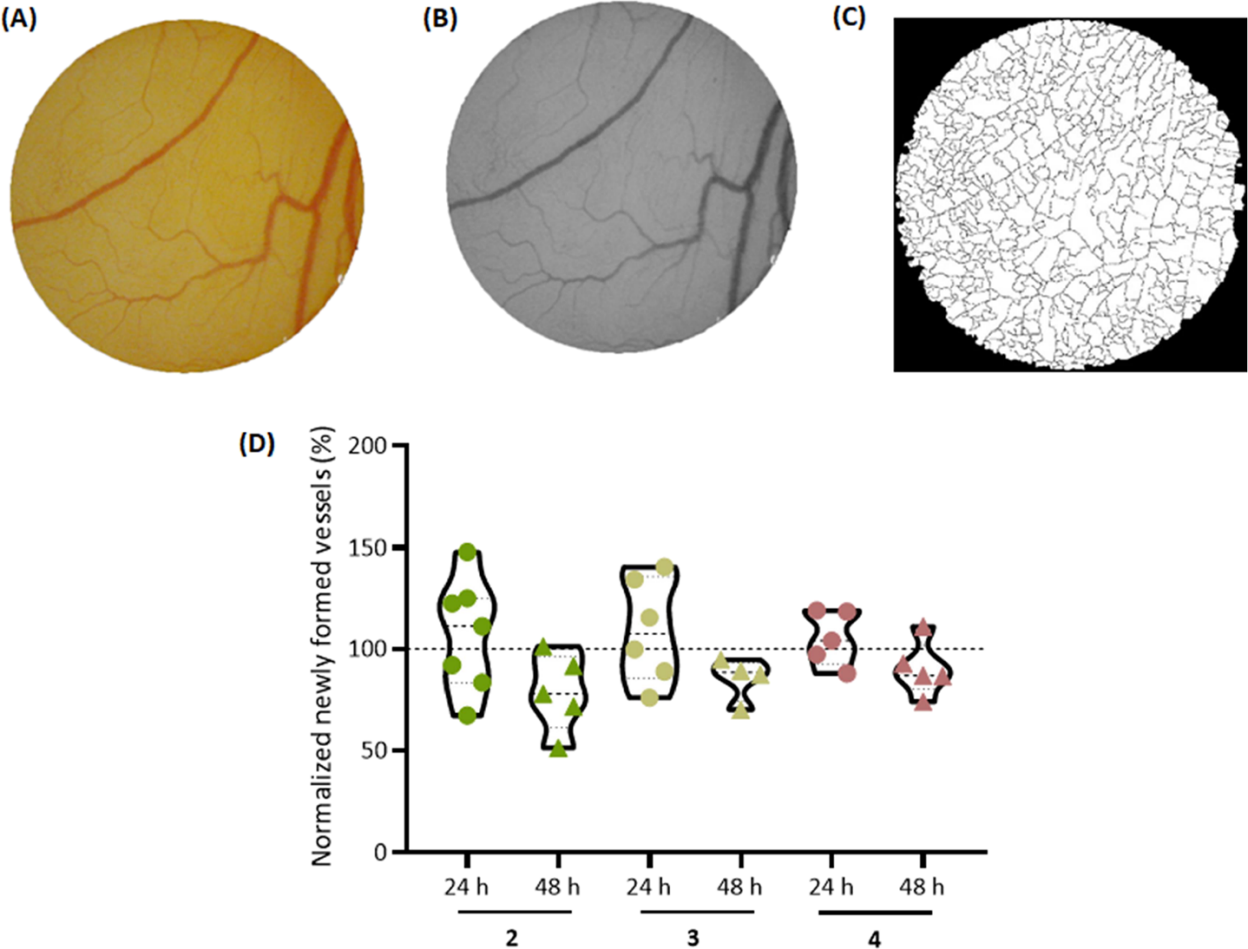
Evaluation of the complexes potentiality to modulate angiogenesis. (A) RGB image of the O-ring interior; (B) Green channel of the same image used for counting the number of veins; (C) Binary of the segmented image used to calculate the number of branches; (D) Percentage of newly formed vessels in ex ovo YSMs after 24 and 48 h exposure to IC_50_ concentrations of complexes **2–4**. It was used at least 7 independent chicken embryos experiments for each condition. Data normalized against the DMSO control (100% dotted line) and expressed as the mean ± SEM of two independent assays.

Ex ovo chick embryo yolk sac membrane (YSM) assay is a simple in vivo model to evaluate the cytotoxicity of different complexes and their potentiality to modulate angiogenesis, being pro-angiogenic or antiangiogenic.^104,105^ For this purpose, YSMs of chicken embryos were challenged with the IC_50_ concentrations of complexes **2–4** for 48 h. All newly formed vessels were counted at different time points after exposure (0 h (control), 24 h, and 48 h) and compared with the formed vessels for vehicle controls (DMSO 0.1%, Figure 16). In what concerns the formation of new vessels, complexes **2–4** do not seem capable of inducing an angiogenic process, which is a promising issue. In fact, after exposure to complexes **2–4**, the number of newly formed vessels seems to decrease from 24 to 48 h.

Additionally, this assay also permits the study of the toxicity in vivo^106^ of the complexes by analyzing the death of the embryo during that period of incubation. When this happens, it might indicate that complexes are highly toxic to the embryo. In our case, no in vivo embryo toxicity was observed when the chicken embryos were exposed to the IC_50_ of the different cobalt complexes for 48 h, which is an important indicator for in vivo absence of toxic side effects in nontumor cells.

## Conclusions

A new family of half-sandwich Co(III)-cyclopentadienyl complexes of general formula [Co(η^5^-C_5_H_5_)(PPh_3_)(NN)][(CF_3_SO_3_)_2_], where NN represents the *N*,*N*-bidentate coligands was synthesized and fully characterized. The coordination geometry around the Co(III) center is described, by DFT methods, as a typical piano-stool structure. The main structural feature of this new family of compounds was found on a metal-to-ligand charge transfer band from the cobalt center to the η^5^-Cp ligand, d_Co_ to π*_Cp_, in which inherent electronic delocalization arising from the NN chosen coligand after its coordination to the CoCp moiety seems to be correlated with the enhanced cytotoxicity and selectivity of the complex.

From all the cancer cell lines tested, A2780, HCT116, MDA-MB-231, and MCF-7, the Co(III) complexes showed a high antiproliferative activity in A2780 and HCT116 cancer cell lines. Interestingly, the Co(III) complexes are not cytotoxic for none of the breast cancer cells studied (MDA-MB-231 and MCF-7), except complex **4**, which is moderately cytotoxic for MCF-7 cells (IC_50_ of 10.2 μM). Also, the complexes show some intrinsic selectivity toward cancer cells compared to normal fibroblast cells. Co(III) complexes were able to internalize A2780 cancer cells by passive diffusion and also via an active mechanism. Once inside cells, complexes can accumulate in the mitochondria and are able to trigger ROS production which was also proved to occur by an in vitro exposure of our complexes to pDNA. Indeed, incubation of complexes with pDNA leads to DNA damage (single-strand cleavage) due to an oxidative mechanism that is clearly dependent on single oxygen, particularly for complex **3**. These results, together with their nuclear accumulation, trigger DNA fragmentation (damage) as shown by the comet assay, which translates to A2780 cell cycle delay, leading at the end to the observed cell death via apoptosis and autophagy, Indeed, in vitro incubation of Co(III) complexes with different CT-DNA concentrations lead to a small hyperchromic effect for **2** and a hypochromic effect for **3** and **4**, indicative of DNA damage and groove binding interactions with *K*_b_ values lower than other Co(II) complexes in the literature. Moreover, melting curve analysis of CT-DNA in the presence of complexes led to total quenching of Evagreen dye fluorescence which further validated their interaction with DNA. Our results point out the best performance for complex **4** toward A2780 cancer cells, whose IC_50_ value is quite close to the cisplatin (4.7 ± 0.09 vs 1.90 ± 0.20 μM) and has also an excellent selective index (SI = 21.3).

Based on previous data on metal complexes internalization several hypotheses can be considered. Co(III) can be reduced to Co(II)/Co(I) even before internalization within cells (e.g., what happens of Fe(III) to Fe(II) and Cu(II) to Cu(I)^76^) which can then be internalized by the respective plasma membrane transporters. However, we cannot exclude the fact that our Co(III) complexes could be also internalized by endocytosis via transferrin (such as some Ru(III) complexes) or albumin receptors. Future studies using inhibitors for each specific human cell-based transporter will provide a very nice and detailed study for a subsequent paper, namely addressing the internalization of complexes **2**-**4** in cells as well as additional in vivo targets using proteomic studies. It is also worth mentioning that complexes **2**-**4** show to be antimetastatic in vitro and nontoxic in vivo in an ex ovo YSM model which is also a good indication for further in vivo studies.

A tentative to find a structure/activity relationship suggests that the structures enhancing π back-donation, d_Co_ to π*Cp, are experimentally related to blue-shifted MLCT bands observed in the UV–vis spectra, will favor the increase of cytotoxicity as well as the selectivity for cancer over noncancer cells. Therefore, this consideration must be taken in account when further designing new CoCp-based complexes as anticancer agents. The results obtained so far pave the way to further explore this emerging family of Co(III)Cp as potential metallodrugs that can constitute a good alternative to drugs based on more expensive metals such as, e.g., platinum, gold, and ruthenium.

## Supporting Information Available

Compound stability analysis by NMR spectroscopy; DFT calculations and antiproliferative activity; and NMR, FTIR, and UV–vis spectra (PDF)

## Abbreviations

bipy: 2,2′-bipyridine
phen: 1,10′-phenanthroline
Me2bipy: 4,4′-dimethyl-2,2′-bipyridine
NH2phen: 5-amino-1,10′-phenanthroline
