# Supplementary material for: Exploiting Co(III)-Cyclopentadienyl Complexes To Develop Anticancer Agents
Source: Inorg Chem. 2024 Mar 19;63(13):5783–804. doi: 10.1021/acs.inorgchem.3c03696 (PMC10988555; doi:10.1021/acs.inorgchem.3c03696)
Supplement: Supplementary file 1 — ic3c03696_si_001.pdf [file ic3c03696_si_001.pdf]

Supporting Information

Exploiting Co(III)-Cyclopentadienyl Complexes to Develop Anticancer Agents

João Franco Machado[a,&], Sandra Cordeiro[b,c&], Joana N. Duarte[a], Paulo J. Costa[d], Paulo J. Mendes[e], Maria Helena Garcia[a,*], Pedro V. Baptista[b,c,*], Alexandra R. Fernandes[b,c,ф,*], Tânia S. Morais[a,ф,*]

[a] Centro de Química Estrutural, Institute of Molecular Sciences, Faculdade de Ciências, Universidade de Lisboa, Campo Grande, 1749-016 Lisboa, Portugal

[b] Associate Laboratory i4HB - Institute for Health and Bioeconomy, NOVA School of Science and Technology, NOVA University Lisbon, 2819-516 Caparica, Portugal

[c] UCIBIO, Departamento de Ciências da Vida, Faculdade de Ciências e Tecnologia, Universidade Nova de Lisboa, 2819-516 Caparica, Portugal.

[d] BioISI – Biosystems & Integrative Sciences Institute, Faculty of Sciences, University of Lisboa, Campo Grande, 1749-016 Lisboa, Portugal

[e] LAQV-REQUIMTE (Polo de Évora), Escola de Ciências e Tecnologia, Universidade de Évora, R. Romão Ramalho 59, 7000-671 Évora, Portugal

[&]Both authors contributed equally to the work.

[ф]Co-last authors

* Co-corresponding authors

Table of contents

Synthesis and characterization of new CoCp complexes

DFT calculations

Antiproliferative Activity

Supplementary NMR Data

Supplementary FTIR Data

Supplementary UV-Vis. Data

Synthesis and characterization of new CoCp complexes

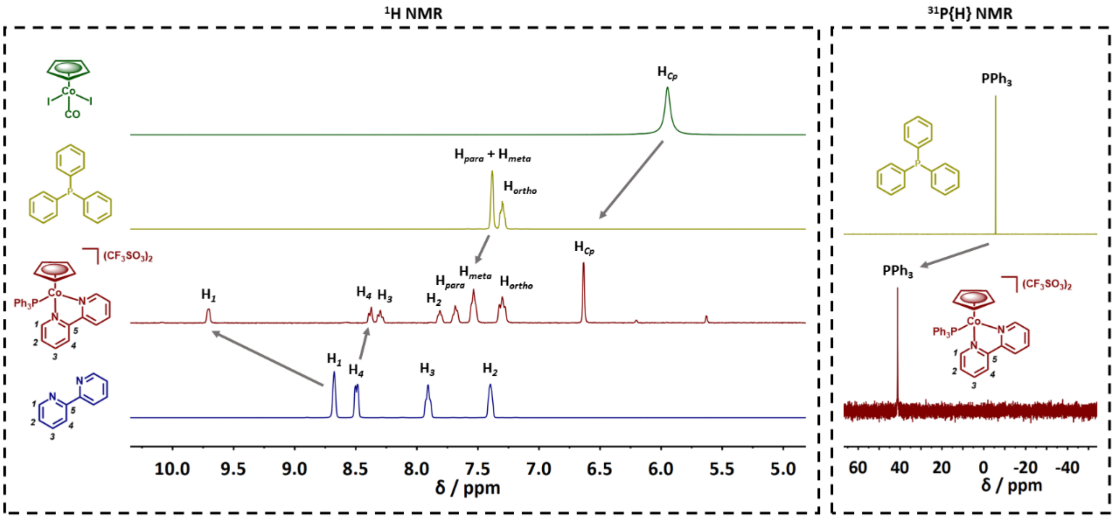

Figure S1. ^{1}H NMR (left) and $^{31}P\{H\}$ NMR (right) spectra of $[CoCp(PPh_3)(bipy)][(CF_3SO_3)_2]$ (**1**, red), $[CoCp(CO)I_2]$ (dark green), free PPh_3 (light green) and free bipy (blue) in acetone-d^{6} at room temperature.

Table S1. Electronic absorption spectra of complexes $[CoCp(PPh_3)(NN)][(CF_3SO_3)_2]$ (**1** – **4**) the precursor complex $[CoCp(CO)I_2]$, and free ligands in dichloromethane solutions.

Complex	λ_{max} / nm (ε / $M^{-1}.cm^{-1}$)	NN free ligand	λ_{max} / nm (ε / $M^{-1}.cm^{-1}$)
**1**	236 (3.31×10^{4})<br>241 (3.04×10^{4})<br>255 (3.19×10^{4})<br>305 (3.00×10^{4})<br>315 (sh)<br>355 (sh)<br>464 (3.90×10^{3})	bipy	238 (1.78×10^{4})<br>245 (sh)<br>283 (2.24×10^{4})
**2**	259 (3.27×10^{4})<br>305 (sh)<br>312 (2.73×10^{4})<br>340 (sh)<br>464 (2.83×10^{3})	Me_2bipy	243 (1.18×10^{4})<br>248 (1.09×10^{4})<br>282 (1.55×10^{4})
**3**	277 (2.97×10^{4})<br>345 (sh)<br>370 (sh)<br>467 (2.69×10^{3})	phen	265 (4.56×10^{4})
**4**	239 (9.61×10^{3})<br>259 (1.01×10^{4})<br>290 (8.75×10^{3})<br>339 (3.15×10^{3})	NH_2phen	253 (8.67×10^{3})<br>279 (1.29×10^{4})<br>327 (3.61×10^{3})
$[CoCp(CO)I_2]$	256 (1.76×10^{4})<br>280 (1.77×10^{4})<br>375 (sh)<br>537 (1.59×10^{3})		

DFT calculations

Table S2. Relevant TD-DFT excitation energies (λ), oscillator strengths (f) and compositions (only those > 5% are shown), for complexes **1**-**4**. These correspond to the excitations highlighted with an asterisk in Figure 5. The calculated values are compared with experimental data (λ_{exp}). Both calculated and experimental values were obtained in dichloromethane.

Complex	λ (nm)	f	Composition	λ_{exp} (nm)
**1**	452	0.03820	H-8 → L (23.2%), H → L (9.6%), H-7 → L+2 (9.1%), H-11 → L (6.8%)	464
	319	0.10980	H → L (69.5%), H-8 → L (7.2%), H → L+2 (6.3%)	305
	291	0.11850	H-4 → L+1 (50.3%), H-3 → L+1 (8.7%), H-1 → L+1 (6.8%), H-7 → L+1 (6.0%)	255
**2**	456	0.03560	H-8 → L (13.6%), H → L (11.6%), H-12 → L (11.0%), H-7 → L (10.1%), H-8 → L+2 (8.9%), H-7 → L+2 (7.1%), H-13 → L+2 (5.2%)	464
	316	0.15990	H → L (64.8%), H-8 → L (6.0%), H-7 → L (5.5%)	312
	287	0.11680	H-3 → L+1 (36.2%), H-1 → L+1 (20.7%), H-5 → L (9.4%), H-4 → L+1 (9.0%), H-3 → L+2 (7.7%)	259
**3**	452	0.03910	H-9 → L (20.7%), H → L (15.3%), H-12 → L (7.3%), H-8 → L+2 (7.3%), H-9 → L+2 (5.3%)	467
	328	0.05760	H → L+1 (62.3%), H → L (23.9%)	
	321	0.11070	H → L (42.4%), H → L+1 (18.9%), H-9 → L (9.7%), H-1 → L (5.0%)	277
**4’**	459	0.03510	H → L (29.8%), H-9 → L+2 (9.2%), H-9 → L (7.2%), H-8 → L+2 (6.6%), H-8 → L (6.6%), H-10 → L+2 (5.6%)	339
	436	0.02590	H → L+1 (49.5%), H → L (29.1%)	290
	361	0.13770	H → L+3 (94.0%)	259
	315	0.11580	H-1 → L (62.9%), H-1 → L+2 (8.0%)	239
**4’’**	459	0.03480	H → L 28.2%, H-8 → L+2 (15.3%), H-9 → L (12.2%), H-11 → L+2 (8.1%)	339
	436	0.02720	H → L+1 (53.2%), H → L (27.2%), H-9 → L (5.1%)	290
	362	0.13590	H → L+1 (53.2%), H → L (27.2%), H-9 → L (5.1%)	259
	316	0.11150	H-1 → L (61.7%), H-9 → L (9.5%), H-1 → L+2 (7.4%)	239

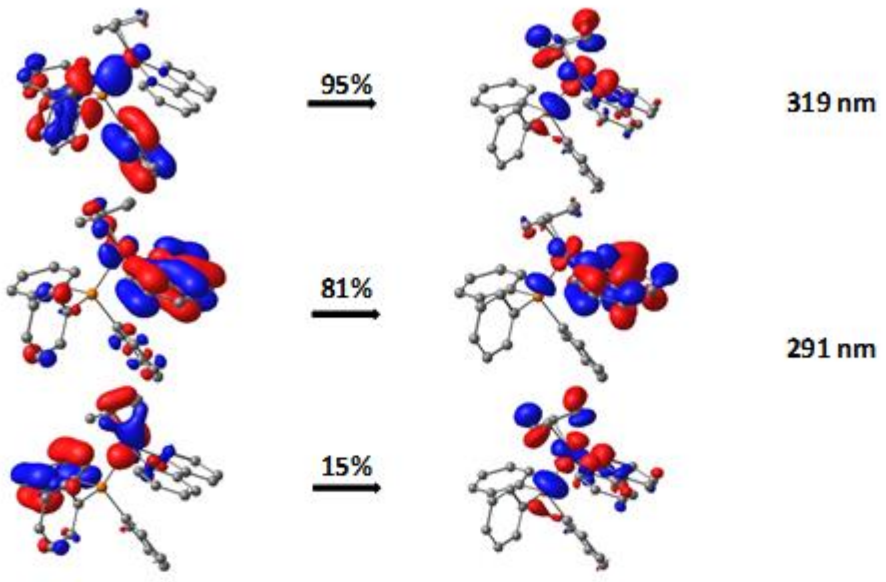

Figure S2. Representative natural transition orbital pairs along with the respective coefficients for the TD-DFT calculated high-energy excitations of complex **1**. More details are given in **Table S2**.

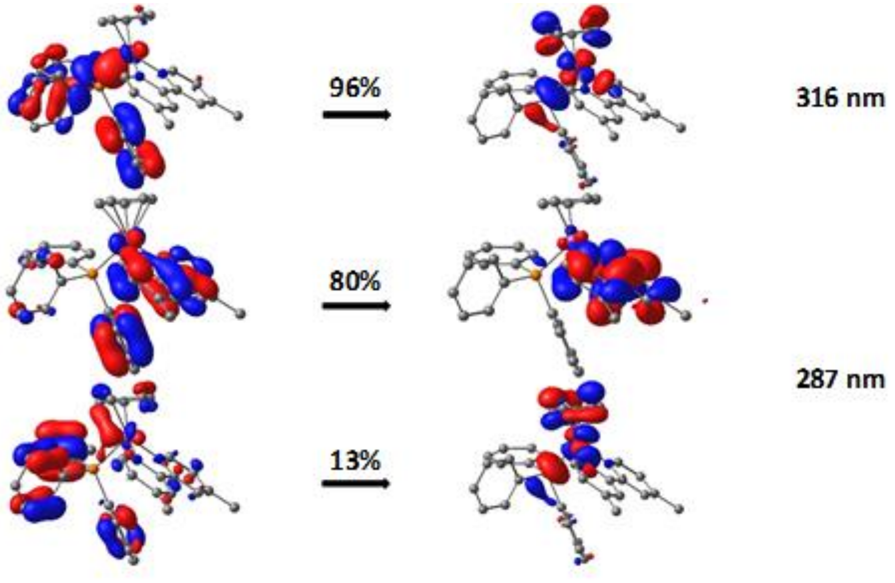

Figure S3. Representative natural transition orbital pairs along with the respective coefficients for the TD-DFT calculated high-energy excitations of complex **2**. More details are given in **Table S2**.

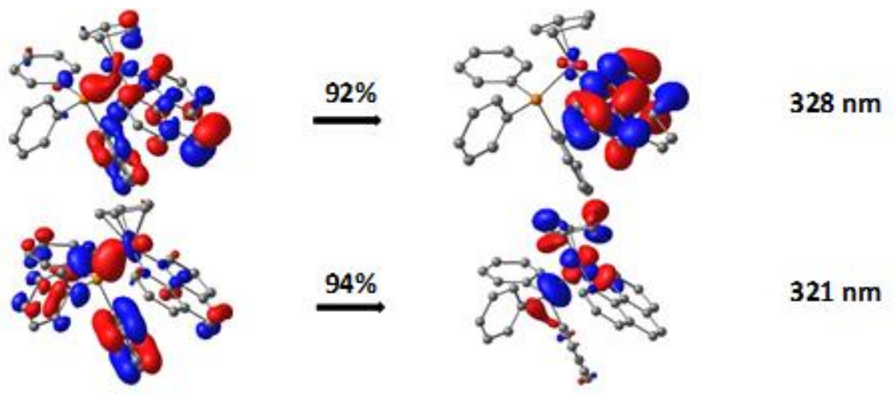

Figure S4. Representative natural transition orbital pairs along with the respective coefficients for the TD-DFT calculated high-energy excitations of complex **3**. More details are given in **Table S2**.

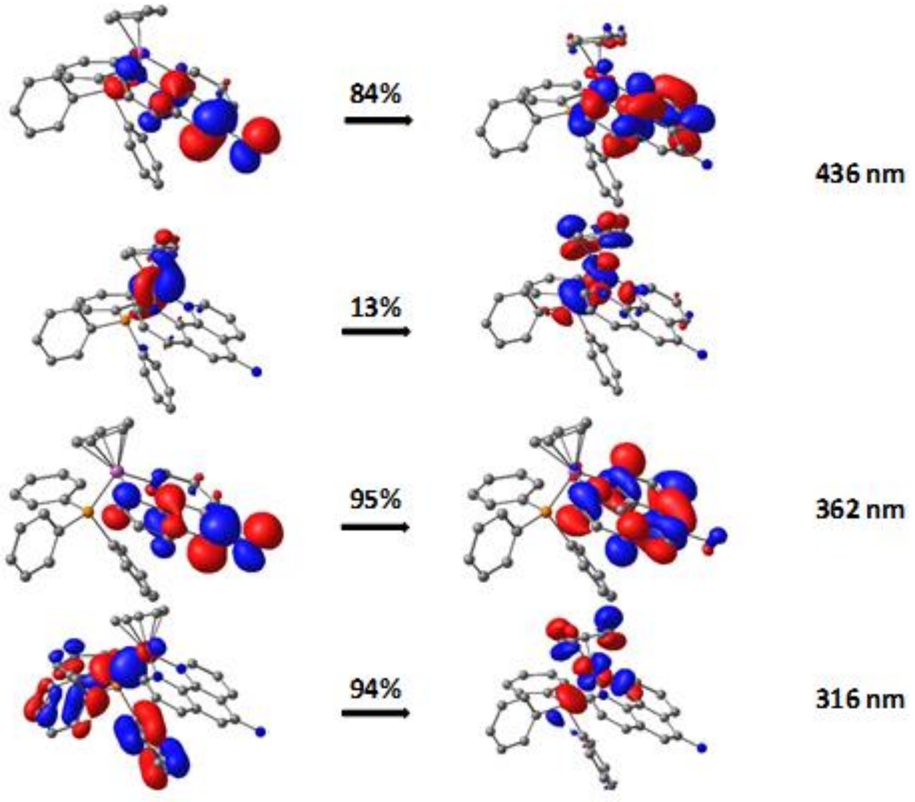

Figure S5. Representative natural transition orbital pairs along with the respective coefficients for the TD-DFT calculated high-energy excitations of complex **4**. More details are given in **Table S2**. For clarity, only **4''** is shown, however, the transitions for **4'** are very similar.

Antiproliferative Activity

The antiproliferative activity of the ligands was also evaluated through the exposure of the A2780 and HCT116 cancer cell lines and a healthy human fibroblast cell line to 0.1 - 50 µM of all

ligands (**Figure S6**) for 48 h, using the CellTiter 96®Aqueous Non-Radioactive Cell Proliferation Assay, as described in materials and methods section.

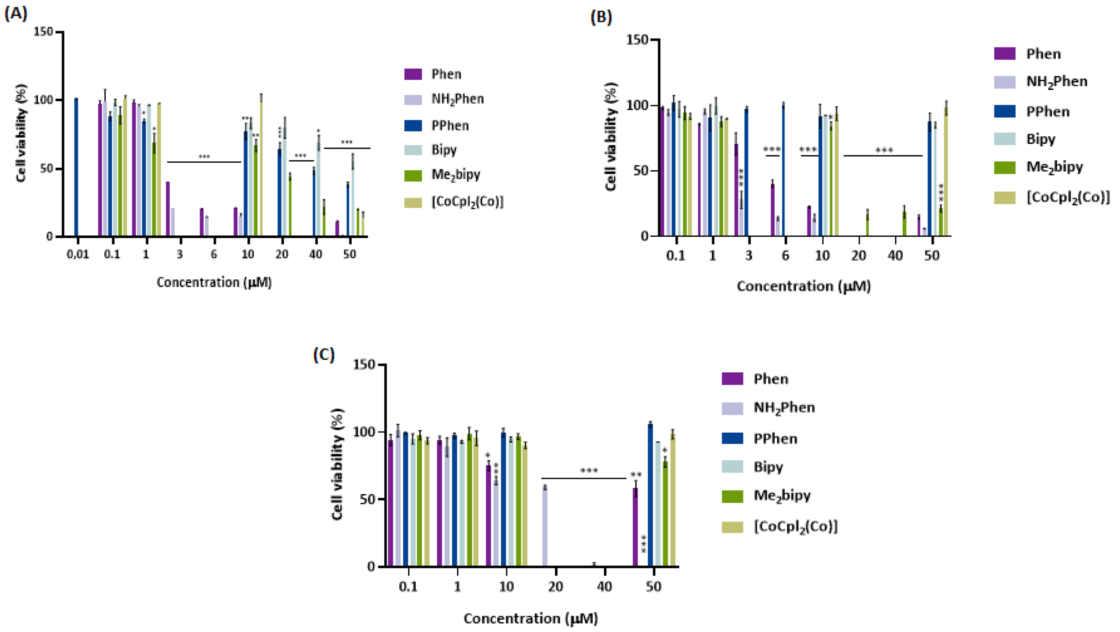

Figure S6. Cellular viability (%) in A2780 (A), HCT116 (B), and Fibroblasts (C) after 48 h of exposure to the different ligands. Data normalized against the control (0.1 % (v/v) DMSO) and expressed as the mean ± SEM of at least two independent assays. The symbols ***, ** and * represent $p < 0.0005$, $p < 0.005$ and $p < 0.05$, respectively.

The calculated IC_{50} and SI values of the ligands in the studied cell lines are shown in **Table S3**.

Table S3. Selectivity index (SI) and relative IC_{50} of the ligands in the A2780, HCT116, and Fibroblasts cell lines. Data expressed as mean ± SEM of at least two independent assays. * SI values calculated considering that the IC_{50} would be 50 µM (assuming the minimal value); - SI values not calculated.

Ligand	Cell lines	IC_{50} (µM)	SI
**Bipy**	A2780	> 50	-
	HCT116	> 50	-
	Fibroblasts	> 50	-
**Me_2bipy**	A2780	17.6 ± 0.1	2.8*
	HCT116	12.4 ± 0.2	4.0*
	Fibroblasts	> 50	-
**Phen**	A2780	2.4 ± 0.1	20.9
	HCT116	4.1 ± 0.1	12.4
	Fibroblasts	50.1± 0.1	-
**NH_2Phen**	A2780	1.9 ± 0.1	15.2
	HCT116	2.3 ± 0.1	13.1
	Fibroblasts	29.9 ± 0.4	-
**PPh_3**	A2780	37.8 ± 0.1	1.3*
	HCT116	> 50	-
	Fibroblasts	> 50	-
**[CoCp(CO)I_2]**	A2780	29.9 ± 0.1	1.7*
	HCT116	> 50	-
	Fibroblasts	> 50	-

To determine the IC_{50} of Cisplatin and doxorubicin, the CellTiter 96®Aqueous Non-Radioactive Cell Proliferation Assay using MTS was performed, as described in materials and methods (**Figure S**).

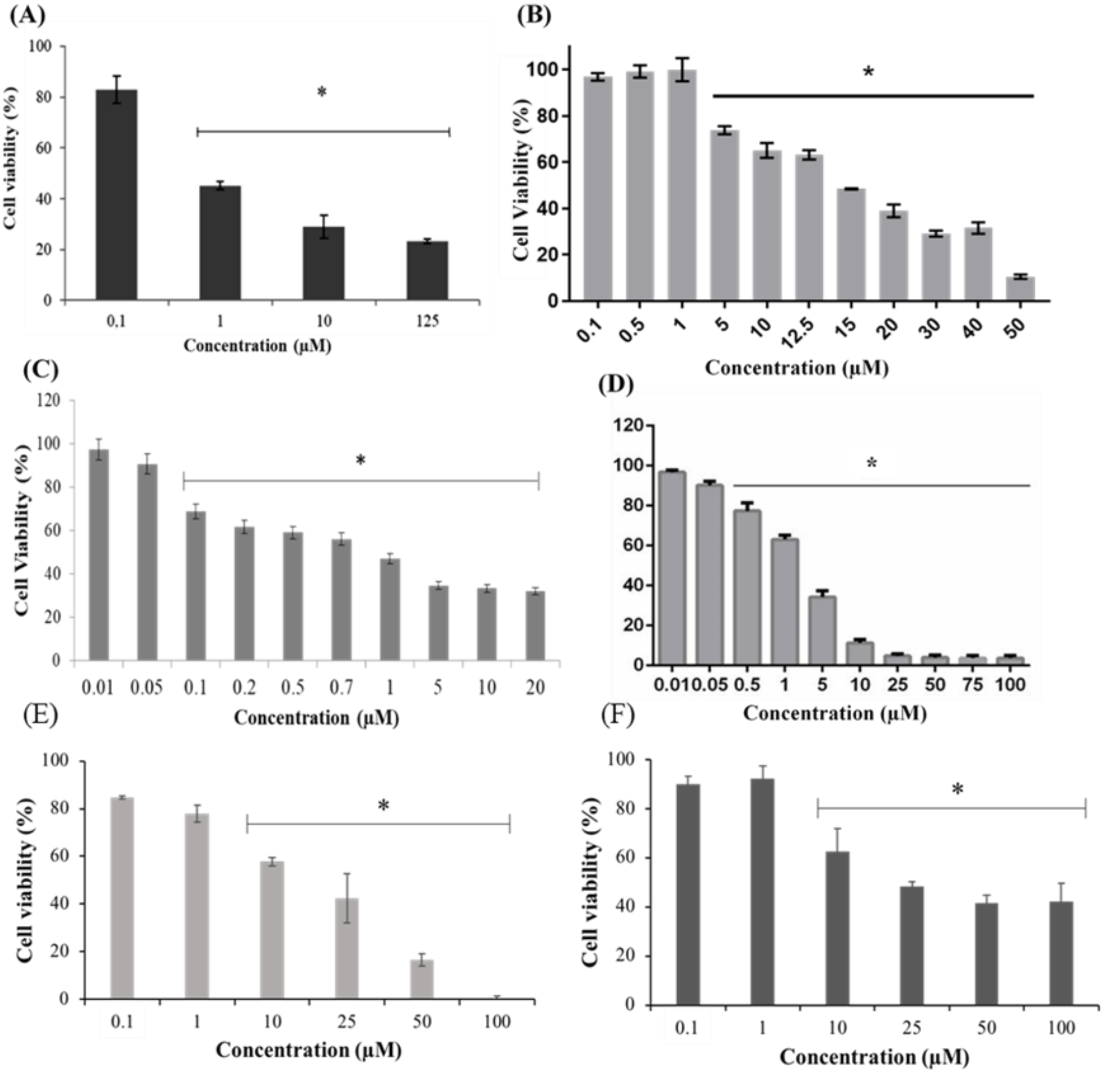

Figure S7. Viability of HCT116 cancer cell line after exposure to different concentrations of doxorubicin (A) and cisplatin (B) for 48 hours. Viability of A2780 cancer cell line after exposure to different concentrations of doxorubicin (C) and cisplatin (D) for 48 hours. Viability of Fibroblasts after exposure to different concentrations of doxorubicin (E) and cisplatin (F). 0.1% (v/v) DMSO was used as the vehicle control. Data are expressed as the mean ± SEM of three biological assays.

Evaluation of the antiproliferative activity of complexes **1 – 4** was also tested in a healthy human cell line (fibroblasts) in a range of concentration of 0.1 - 100 µM (**Figure S8**).

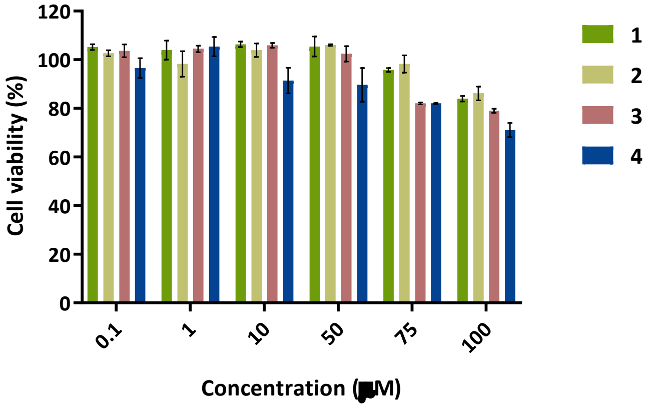

Figure S8. Cellular viability (%) of fibroblasts after 48 h of exposure to the complexes **1 – 4**. Data normalized against the control (0.1% (v/v) DMSO) and expressed as the mean ± SEM of at least two independent assays.

For the evaluation of the stability in a cell culture medium, complexes **1 – 3** were dissolved in RPMI medium without *phenol red* at concentrations of 50 µM and complex **4** at a concentration of 20 µM and incubated at 37°C at 0 h, 24 h, and 48 h (**Figure S9**).

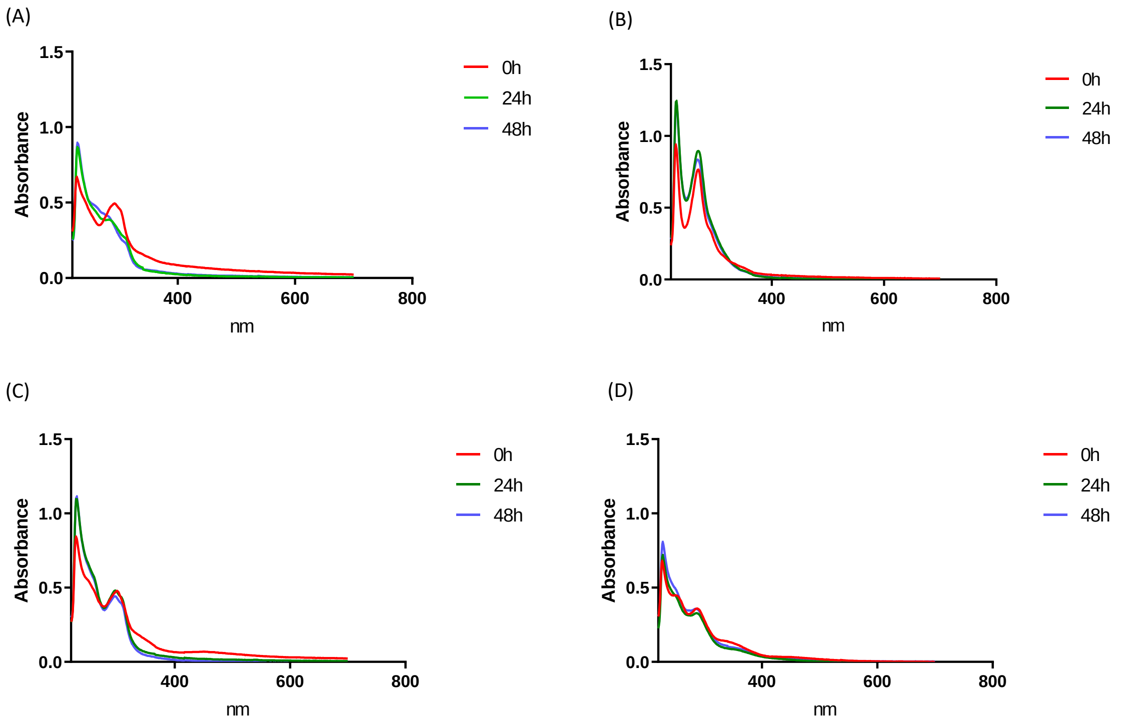

Figure S9. Stability evaluation of the cobalt complexes **1** (A), **2** (B), **3** (C), and **4** (D), at 50 µM for the complexes **1-3** and 20 µM for complex **4** for 0 h, 24 h, and 48 h at 37°C in biological medium.

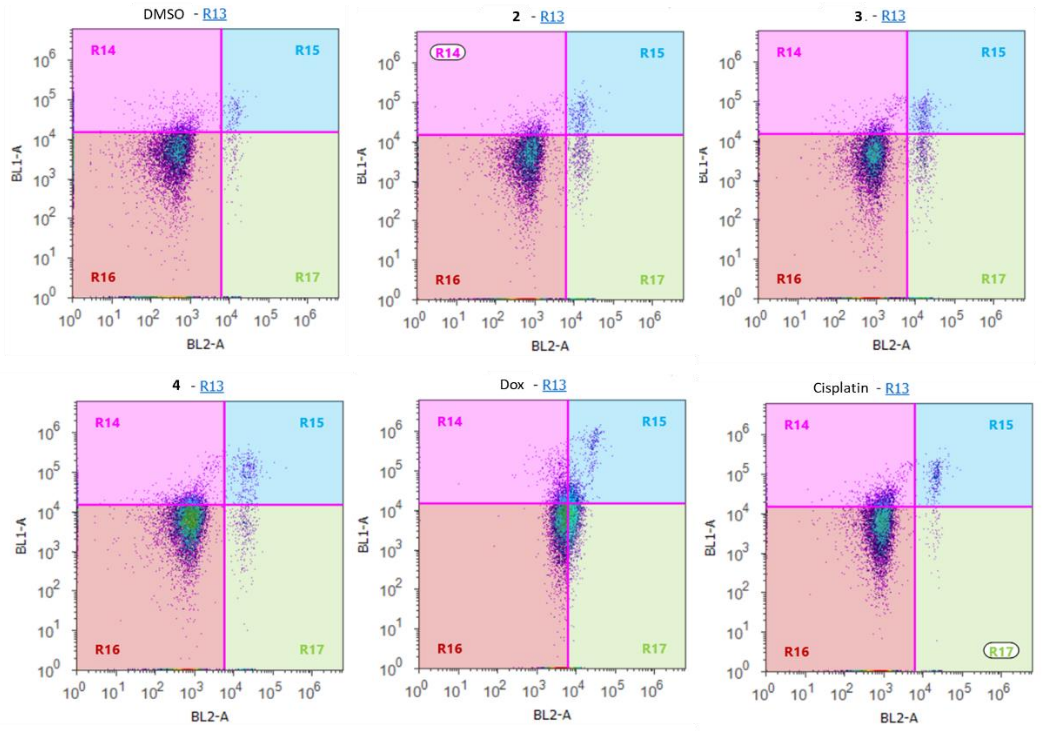

Figure S10. 2D FACS plots for the Apoptosis assay. R16 correspond to live cells, R14 cells in initial apoptosis, R15 cells in late apoptosis and R17 cells in necrosis.

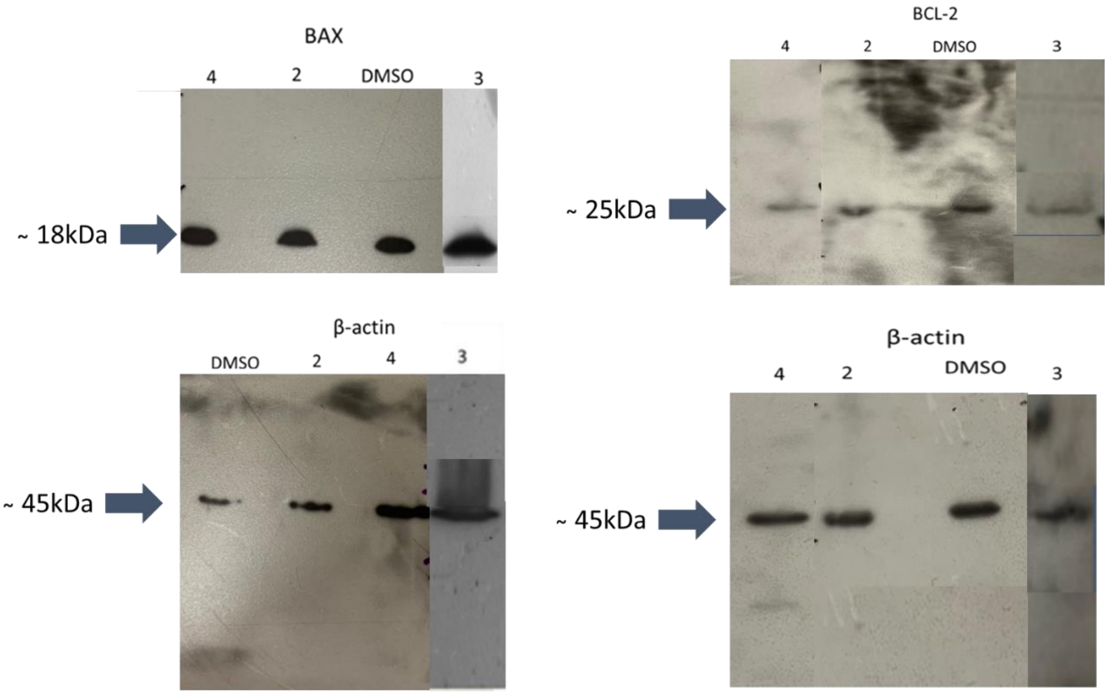

Figure S11. Western Blot bands used for the quantification of proteins BAX and BCL-2 in A2780 cells after their exposure to complexes **2 – 4**, or DMSO.

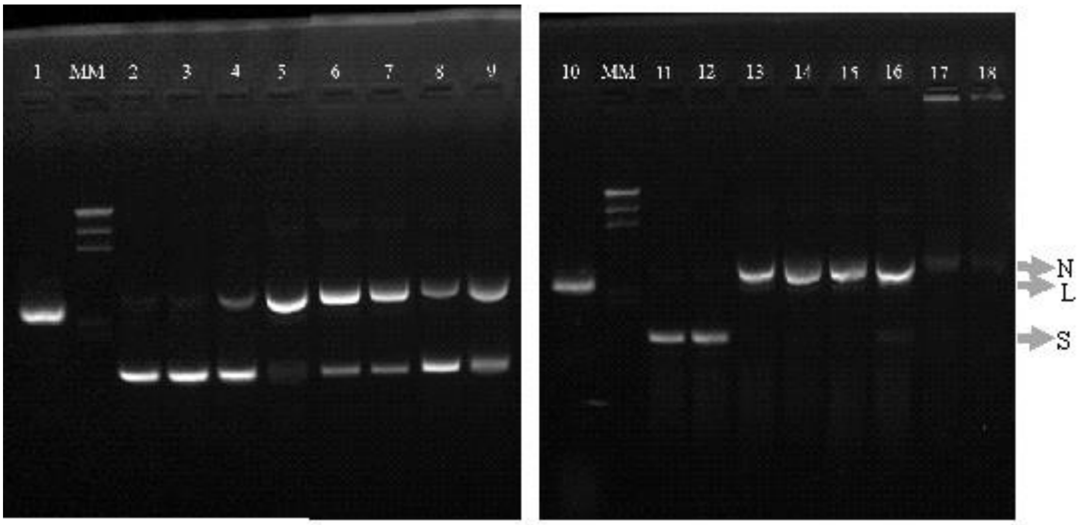

Figure S12. Electrophoresis in 0.8% (w/v) agarose gel in TAE buffer at 70 V constant voltage for 1h with the samples from the interaction of different concentrations (5, 25, 50 and 100 µM) of the complexes **2 - 4** with pDNA (100 ng). Legend: MM – Marker of molecular weights lambda *Hind III*; 1 – pUC18 cleaved by *Hind III* after 2 h of incubation; 2 – pUC18 solubilized in the 5 mM Tris-HCl, 50 mM NaCl buffer, pH 7.02 after 24 h incubation; 3 – pUC18 incubated with the buffer and DMSO 0.1% (v/v) for 24 h; 4 – pUC18 incubated with 5 µM of the complex **2** for 24 h; 5 - pUC18 incubated with 25 µM of the complex **2** for 24 h; 6 - pUC18 incubated with 5 µM of the complex **3** for 24 h; 7 - pUC18 incubated with 25 µM of the complex **3** for 24 h; 8 - pUC18 incubated with 5 µM of the complex **4** for 24 h; 9 - pUC18 incubated with 25 µM of the complex **4** for 24 h; 10 - pUC18 cleaved by *Hind III* after 2 h of incubation; 11 - pUC18 solubilized in the 5 mM Tris-HCl, 50 mM NaCl buffer, pH 7.02 after 24 h incubation; 12 - pUC18 incubated with the buffer and DMSO 0.1% (v/v) for 24 h; 13 - pUC18 incubated with 50 µM of the complex **2** for 24 h; 14 - pUC18 incubated with 100 µM of the complex **2** for 24 h; 15 - pUC18 incubated with 50 µM of the complex **3** for 24 h; 16 - pUC18 incubated with 100 µM of the complex **3** for 24 h; 17 - pUC18 incubated with 50 µM of the complex **4** for 24 h; 18 - pUC18 incubated with 100 µM of the complex **4** for 24 h; N – Isoform nicked; L – Linear isoform; SC – supercoiled isoform.

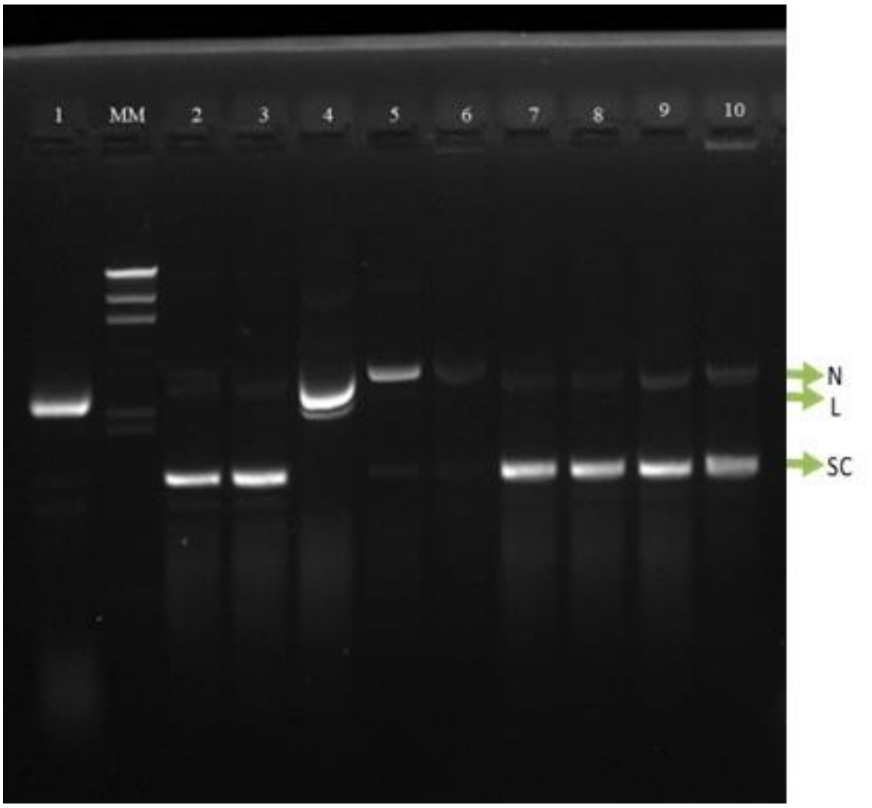

Figure S13. Electrophoresis in 0.8% (w/v) agarose gel in TAE buffer at 70 V constant voltage for 1h with the samples from the interaction of 25 µM of the complexes **2 - 4** with pDNA (100 ng) and NaN_3. Legend: MM – Marker of molecular weights lambda *Hind III*; 1 – pUC18 cleaved by *Hind III* after 2 h of incubation; 2 – pUC18 solubilized in the 5 mM Tris-HCl, 50 mM NaCl buffer, pH 7.02 after 24 h incubation; 3 – pUC18 incubated with the buffer and DMSO 0.1% (v/v) for 24 h; 4 – pUC18 incubated with 25 µM of the complex **2** for 24 h; 5 - pUC18 incubated with 25 µM of the complex **3** for 24 h; 6 - pUC18 incubated with 25 µM of the complex **4** for 24 h; 7 - pUC18 incubated with 50 µM of the NaN_3 for 24 h; 8 - pUC18 incubated with 25 µM of the complex **2** and 50 µM of NaN_3 for 24 h; 9 - pUC18 incubated with 25 µM of the complex **3** and 50 µM of NaN_3 for 24 h; 10 - pUC18 incubated with 25 µM of the complex **4** and 50 µM of NaN_3 for 24 h; N – Isoform nicked; L – Linear isoform; SC – supercoiled isoform.

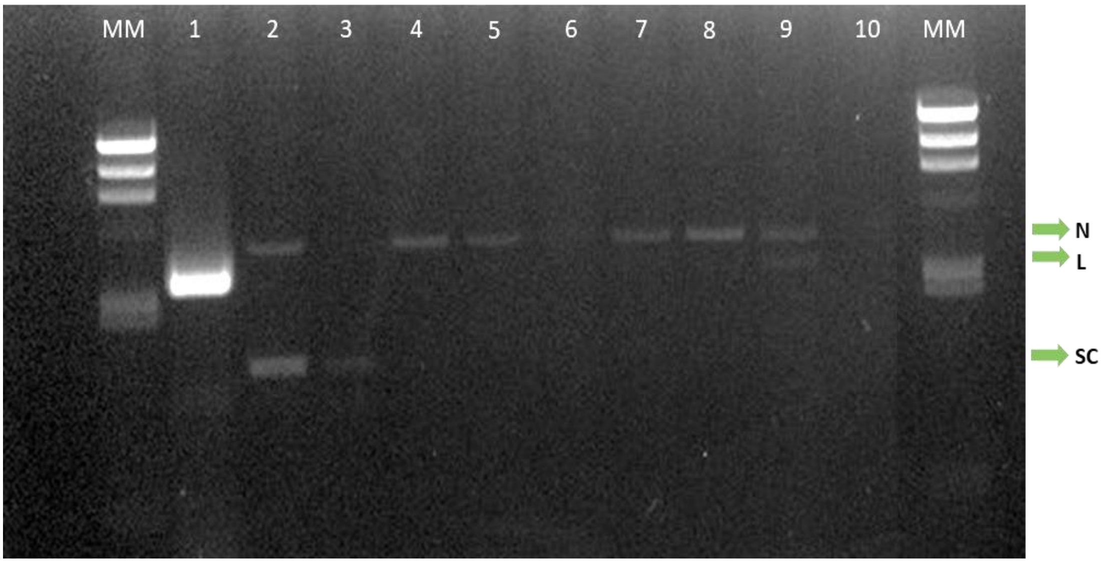

Figure S14. Electrophoresis in 0.8% (w/v) agarose gel in TAE buffer at 70 V constant voltage for 1h with the samples from the interaction of different concentrations (25 µM) of the complexes **2 - 4** with pDNA (100 ng) and 4 units of Catalase. Legend: MM – Marker of molecular weights lambda *Hind III*; 1 – pUC18 cleaved by *Hind III* after 2 h of incubation; 2 – pUC18 solubilized in the 5 mM Tris-HCl, 50 mM NaCl buffer, pH 7.02 after 24 h incubation; 3 – pUC18 incubated with the buffer and DMSO 0.1% (v/v) for 24 h; 4 – pUC18 incubated with 25 µM of the complex **3** for 24 h; 5 - pUC18 incubated with 25 µM of the complex **2** for 24 h; 6 - pUC18 incubated with 25 µM of the complex **4** for 24 h; 7 - pUC18 incubated with25 µM of the complex **2** and 4 units of Catalase for 24 h; 8 - pUC18 incubated with 25 µM of the complex **3** and 4 units of Catalase for 24 h; 9 - pUC18 incubated with 50 µM of the complex **2** and 4 units of Catalase for 24 h; 10 - pUC18 incubated with 25 µM of the complex **4** and 4 units of Catalase for 24 h; N – Isoform nicked; L – Linear isoform; SC – supercoiled isoform.

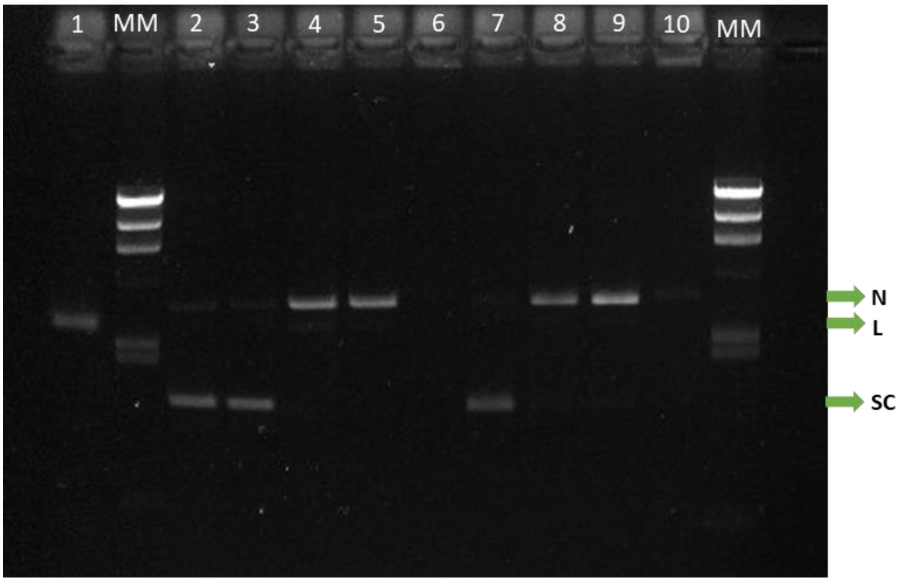

Figure S15. Electrophoresis in 0.8% (w/v) agarose gel in TAE buffer at 70 V constant voltage for 1 h with the samples from the interaction of different concentrations (25 µM) of the complexes **2 - 4** with pDNA (100 ng) and D_2O for 24 h. Legend: MM – Marker of molecular weights lambda *Hind III*; 1 – pUC18 cleaved by *Hind III* after 2 h of incubation; 2 – pUC18 solubilized in the 5 mM Tris-HCl, 50 mM NaCl buffer, pH 7.02 after 24 h incubation; 3 – pUC18 incubated with the buffer and DMSO 0.1% (v/v) for 24 h; 4 – pUC18 incubated with 25 µM of the complex **2** for 24 h; 5 - pUC18 incubated with 25 µM of the complex **3** for 24 h; 6 - pUC18 incubated with 25 µM of the complex **4** for 24 h; 7 - pUC18 incubated with D_2O for 24 h; 8 - pUC18 incubated with 25 µM of the complex **2** and D_2O for 24 h; 9 - pUC18 incubated with 25 µM of the complex **3** and D_2O for 24 h; 10 - pUC18 incubated with 25 µM of the complex **4** and D_2O for 24 h; N – Isoform nicked; L – Linear isoform; SC – supercoiled isoform.

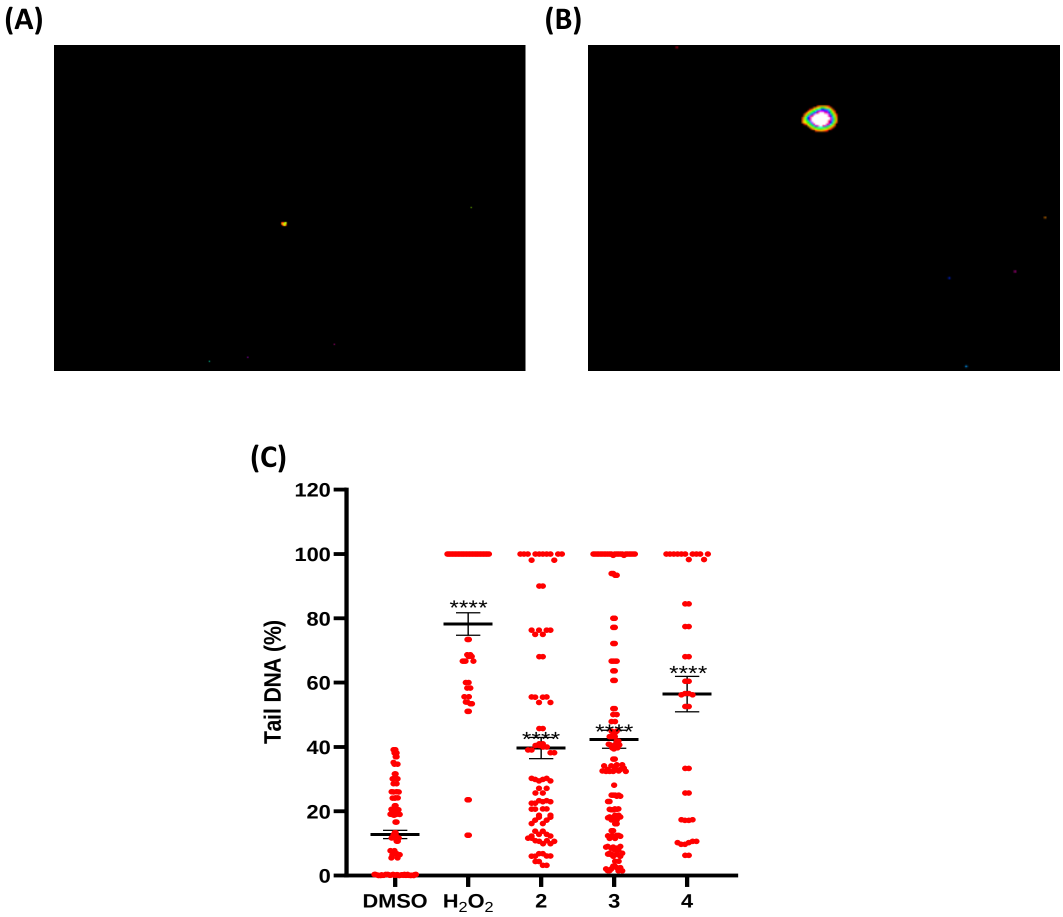

Figure S16. Comet assay in A2780 cells after exposure to 0.1% (v/v) DMSO and complexes **2 – 4** for 12 h and 0.05% H_2O_2 for 30 min at RT. **(A)** Comet image after exposure to 0.1% (v/v) DMSO, vehicle control, using CometScore 2.1 software; **(B)** Comet image after exposure to complex **3,** using CometScore 2.1 software; **(C)** Representation of tail DNA (%) after exposure to 0.1% (v/v) DMSO and complexes **2 – 4** for 12 h and 0.05% H_2O_2 for 30 min at RT. Data expressed as mean ± SEM.

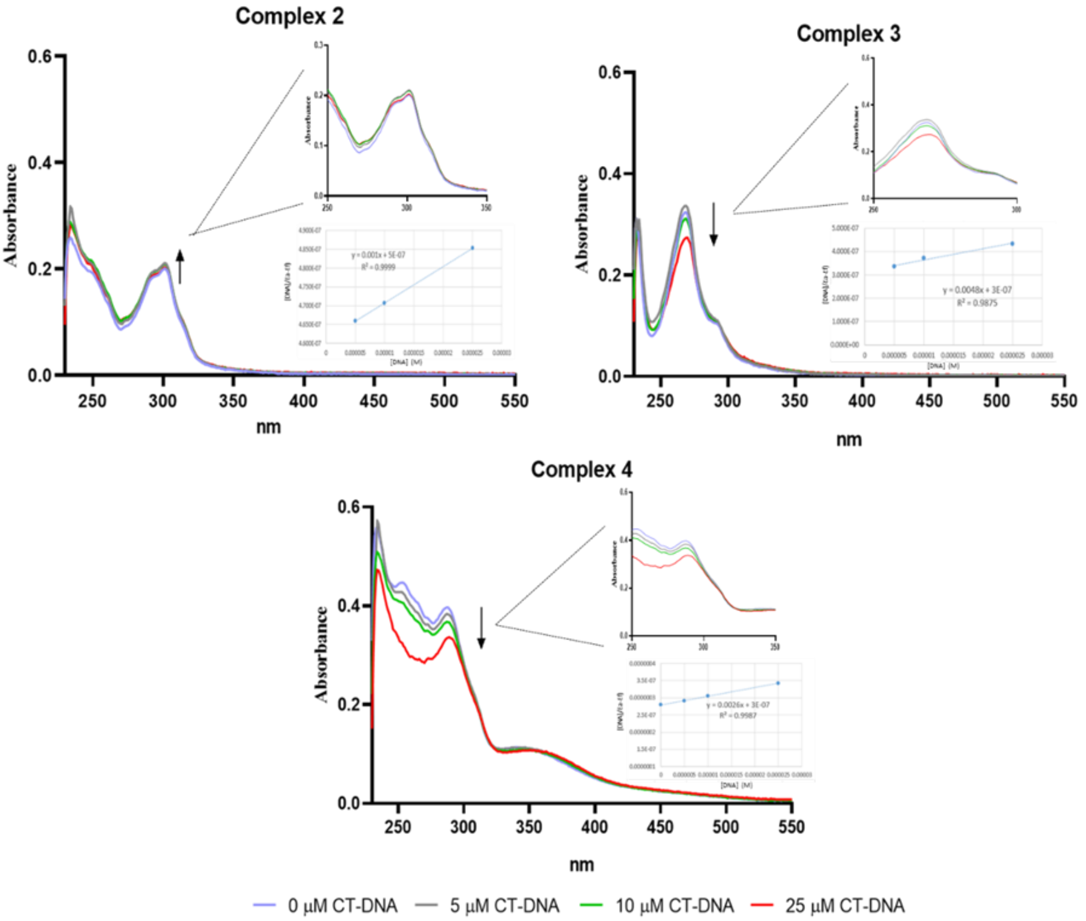

Figure S17. Absorption spectra of Co(III) complexes **(A) 2, (B) 3** and **(C) 4,** (all at 25 µM) after 24h of incubation in the absence or presence of increasing amounts of CT-DNA (0-25 µM) at 37 °C.

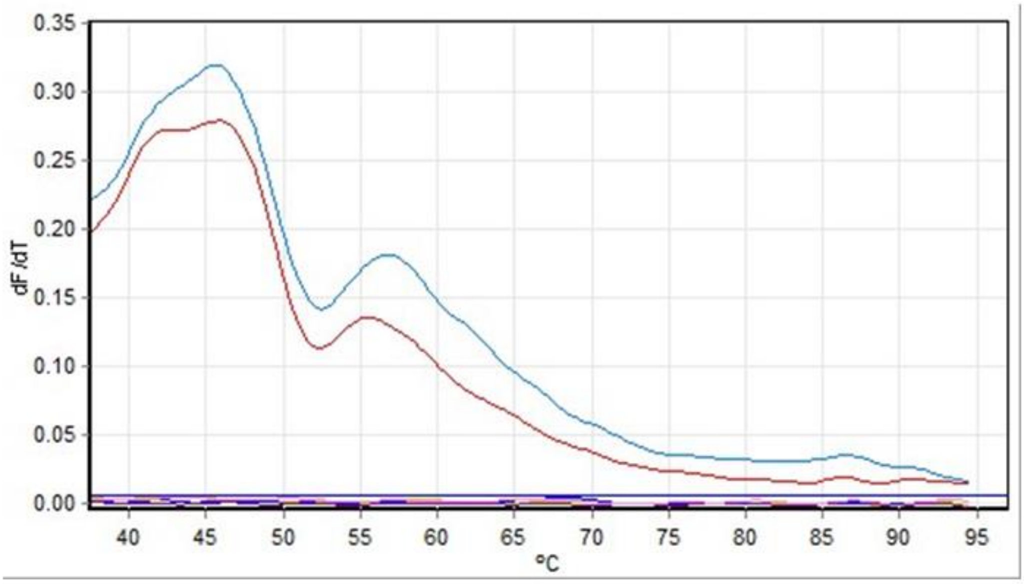

No.	Color	Name	Peak 1	Peak 2	Peak 3	Peak 4
1		CT-DNA + evagreen	42.5	45.8	55.5	86.3
2		CT-DNA + **3** (50µM) + evagreen				
3		CT-DNA + **2** (50µM) + evagreen				
4		CT-DNA + **4** (50µM) + evagreen				
5		Only DMSO 0.1% + evagreen				
6		CT-DNA + DMSO 0.1% + evagreen	45.5	56.8	86.5	

Figure S18. Melting profile of 10 µM CT-DNA in presence and absence of 10 µM complexes **2** – **4** or 0.1% DMSO recorded after 1h30 of exposure to the cobalt complexes in a real time PCR machine (Corbet RotorGene).

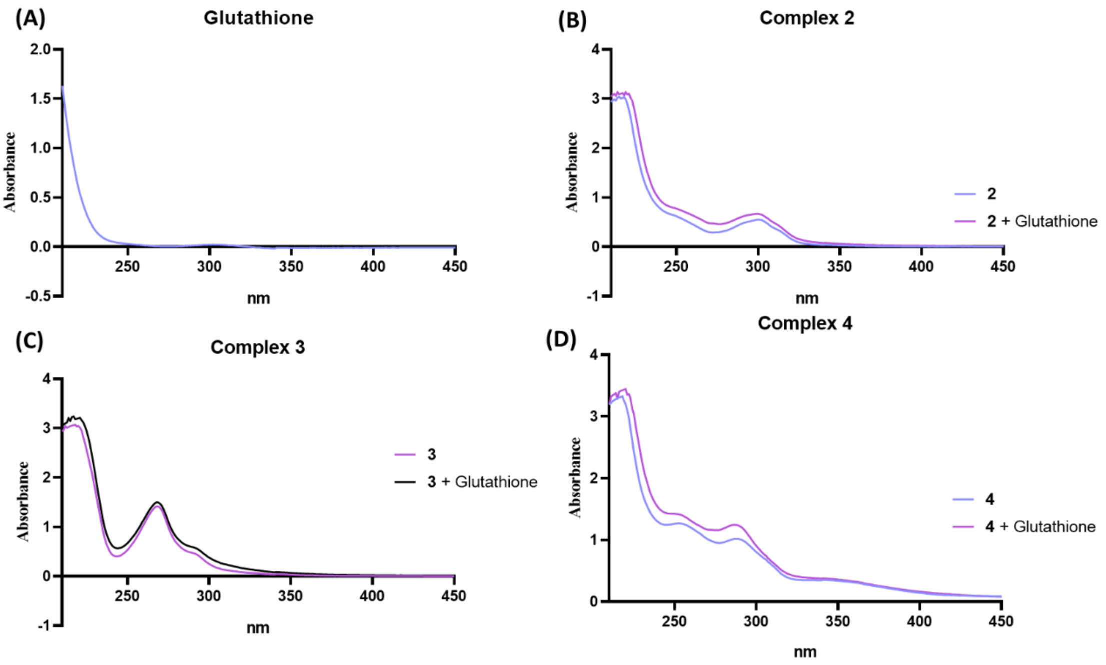

Figure S19. UV-Vis spectra of 50 µM Glutathione **(A)** and 50 µM Glutathione exposed to the 50 µM of the complexes **2 (A)**, **3 (B)** and **4 (C)** after 24 h incubation**.**

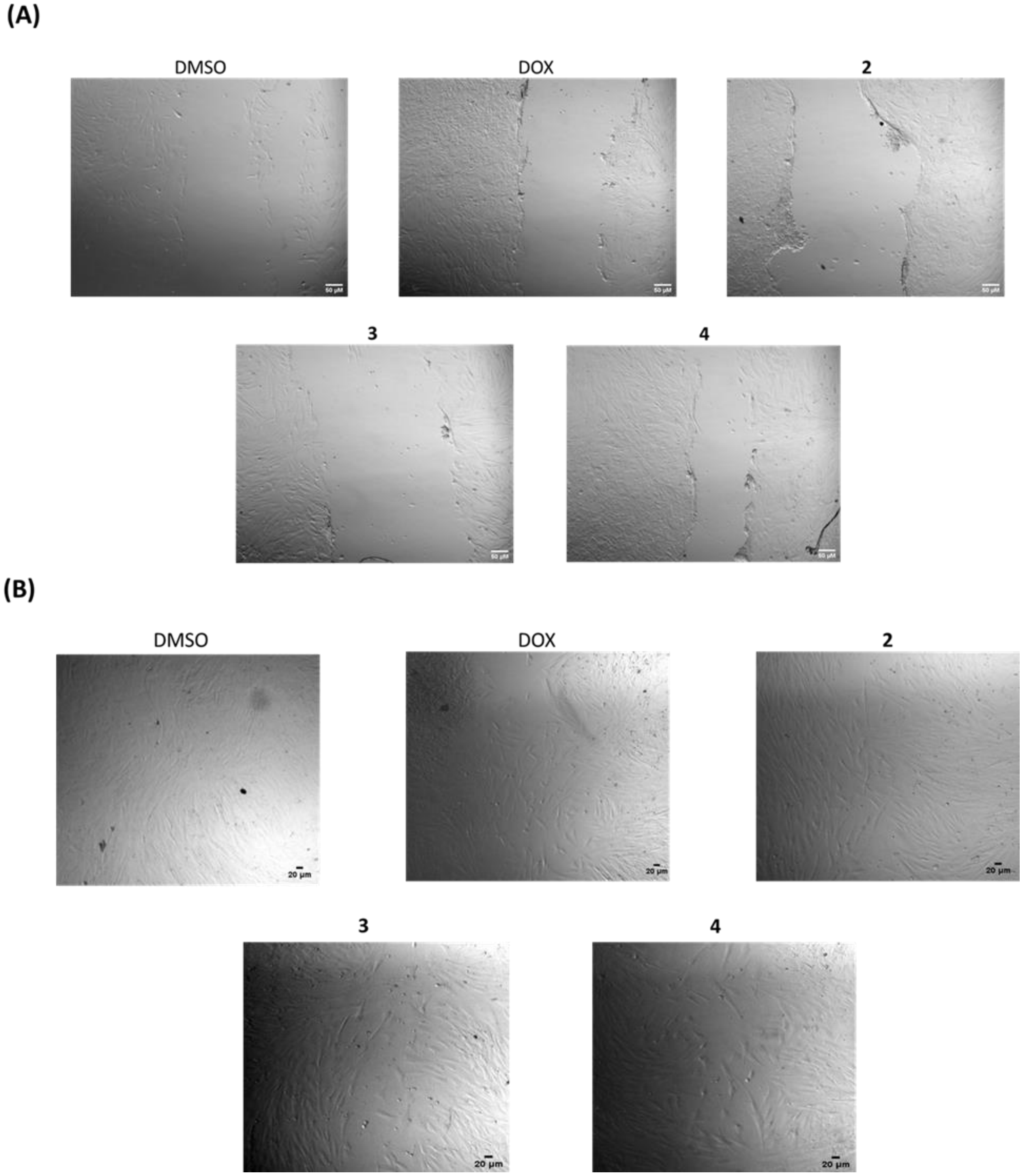

Figure S20. Representative cell migration assay microscopy images. **(A)** Fibroblasts images after 0h of incubation with the IC_{50} concentrations of the complexes **2**, **3** and **4**, 0.4 % DOX or 0.1% (v/v) DMSO. **(B)** Fibroblasts images after 24h of incubation with the IC_{50} concentrations of the complexes **2**, **3** and **4**, 0.4 % DOX or 0.1% (v/v) DMSO. Images obtained through Ti-U Eclipse inverted microscope (Nikon) and respective software, NIS Elements Basic software 3.1 (Nikon). The scratch images were analyzed via ImageJ software.

Supplementary NMR data

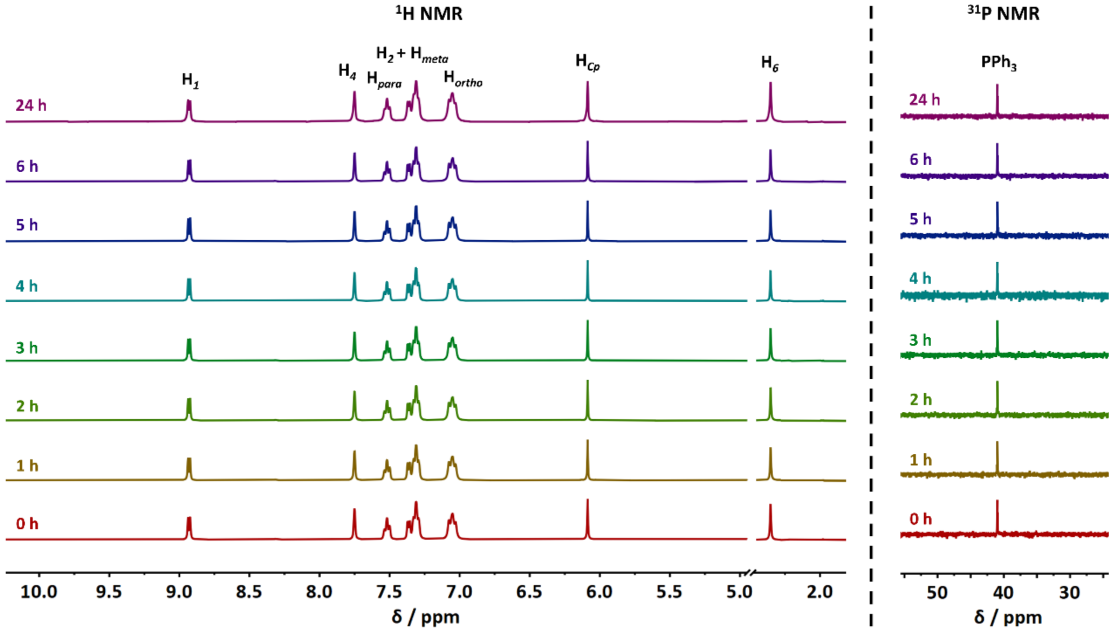

Figure S21. Evaluation of the stability of $[CoCp(PPh_3)(Me_2bipy)][(CF_3SO_3)_2]$ (**2**) in 90% D_2O/10% DMSO-d$_6$ solution (2.5 mM) over 24 hours, by ^{1}H NMR (left) and ^{31}P{H} NMR (right).

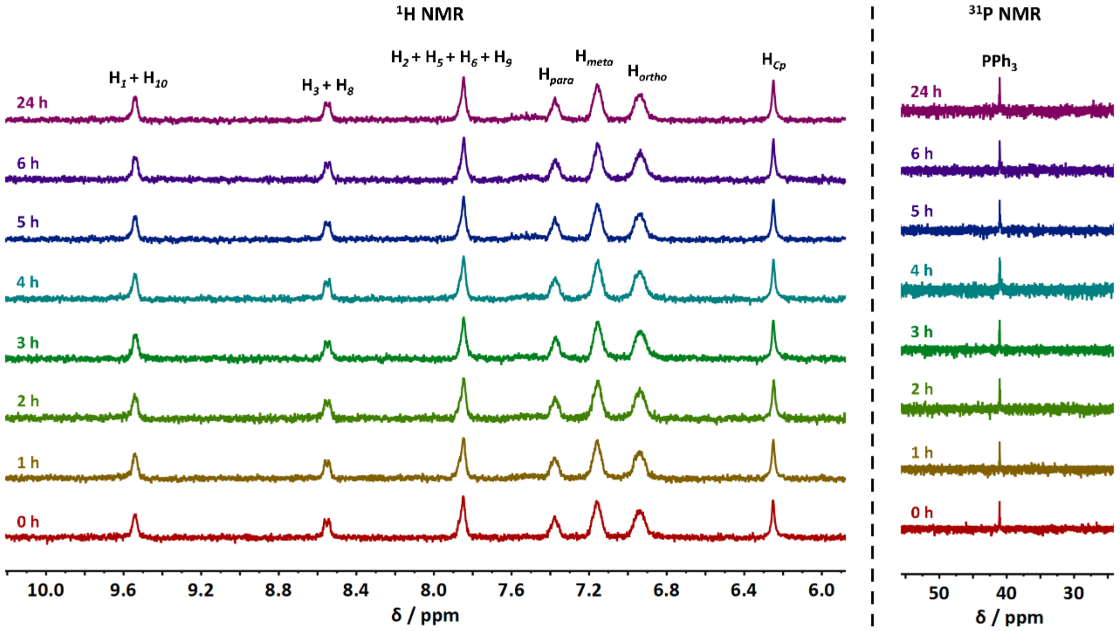

Figure S22. Evaluation of the stability of $[CoCp(PPh_3)(phen)][(CF_3SO_3)_2]$ (**3**) in 90% D_2O/10% DMSO-d_6 solution (2.5 mM) over 24 hours, by ^{1}H NMR (left) and ^{31}P{H} NMR (right).

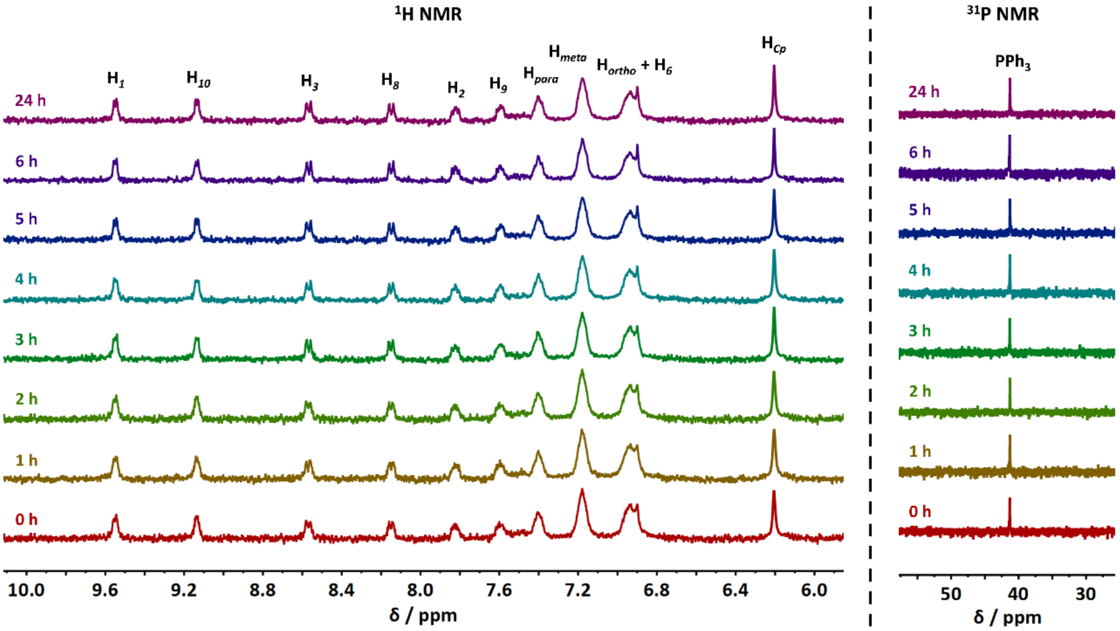

Figure S23. Evaluation of the stability of [CoCp(PPh$_3$)(NH$_2$phen)][(CF$_3$SO$_3$)$_2$] (**4**) in 90% D$_2$O/10% DMSO-d_6 solution (2.5 mM) over 24 hours, by ^{1}H NMR (left) and ^{31}P{H} NMR (right).

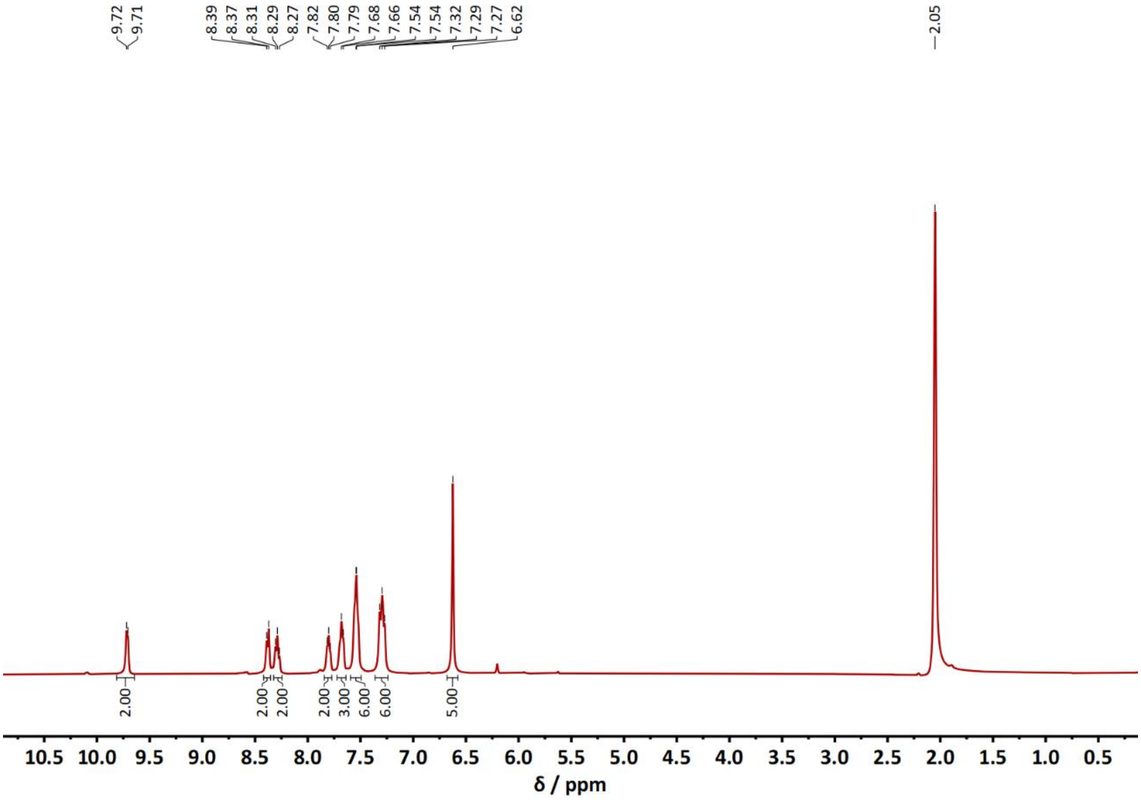

Figure S24. ^{1}HNMR spectrum of complex [CoCp(PPh$_3$)(bipy)][(CF$_3$SO$_3$)$_2$] (**1**) in (CD$_3$)$_2$CO.

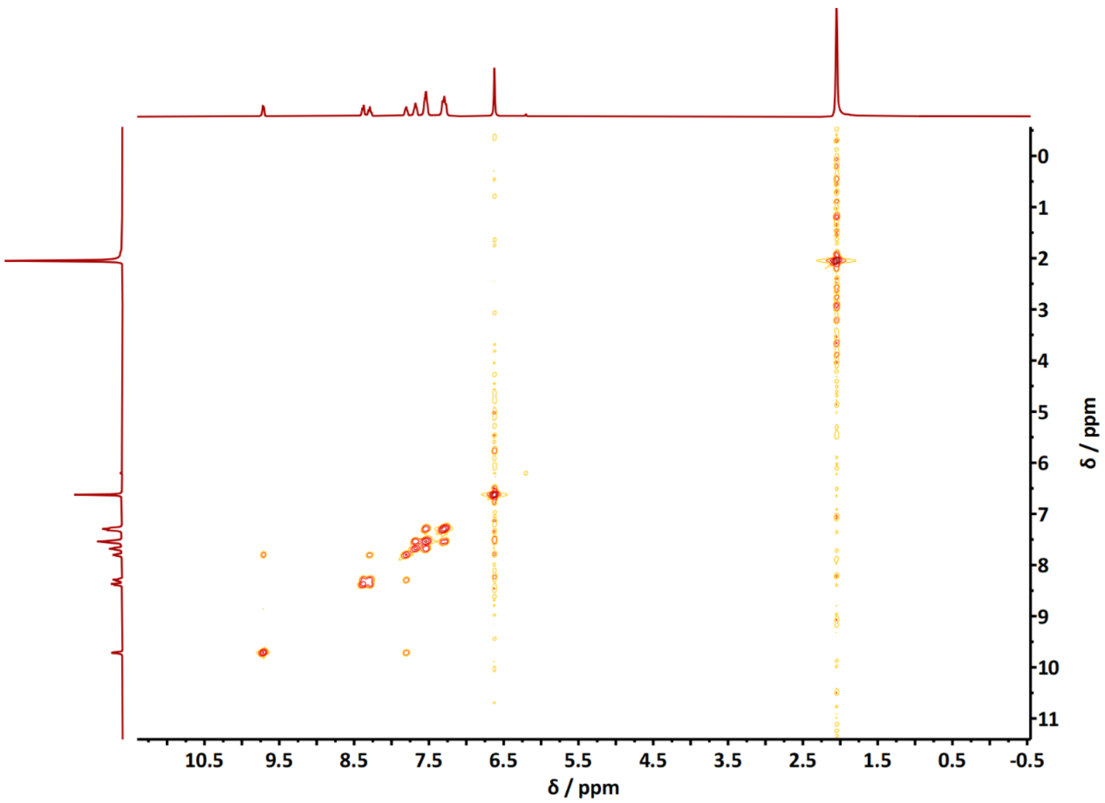

Figure S25. cosy-NMR spectrum of complex [CoCp(PPh$_3$)(bipy)][(CF$_3$SO$_3$)$_2$] (**1**) in (CD$_3$)$_2$CO.

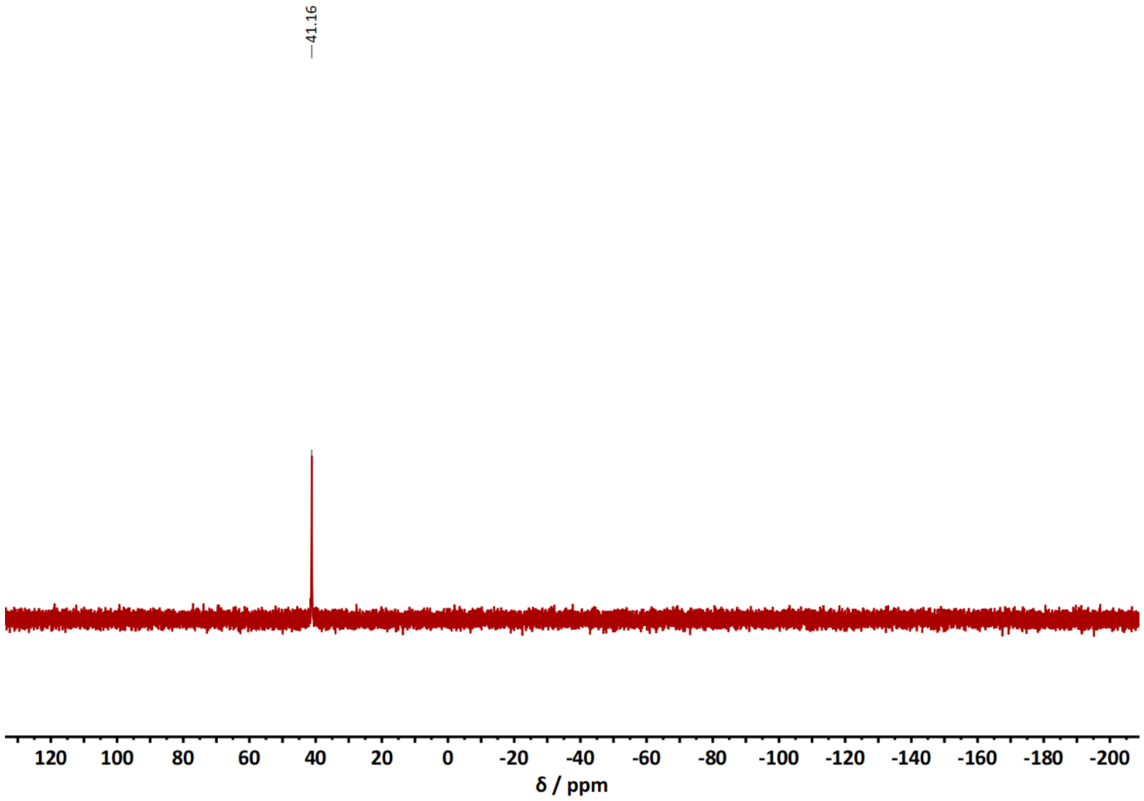

Figure S26. ^{31}P NMR spectrum of complex [CoCp(PPh$_3$)(bipy)][(CF$_3$SO$_3$)$_2$] (**1**) in (CD$_3$)$_2$CO.

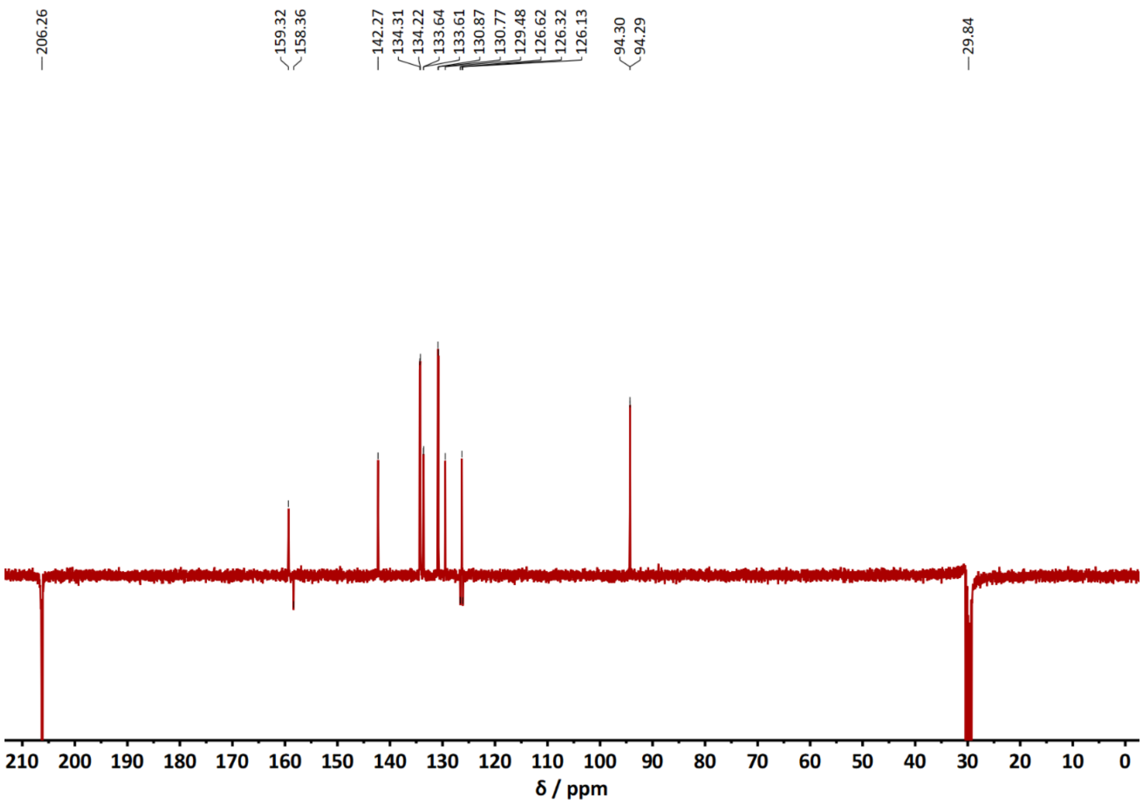

Figure S27. ^{13}C NMR spectrum of complex $[CoCp(PPh_3)(bipy)][(CF_3SO_3)_2]$ (**1**) in $(CD_3)_2CO$.

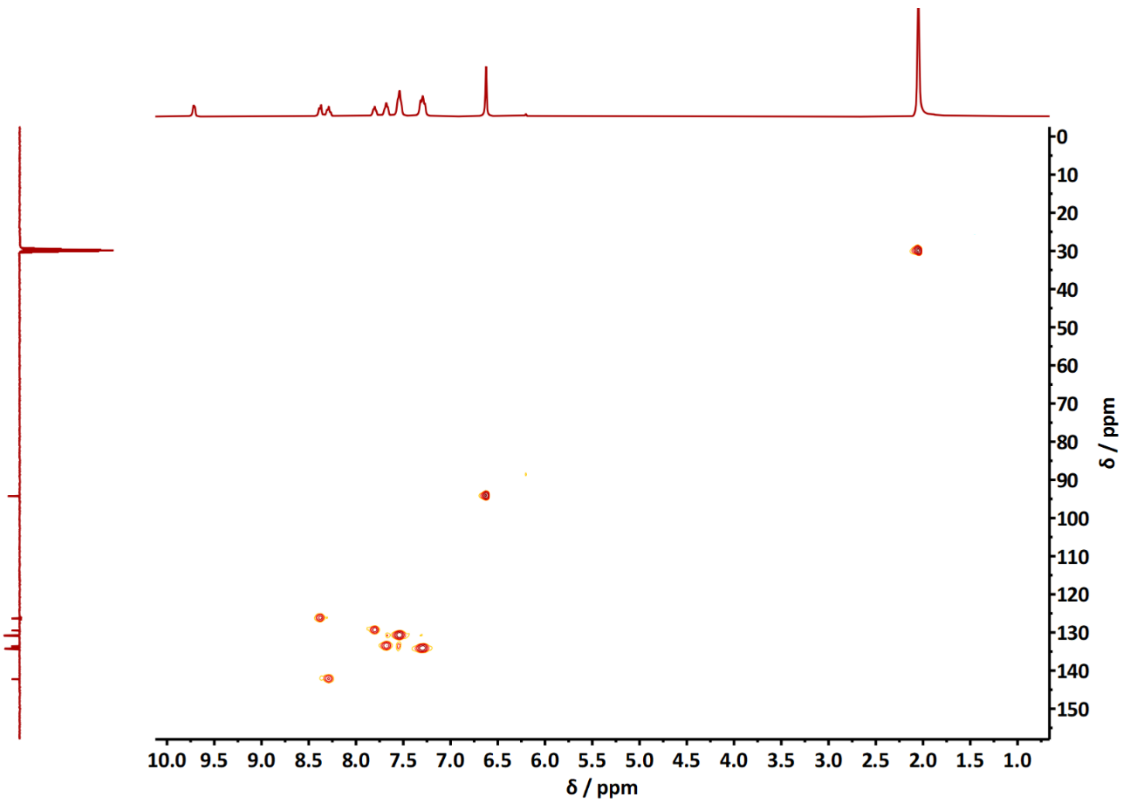

Figure S28. HSQC-NMR spectrum of complex $[CoCp(PPh_3)(bipy)][(CF_3SO_3)_2]$ (**1**) in $(CD_3)_2CO$.

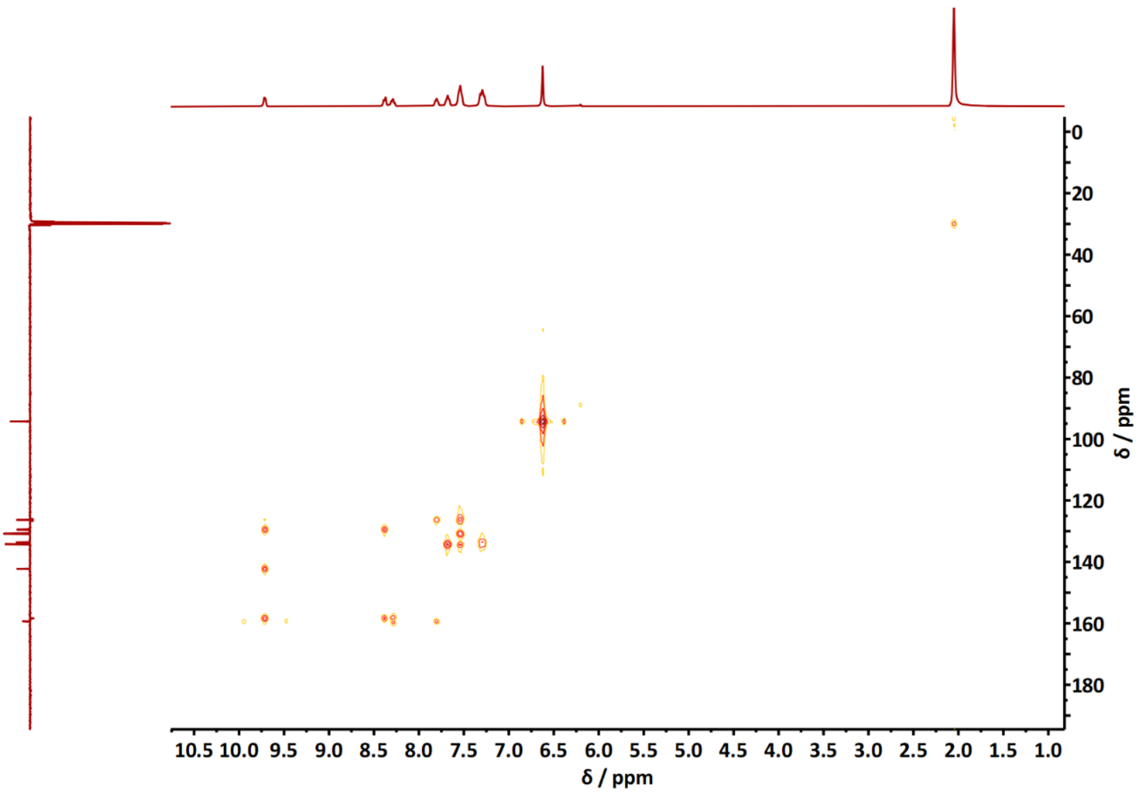

Figure S29. HMBC-NMR spectrum of complex $[CoCp(PPh_3)(bipy)][(CF_3SO_3)_2]$ (**1**) in $(CD_3)_2CO$.

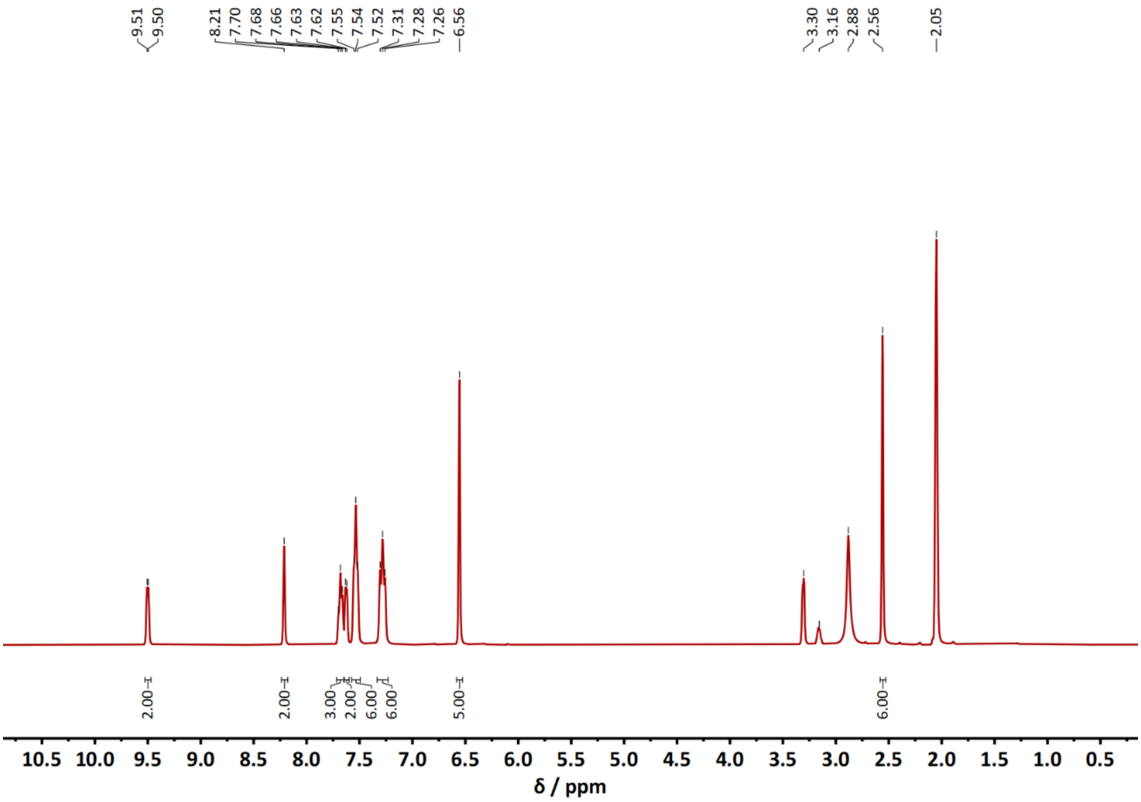

Figure S30. ^1H NMR spectrum of complex $[CoCp(PPh_3)(Me_2bipy)][(CF_3SO_3)_2]$ (**2**) in $(CD_3)_2CO$.

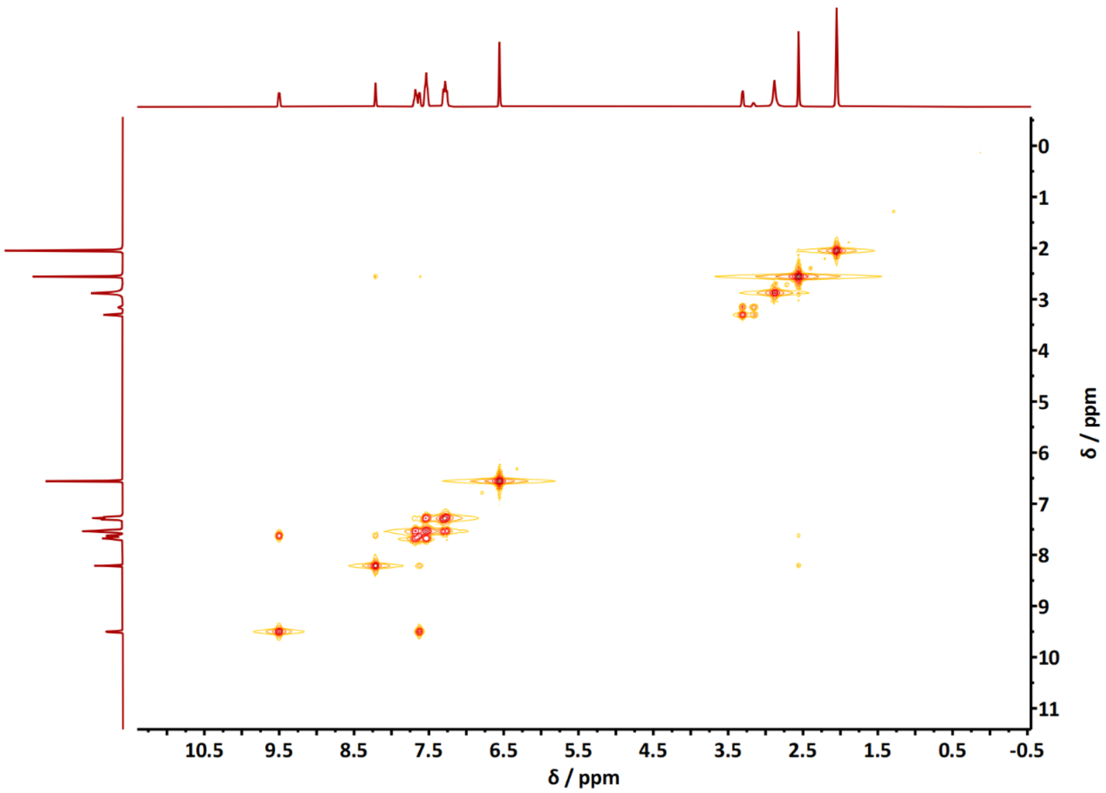

Figure S31. cosy-NMR spectrum of complex $[CoCp(PPh_3)(Me_2bipy)][(CF_3SO_3)_2]$ (**2**) in $(CD_3)_2CO$.

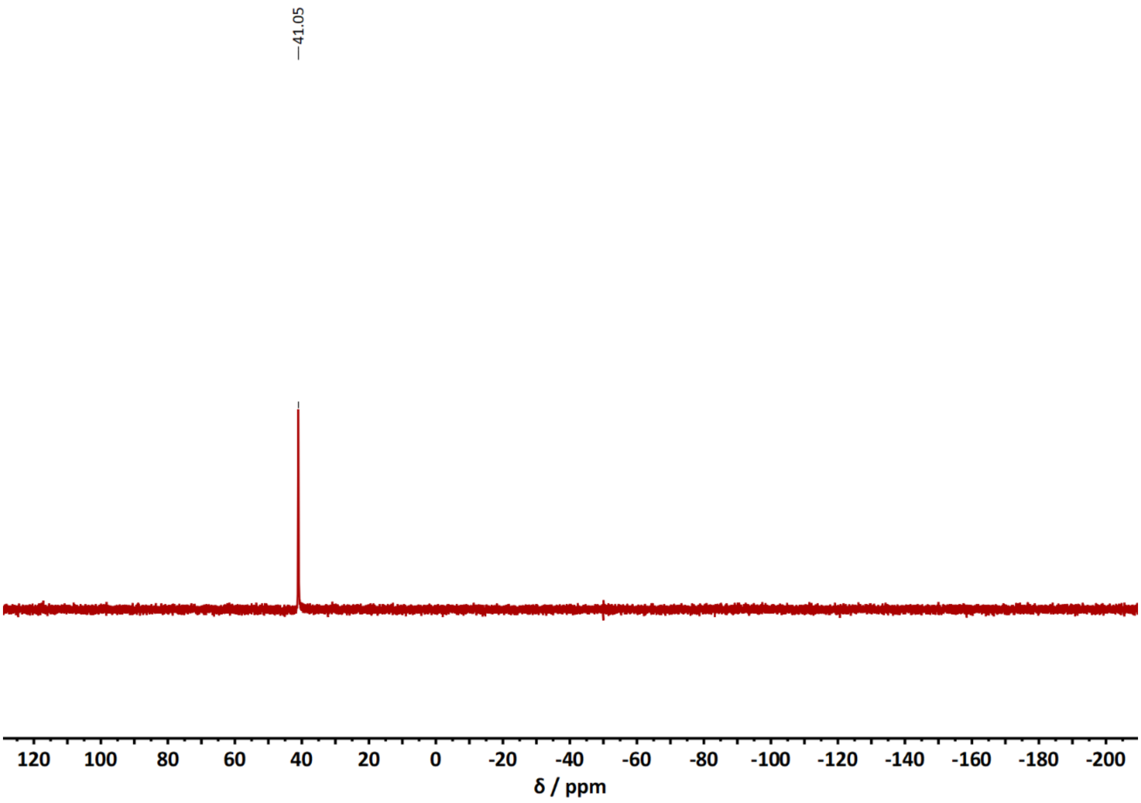

Figure S32. ^{31}P NMR spectrum of complex $[CoCp(PPh_3)(Me_2bipy)][(CF_3SO_3)_2]$ (**2**) in $(CD_3)_2CO$.

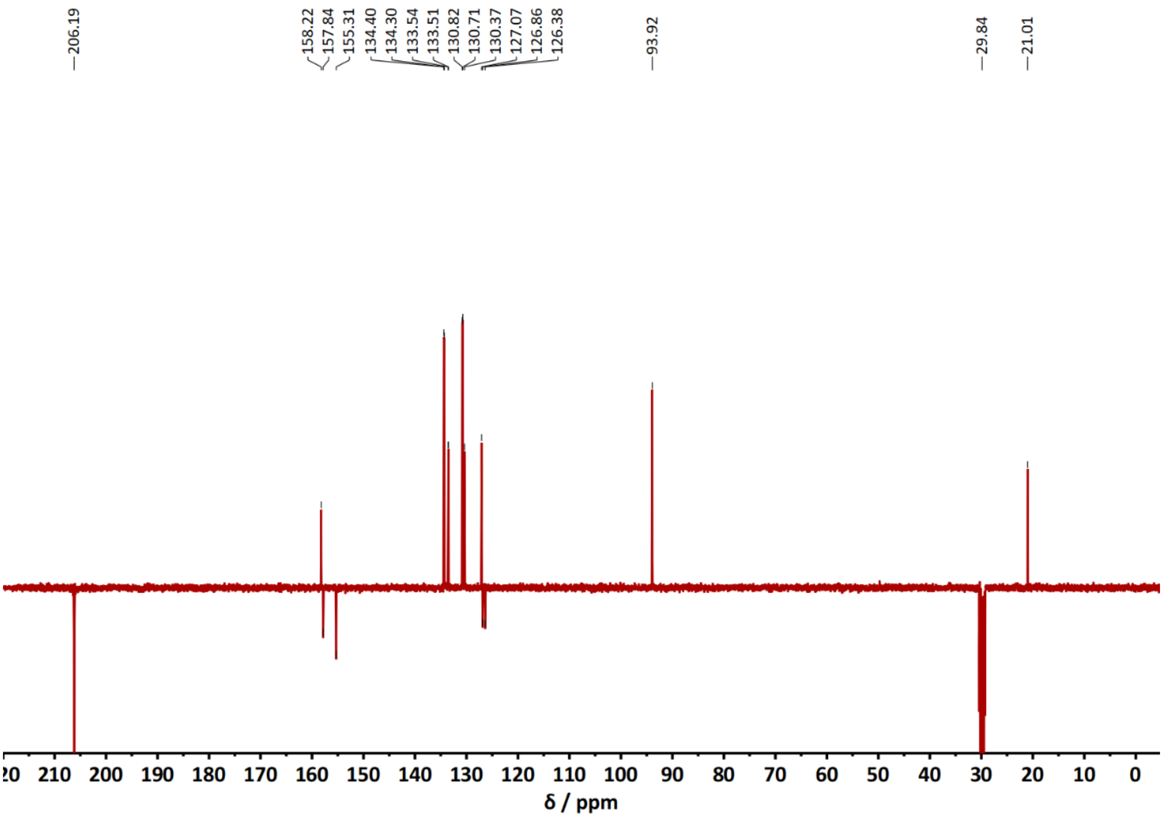

Figure S33. ^{13}C NMR spectrum of complex $[CoCp(PPh_3)(Me_2bipy)][(CF_3SO_3)_2]$ (**2**) in $(CD_3)_2CO$.

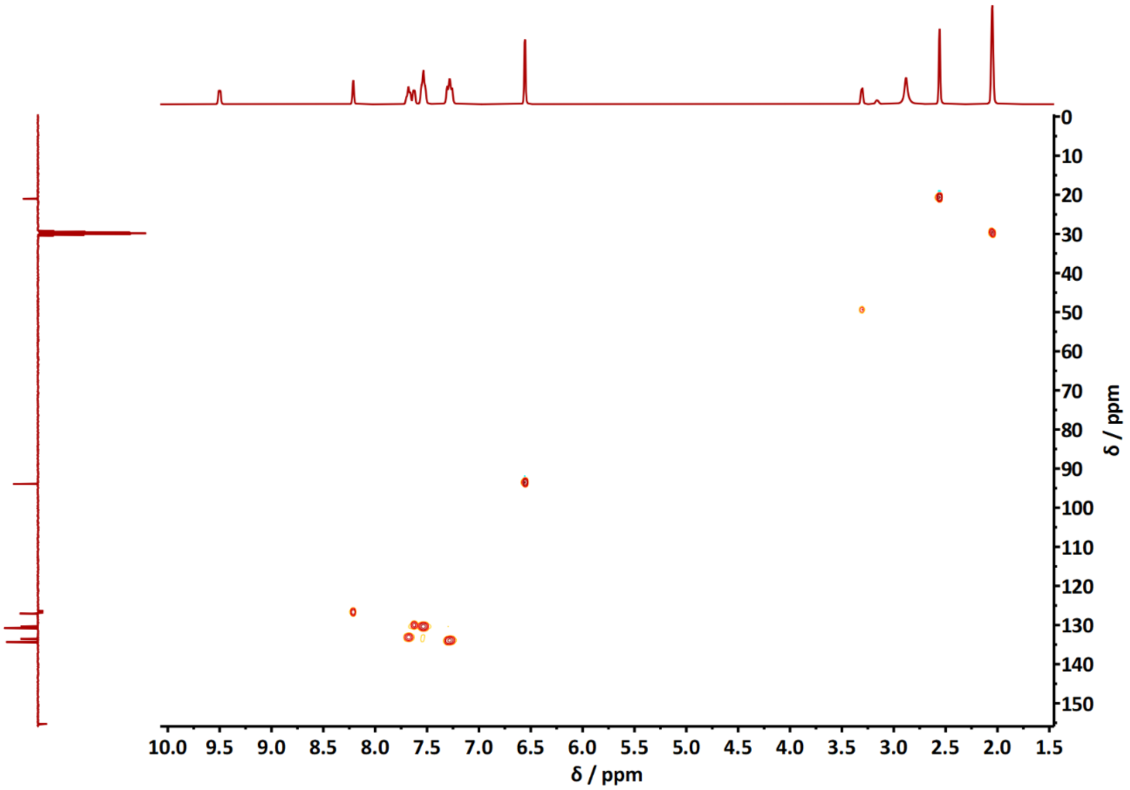

Figure S34. HSQC-NMR spectrum of complex $[CoCp(PPh_3)(Me_2bipy)][(CF_3SO_3)_2]$ (**2**) in $(CD_3)_2CO$.

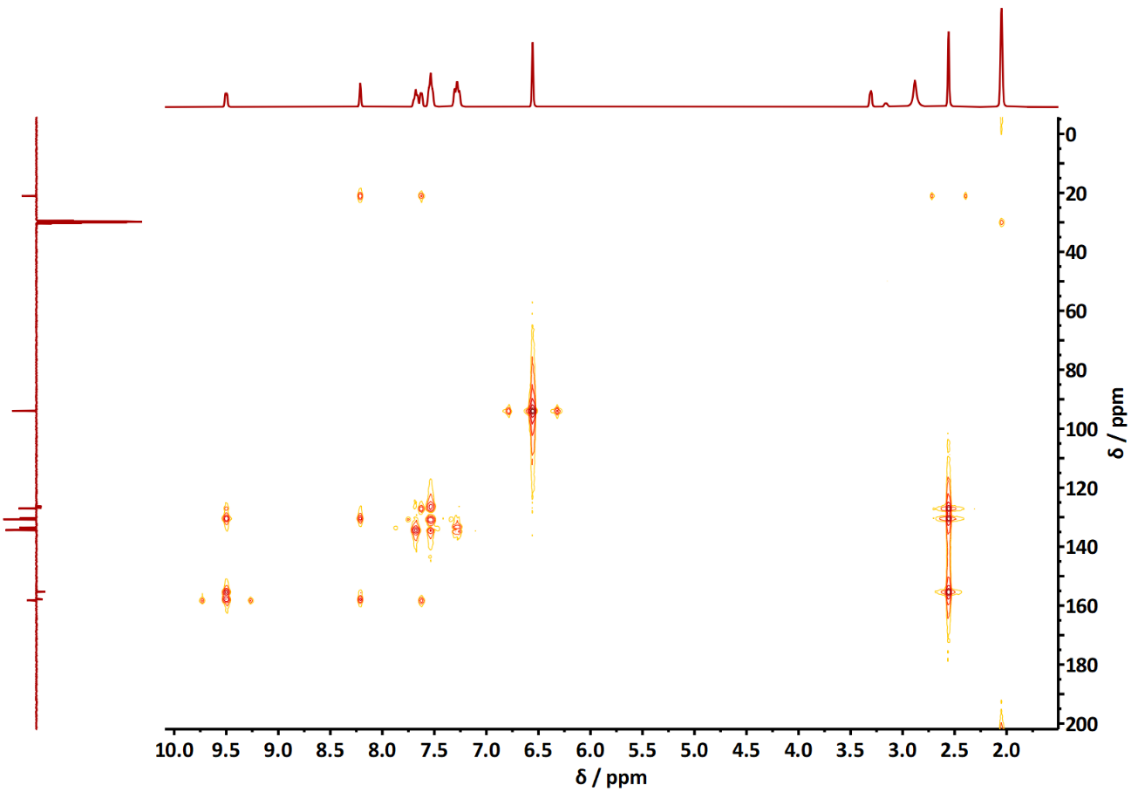

Figure S35. HMBC-NMR spectrum of complex $[CoCp(PPh_3)(Me_2bipy)][(CF_3SO_3)_2]$ (**2**)in $(CD_3)_2CO$.

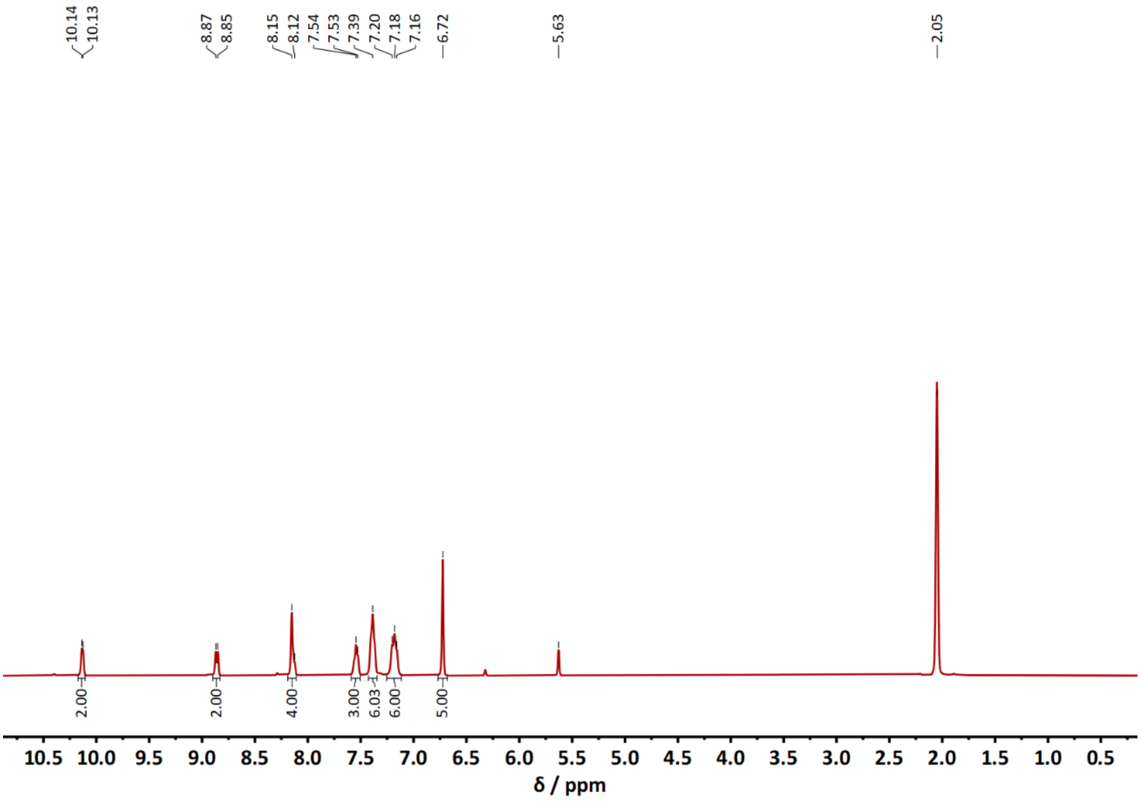

Figure S36. ^{1}H NMR spectrum of complex $[CoCp(PPh_3)(Phen)][(CF_3SO_3)_2]$ (**3**) in $(CD_3)_2CO$.

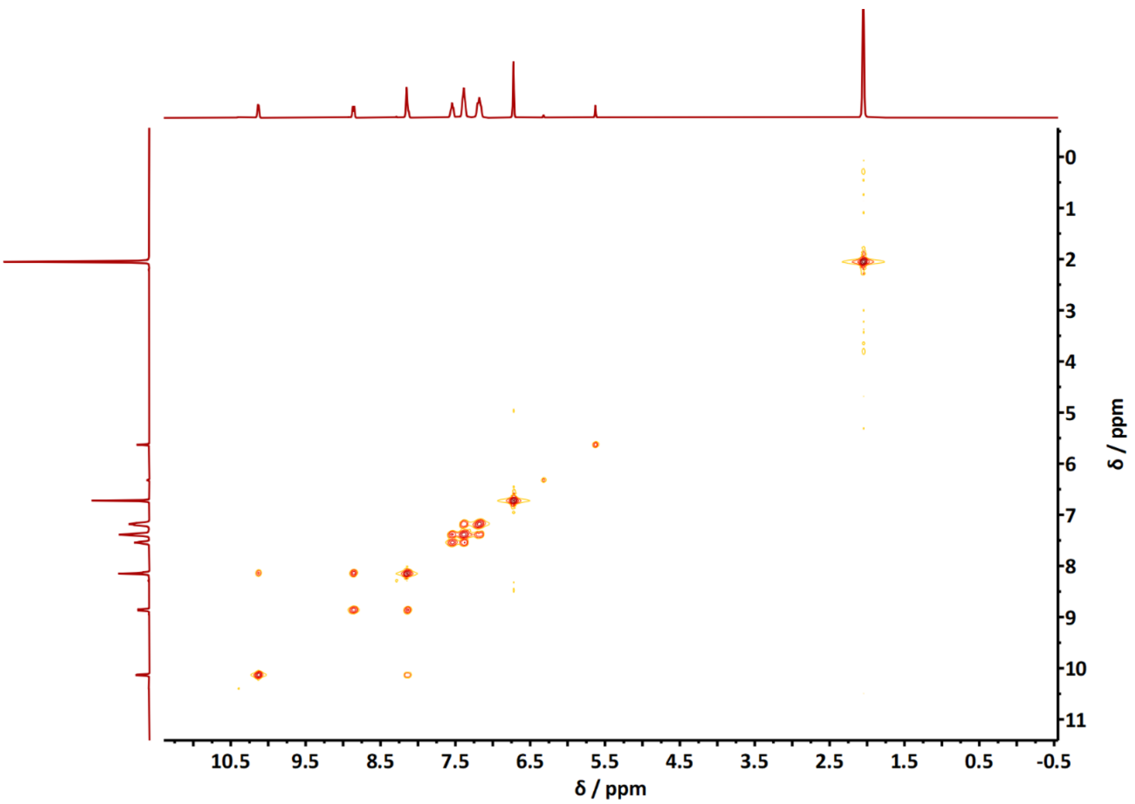

Figure S37. cosy-NMR spectrum of complex $[CoCp(PPh_3)(Phen)][(CF_3SO_3)_2]$ (**3**) in $(CD_3)_2CO$.

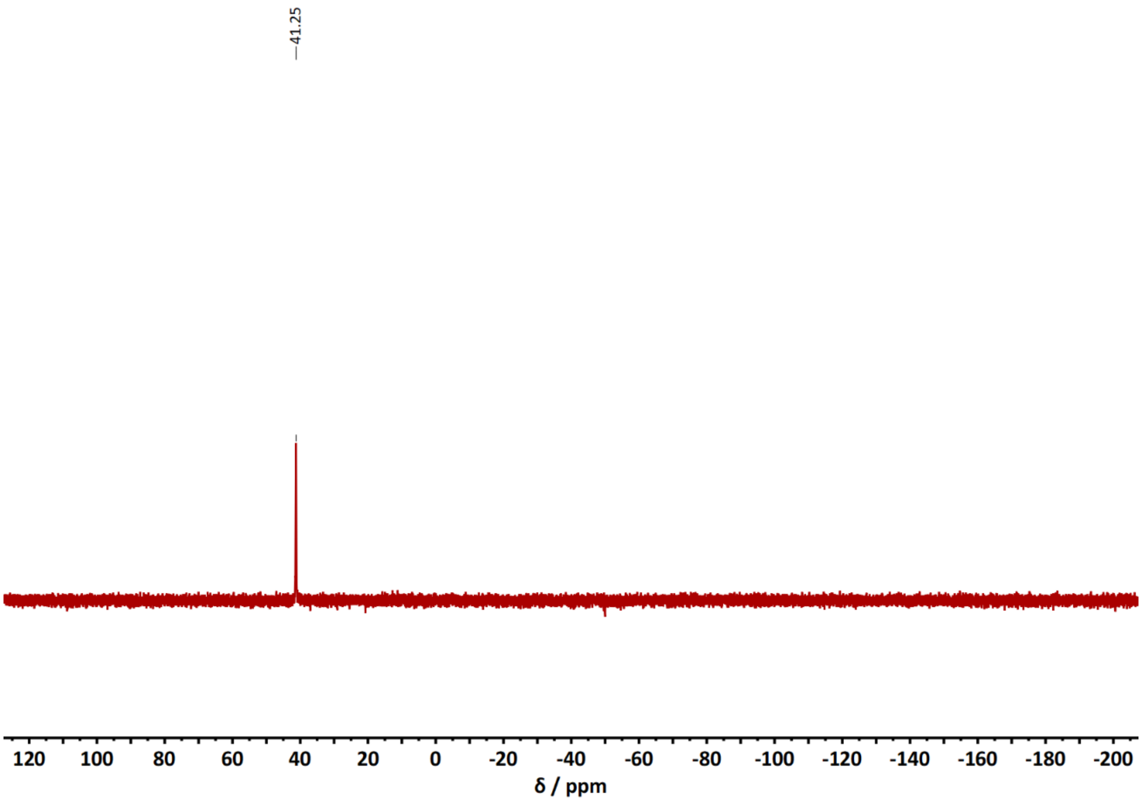

Figure S38. ^{31}P NMR spectrum of complex $[CoCp(PPh_3)(Phen)][(CF_3SO_3)_2]$ (**3**) in $(CD_3)_2CO$.

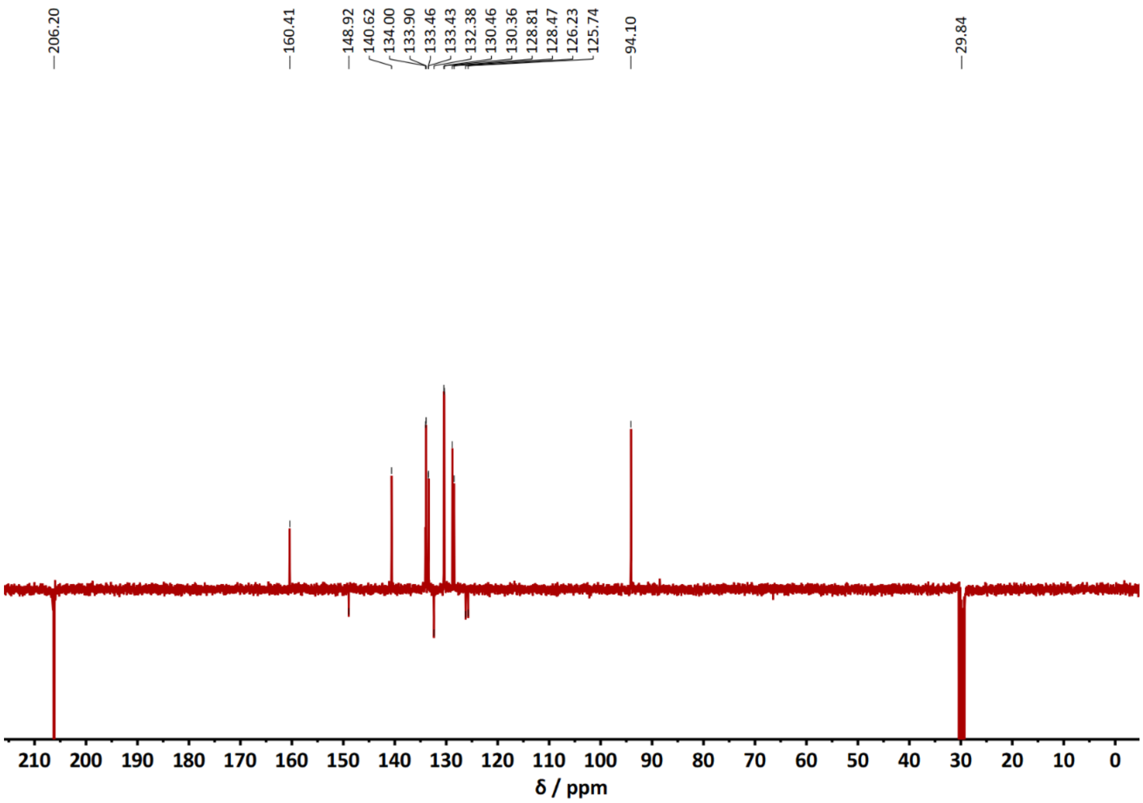

Figure S39. ^{13}C NMR spectrum of complex [CoCp(PPh$_3$)(Phen)][(CF$_3$SO$_3$)$_2$] (**3**) in (CD$_3$)$_2$CO.

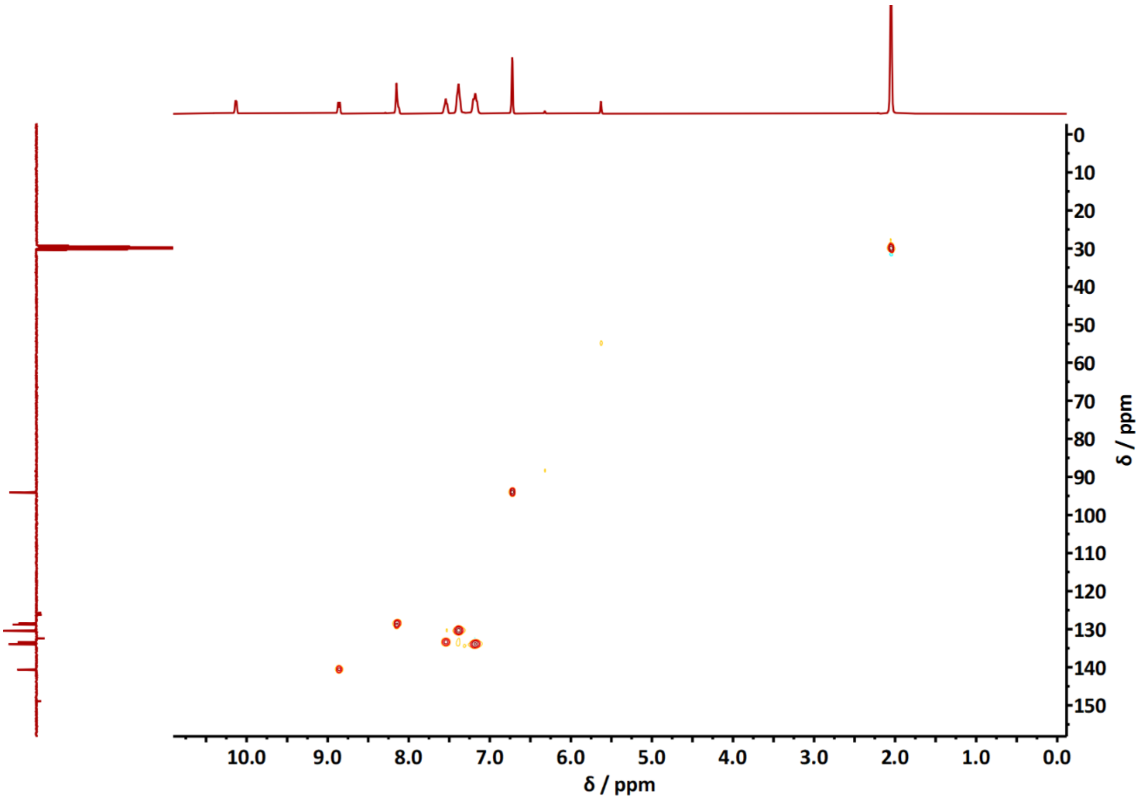

Figure S40. HSQC-NMR spectrum of complex [CoCp(PPh$_3$)(Phen)][(CF$_3$SO$_3$)$_2$] (**3**) in (CD$_3$)$_2$CO.

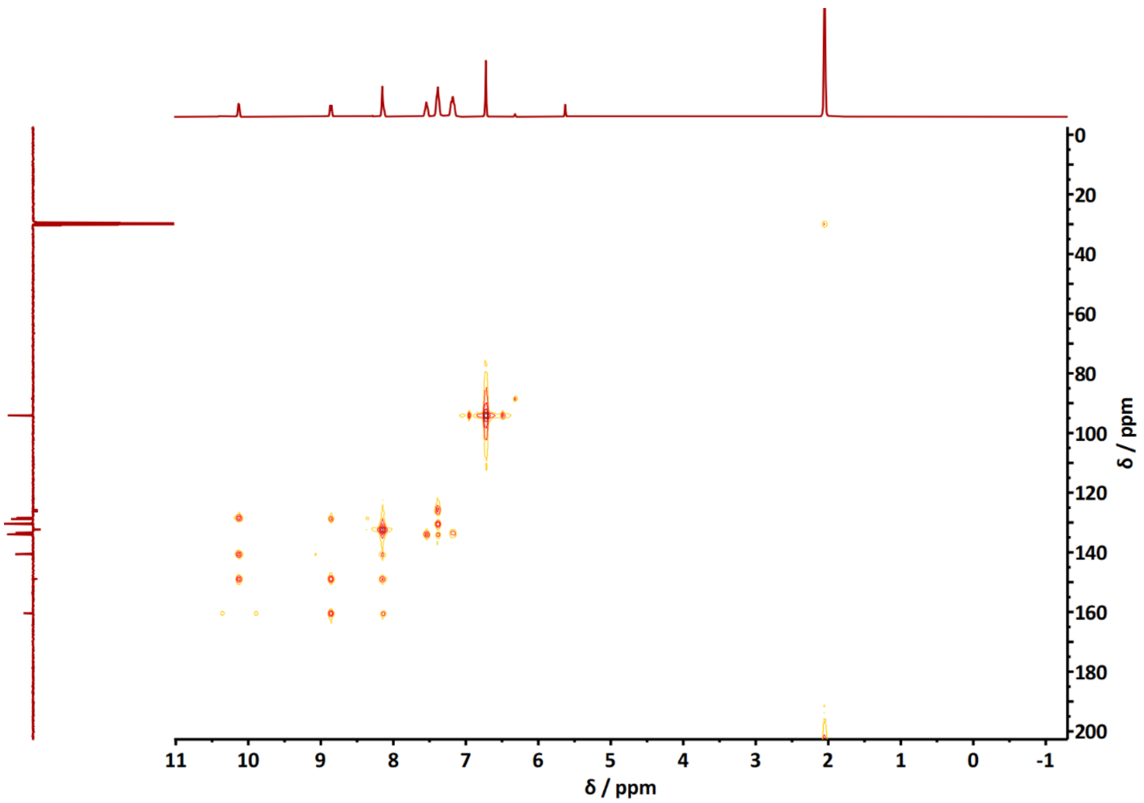

Figure S41. HMBC-NMR spectrum of complex [CoCp(PPh$_3$)(Phen)][(CF$_3$SO$_3$)$_2$] (**3**) in (CD$_3$)$_2$CO.

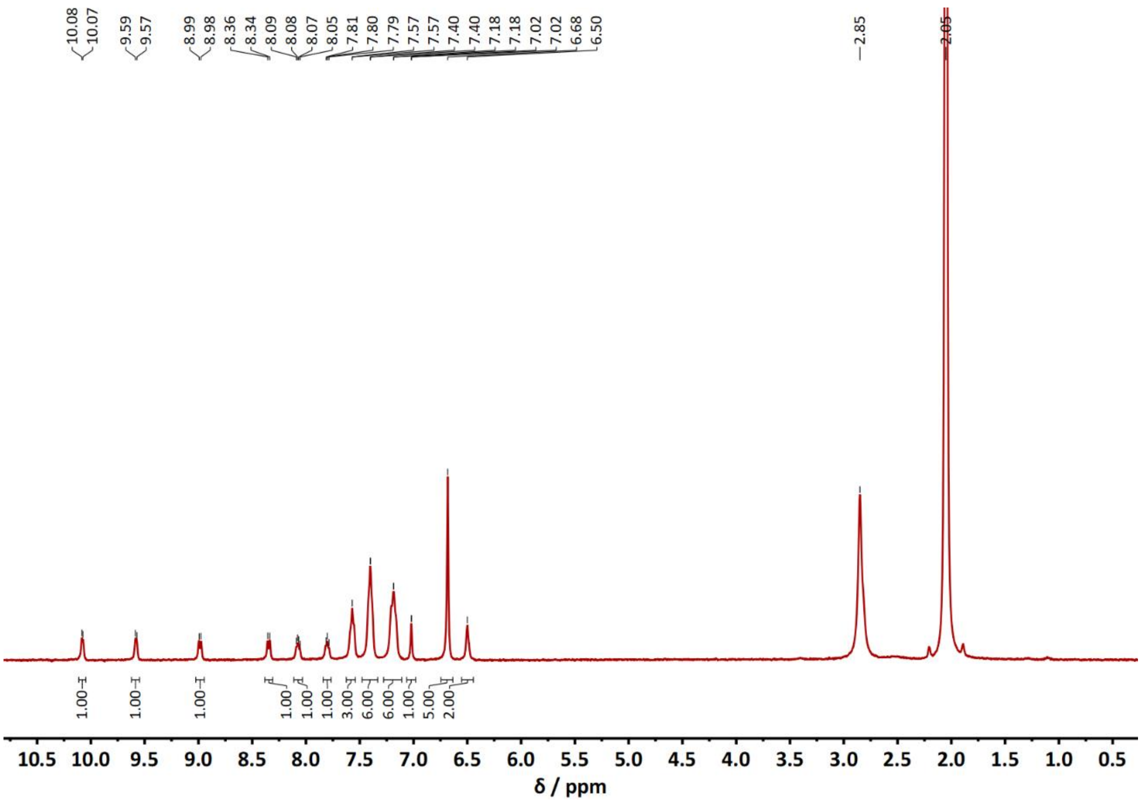

Figure S42. ^{1}H NMR spectrum of complex [CoCp(PPh$_3$)(NH$_2$Phen)][(CF$_3$SO$_3$)$_2$] (**4**) in (CD$_3$)$_2$CO.

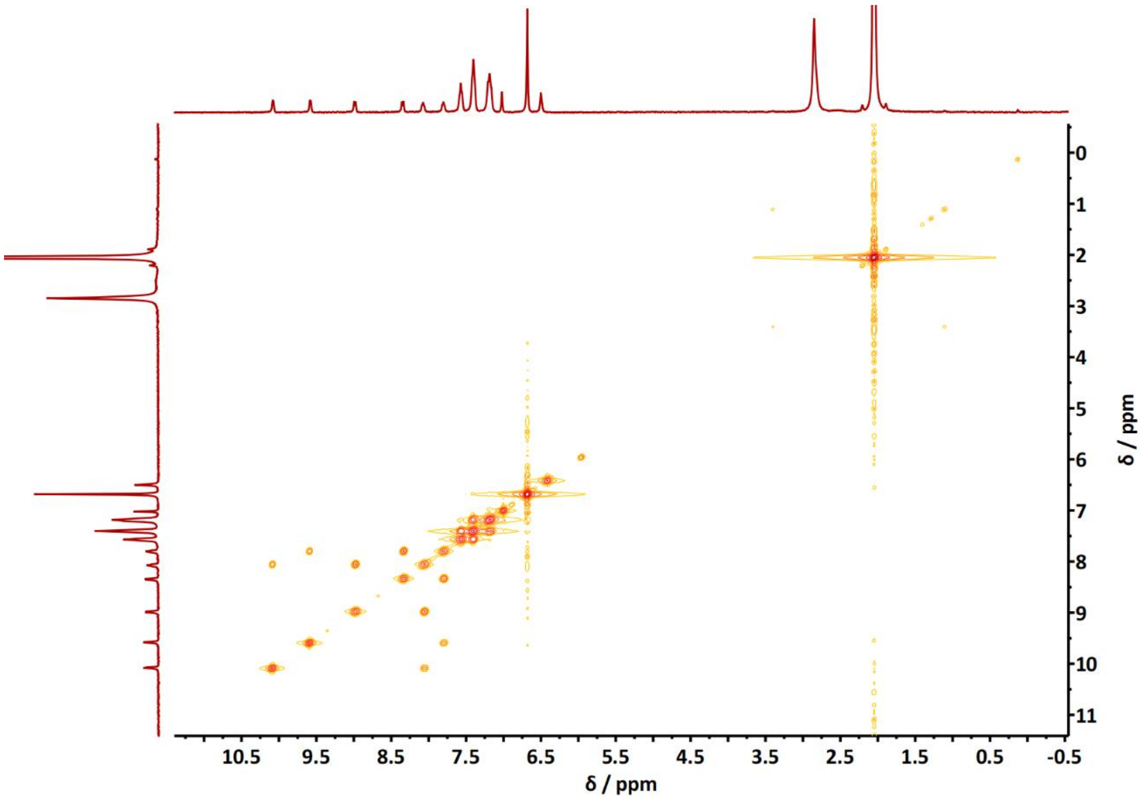

Figure S43. cosy-NMR spectrum of complex $[CoCp(PPh_3)(NH_2Phen)][(CF_3SO_3)_2]$ (**4**) in $(CD_3)_2CO$.

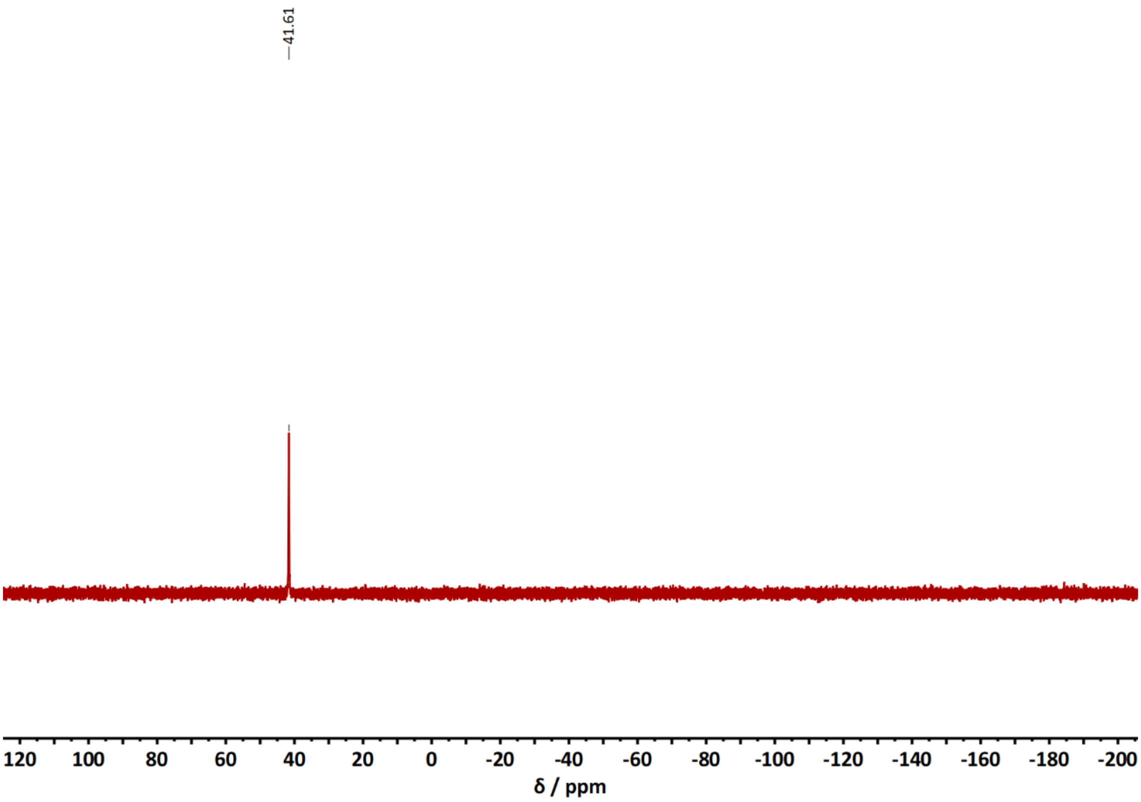

Figure S44. ^{31}P NMR spectrum of complex $[CoCp(PPh_3)(NH_2Phen)][(CF_3SO_3)_2]$ (**4**) in $(CD_3)_2CO$.

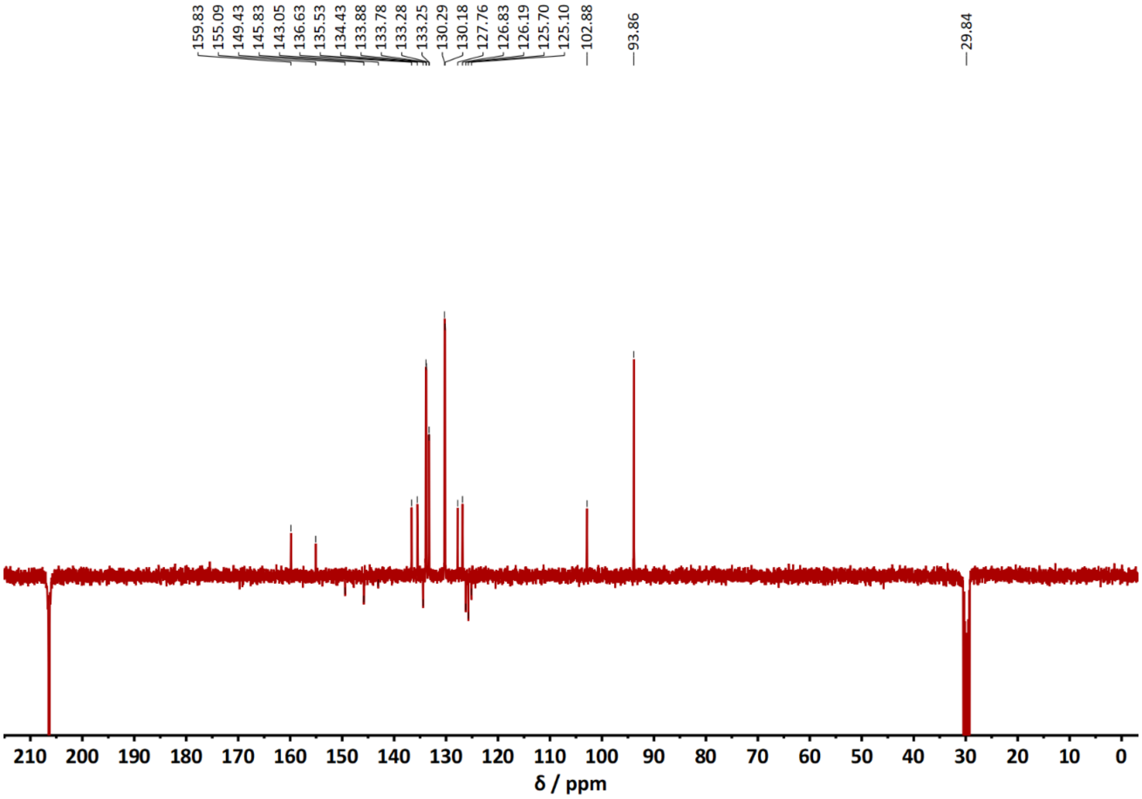

Figure S45. ^{13}C NMR spectrum of complex [CoCp(PPh$_3$)(NH$_2$Phen)][(CF$_3$SO$_3$)$_2$] (**4**) in (CD$_3$)$_2$CO.

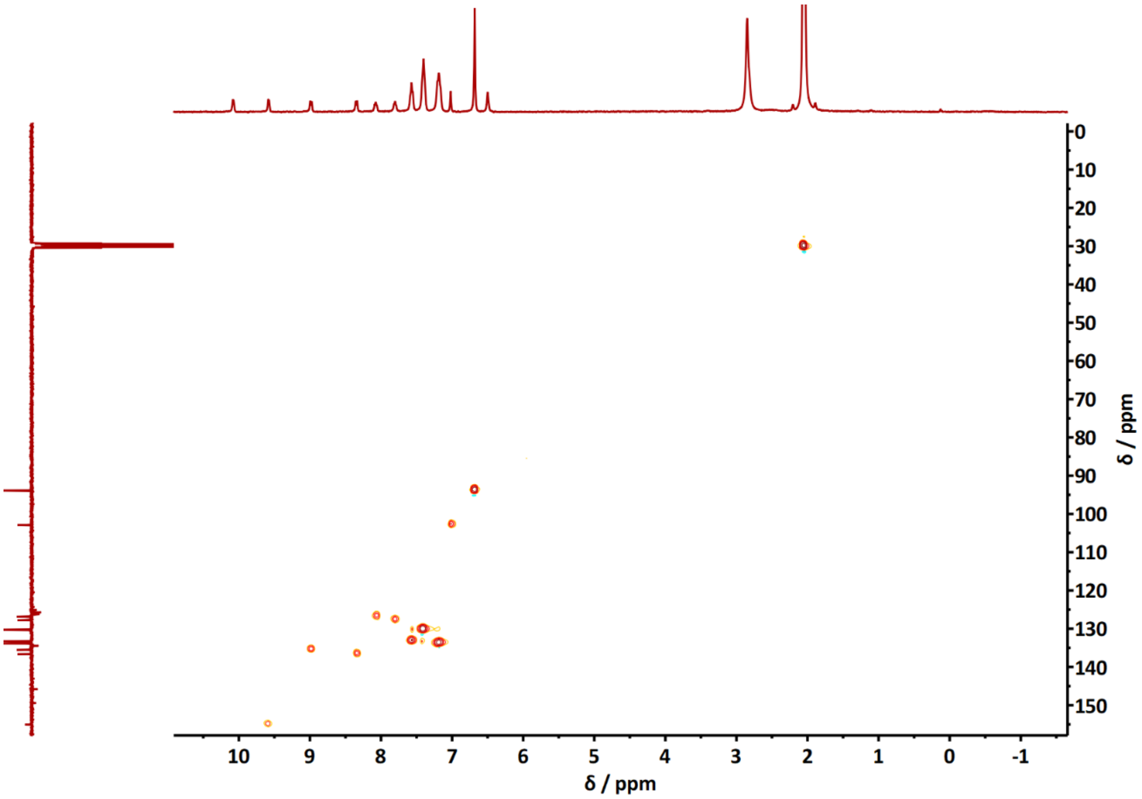

Figure S46. HSQC-NMR spectrum of complex [CoCp(PPh$_3$)(NH$_2$Phen)][(CF$_3$SO$_3$)$_2$] (**4**) in (CD$_3$)$_2$CO.

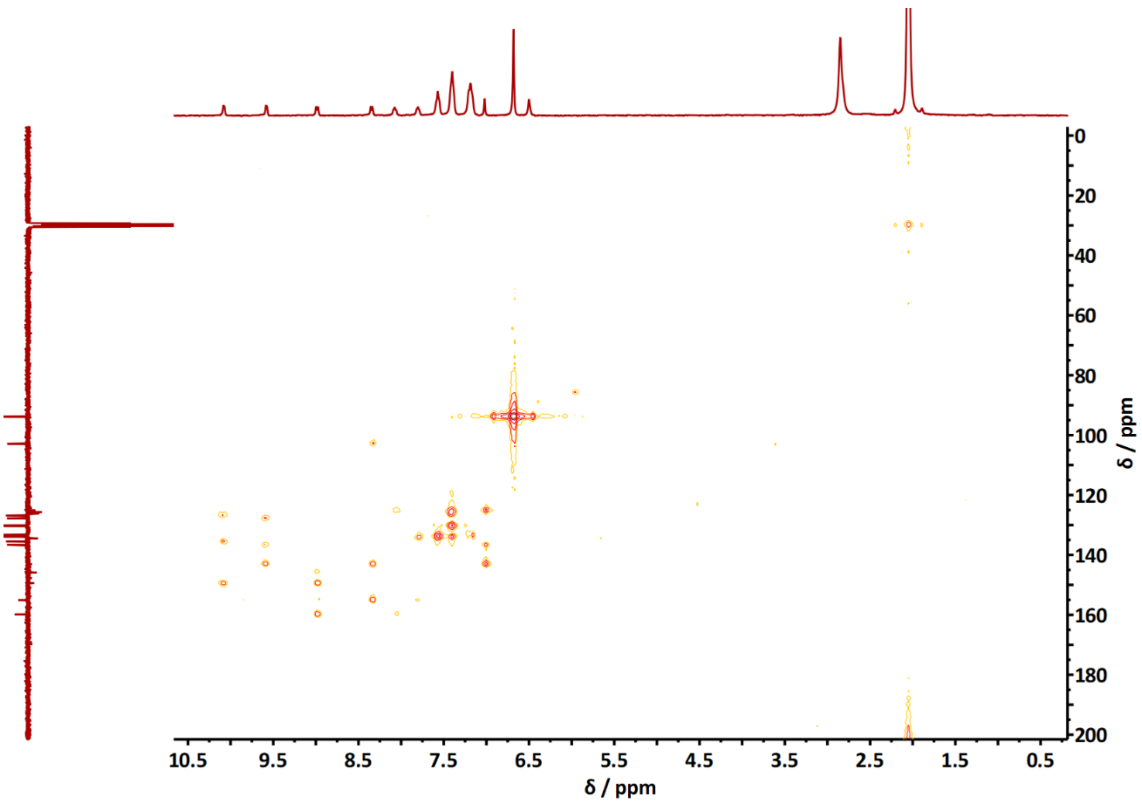

Figure S47. HMBC-NMR spectrum of complex [CoCp(PPh$_3$)(NH$_2$Phen)][(CF$_3$SO$_3$)$_2$] (**4**) in (CD$_3$)$_2$CO.

Supplementary FTIR Data

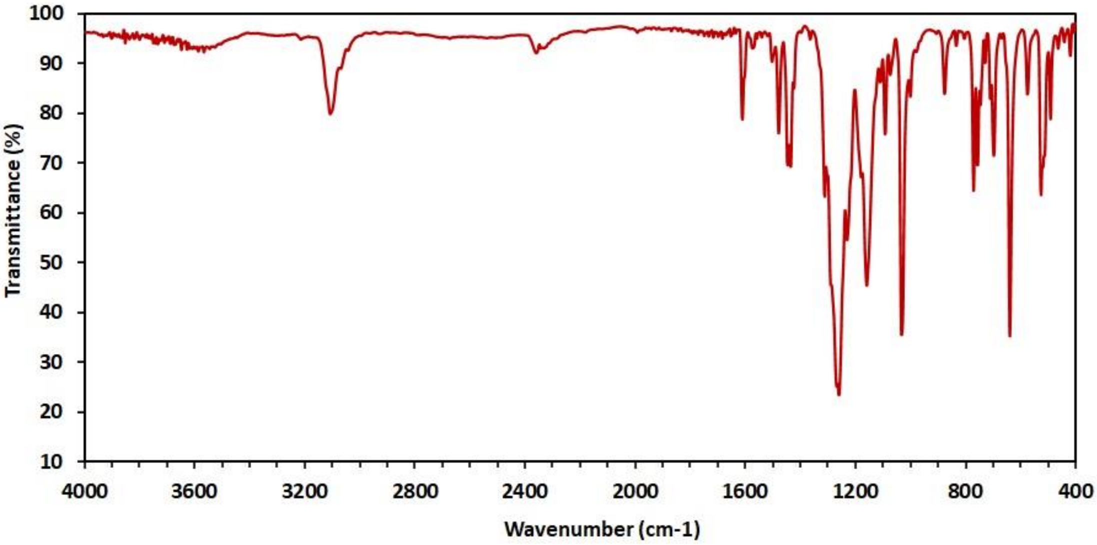

Figure S48. FTIR Spectrum of complex [CoCp(PPh$_3$)(bipy)][(CF$_3$SO$_3$)$_2$] (**1**), KBR.

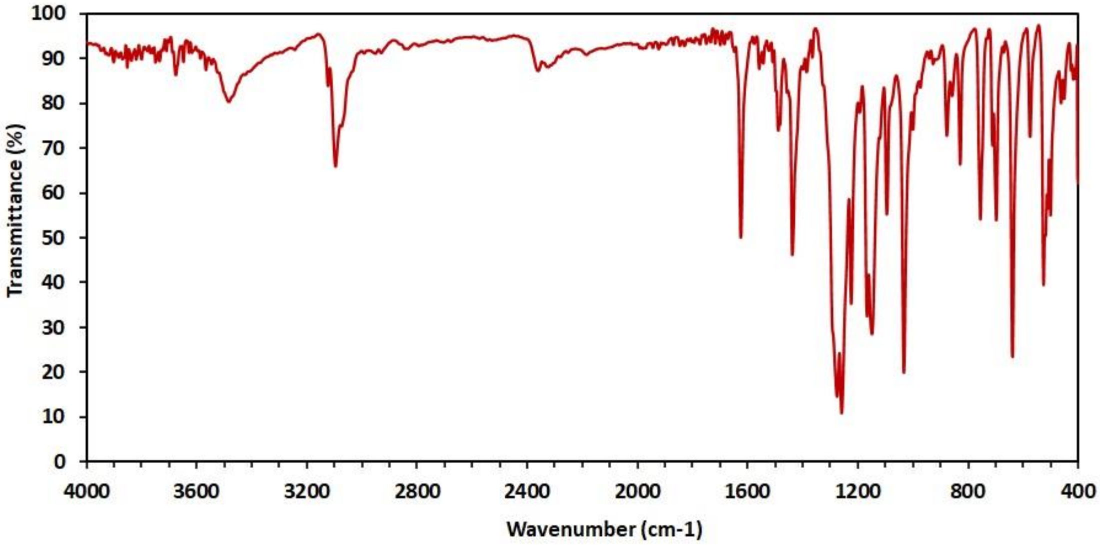

Figure S49. FTIR Spectrum of complex [CoCp(PPh$_3$)(Me$_2$bipy)][(CF$_3$SO$_3$)$_2$] (**2**), KBr.

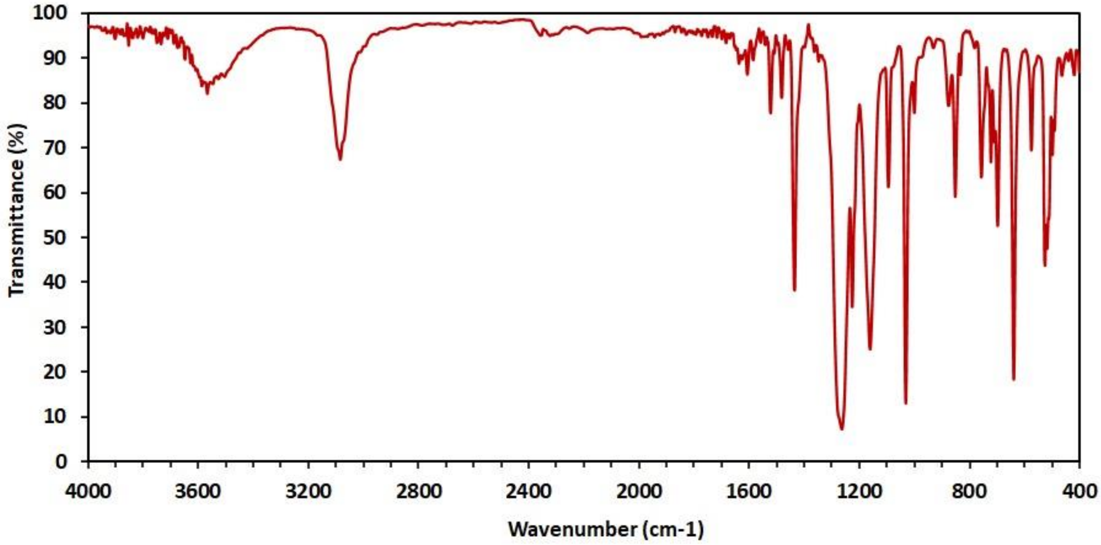

Figure S50. FTIR Spectrum of complex [CoCp(PPh$_3$)(Phen)][(CF$_3$SO$_3$)$_2$] (**3**), KBr.

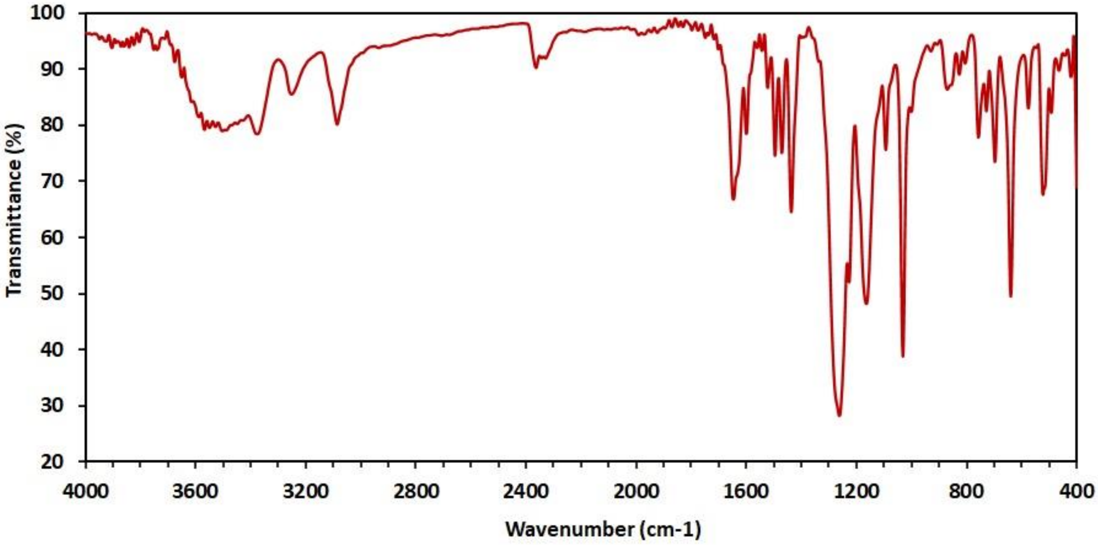

Figure S51. FTIR Spectrum of complex $[CoCp(PPh_3)(NH_2phen)][(CF_3SO_3)_2]$ (**4**), KBr.

Supplementary HPLC

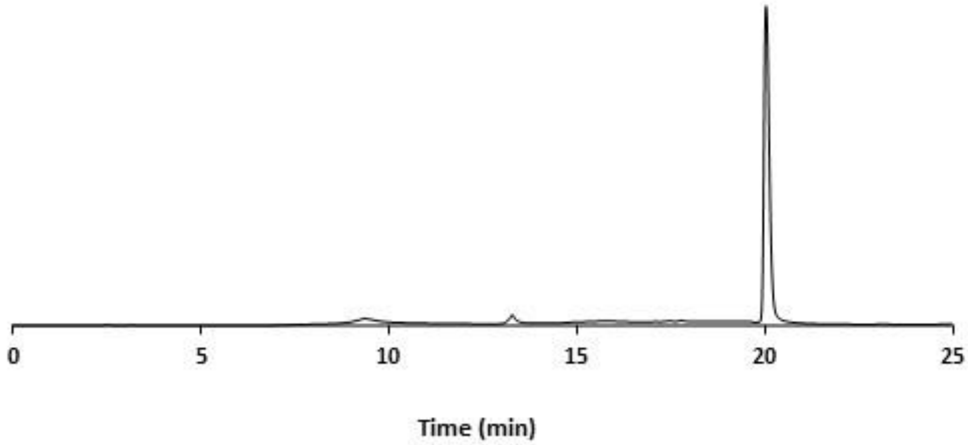

Figure S52. Analytical RP-HPLC chromatogram of complex $[CoCp(PPh_3)(NH_2phen)][(CF_3SO_3)_2]$ (**4**) using method 0-1 min: 10%B; 1-18 min: 10-90% B; 18-21 min: 90% B; 21-24 min: 90-10% B; 24-25 min: 10% B),where A: H2O, B: CAN.

Supplementary UV-Vis. Data

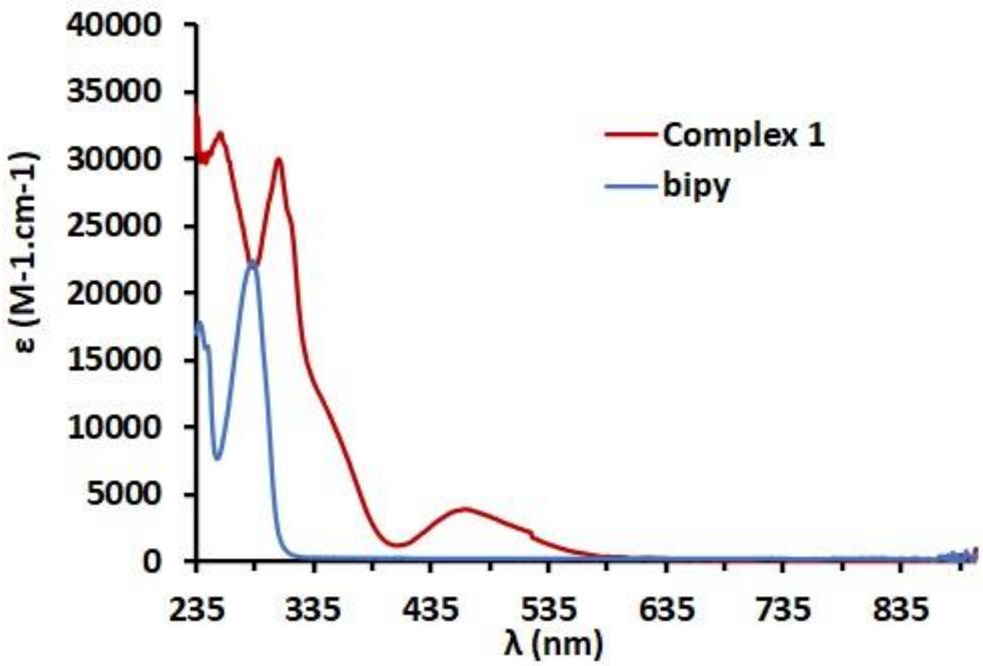

Figure S53. Electronic spectra of $[CoCp(PPh_3)(bipy)][(CF_3SO_3)_2]$ (**1**, —),and free bipy ligand (—) in dichloromethane.

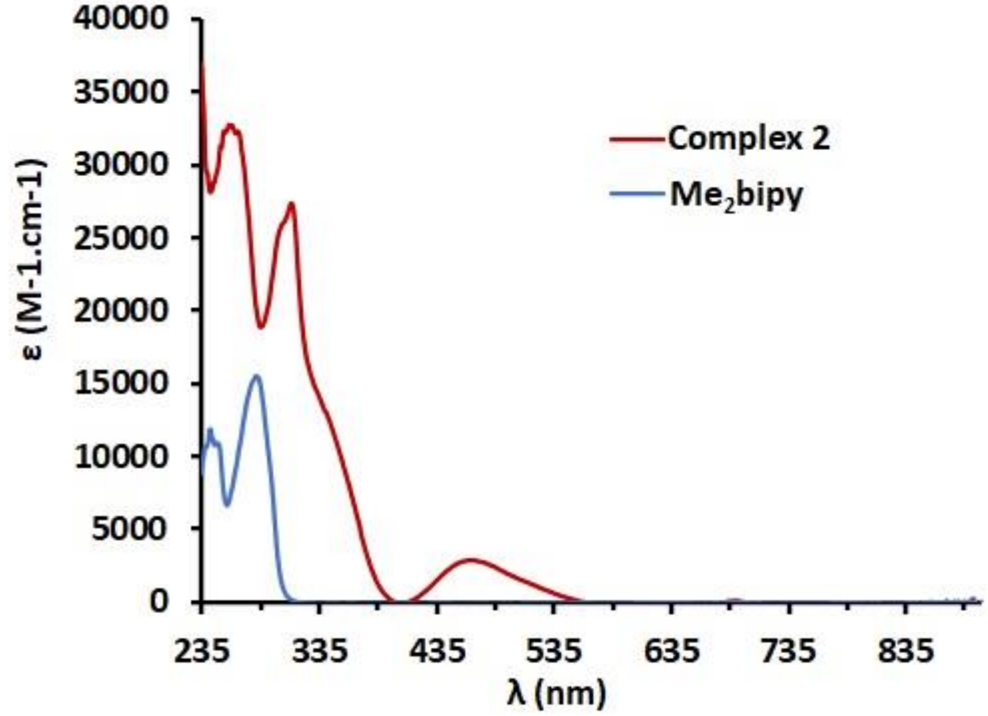

Figure S54. Electronic spectra of [CoCp(PPh$_3$)(Me$_2$bipy)][(CF$_3$SO$_3$)$_2$] (**2**, —),and free Me$_2$bipy ligand (—) in dichloromethane.

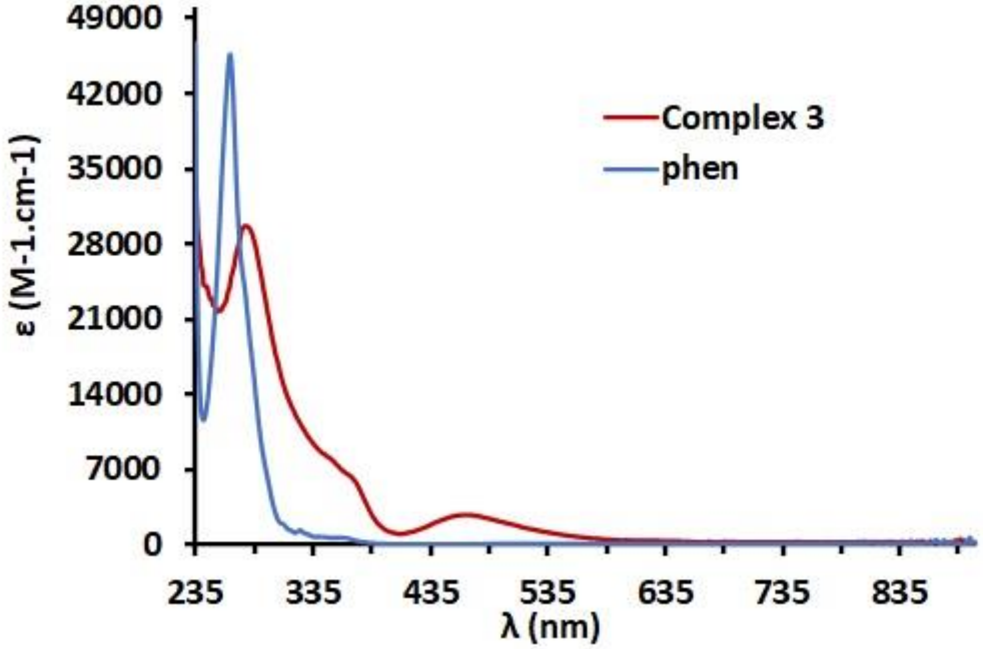

Figure S55. Electronic spectra of [CoCp(PPh$_3$)(Phen)][(CF$_3$SO$_3$)$_2$] (**3**, —),and free Phen ligand (—) in dichloromethane.

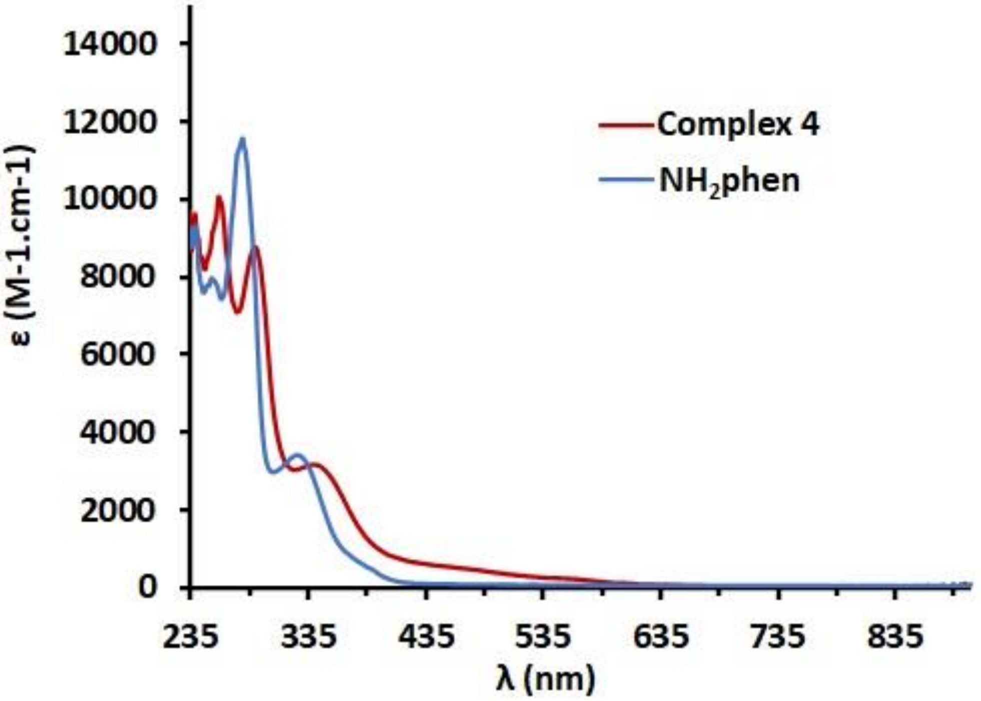

Figure S56. Electronic spectra of $[CoCp(PPh_3)(NH_2Phen)][(CF_3SO_3)_2]$ (**4**, —),and free NH_2Phen ligand (—) in dichloromethane.
